# TEMI: tissue-expansion mass-spectrometry imaging

**DOI:** 10.1038/s41592-025-02664-9

**Published:** 2025-04-22

**Authors:** Hua Zhang, Lang Ding, Amy Hu, Xudong Shi, Penghsuan Huang, Haiyan Lu, Paul W. Tillberg, Meng C. Wang, Lingjun Li

**Affiliations:** 1 School of Pharmacy, University of Wisconsin-Madison, Madison, WI USA; 2 Howard Hughes Medical Institute, Janelia Research Campus, Ashburn, VA USA; 3 Graduate Program in Chemical, Physical & Structural Biology, Graduate School of Biomedical Science, Baylor College of Medicine, Houston, TX USA; 4 Division of Otolaryngology, Department of Surgery, School of Medicine and Public Health, University of Wisconsin-Madison, Madison, WI USA; 5 Department of Chemistry, University of Wisconsin-Madison, Madison, WI USA

**Keywords:** Metabolomics, Mass spectrometry, Molecular imaging, Lipidomics, Lipidomics

## Abstract

The spatial distribution of diverse biomolecules in multicellular organisms is essential for their physiological functions. High-throughput in situ mapping of biomolecules is crucial for both basic and medical research, and requires high scanning speed, spatial resolution, and chemical sensitivity. Here we developed a tissue-expansion method compatible with matrix-assisted laser desorption/ionization mass-spectrometry imaging (TEMI). TEMI reaches single-cell spatial resolution without sacrificing voxel throughput and enables the profiling of hundreds of biomolecules, including lipids, metabolites, peptides (proteins), and N-glycans. Using TEMI, we mapped the spatial distribution of biomolecules across various mammalian tissues and uncovered metabolic heterogeneity in tumors. TEMI can be easily adapted and broadly applied in biological and medical research, to advance spatial multi-omics profiling.

## Abstract (web summary)

Tissue-expansion method compatible with mass-spectrometry imaging (TEMI) enables the profiling of lipids, metabolites, peptides, proteins, and N-glycans in complex tissues with high spatial resolution, advancing spatially resolved multi-omics mapping.

## Main

Multicellular organisms consist of diverse molecular species, including nucleic acids, proteins, lipids, carbohydrates, and metabolites. These molecules actively contribute to organismal physiology and pathology, and their spatial distribution within tissues and across different cell types plays a crucial role in their functions. Advances in next-generation sequencing and spatial transcriptomics have revolutionized the high-throughput analysis of nucleic acids with molecular specificity and spatial precision^1,2^. However, mapping the spatial distribution of other molecular species, such as lipids, metabolites, and glycans, with high spatial resolution and throughput remains technically challenging.

Mass-spectrometry imaging (MSI) has emerged as a powerful technique for spatially mapping molecules in tissue samples in a label-free manner^3–7^. However, its broad application in basic and medical research has been limited by its low spatial resolution. Recent advances in instrumentation have improved MSI spatial resolution^8–11^, but their broad adoption has been limited by prohibitive costs and specialized hardware requirements. To circumvent this challenge, we have developed a tissue-expansion method compatible with mass-spectrometry imaging (TEMI), an easily adaptable and inexpensive tissue-expansion method compatible with MSI.

## Results

### Development of tissue-expansion method for MSI

**Fig. 1:**
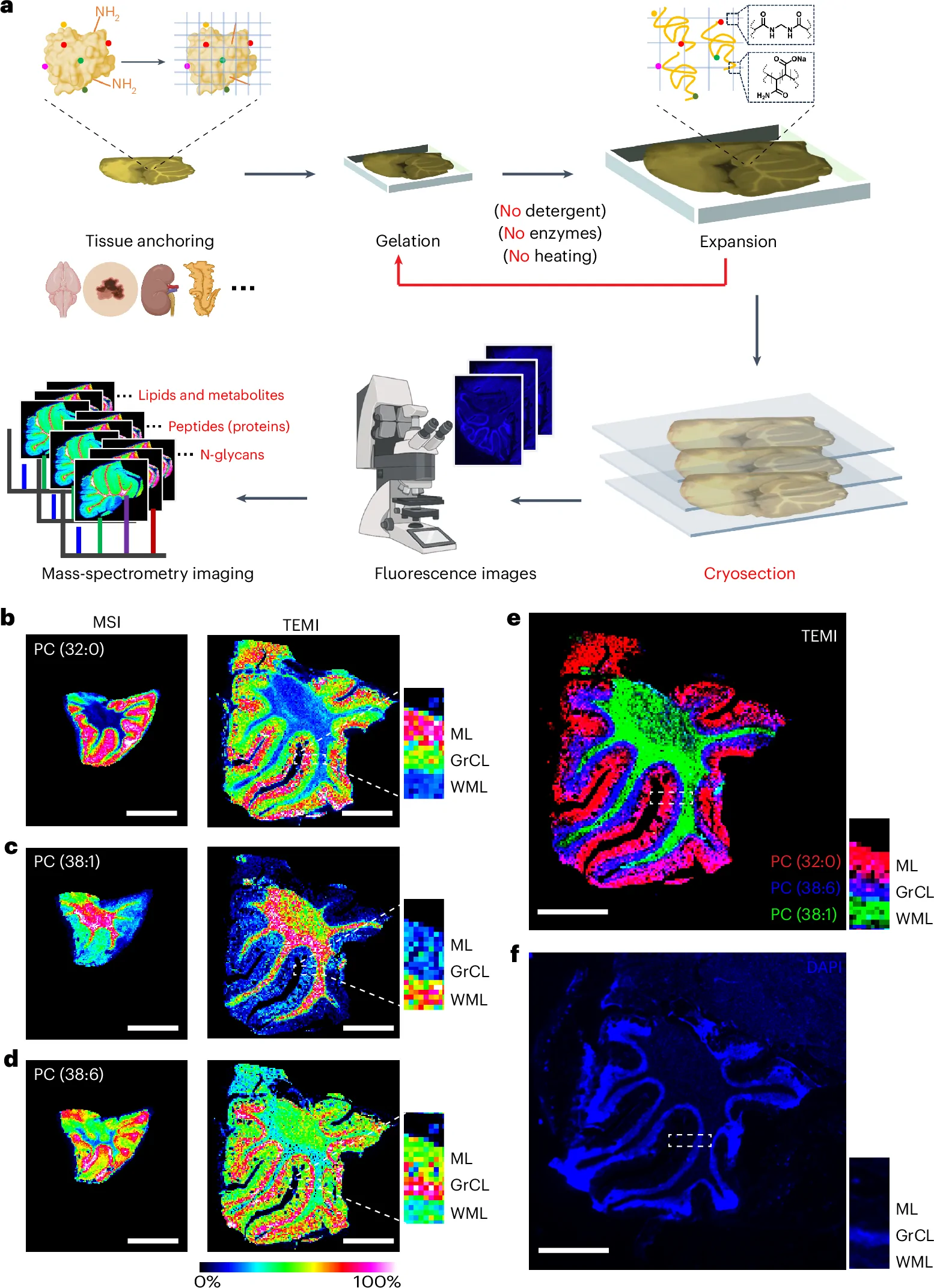
TEMI mapping of lipid distribution cross cerebellar layers. **a**, Concept and workflow of TEMI for mapping biomolecules with improved spatial resolution. The colored dots denote small-molecule metabolites; the blue grid denotes the hydrogel polymers, with chemically anchored proteins. **b**–**d**, Representative MS images of unexpanded control (left, MSI) and double-embedded (right, TEMI) cerebellum using a 50-µm laser beam raster scanning, with lipid species specifically enriched in three distinctive layers. **b**, ML: PC (32:0) ([M+H]^+^, *m/z* 734.568); **c**, WML: PC (38:1) ([M+H]^+^, *m/z* 816.653); **d**, GrCL: PC (38:6) ([M+H]^+^, *m/z* 806.573). **e**, Overlay of TEMI images of PC (32:0) (red), PC (38:1) (green), and PC (38:6) (blue), highlighting the functional layers of cerebellum. **f**, DAPI staining fluorescent image of the expanded cerebellum tissue. White dashed boxes denote regions magnified on the right. All the MSI images were obtained with a mass error tolerance of 10 ppm. Scale bars, 2 mm. Here and throughout, unless otherwise noted, the color bar indicates the ion intensity scale ranging from 0 to 100%. Microscope and mouse tissue shapes in **a** were adapted from BioRender.com.

Tissue expansion typically involves chemically anchoring proteins into a hydrogel synthesized in situ. This is followed by a harsh denaturation process involving high temperatures, detergents, and proteolysis, to enable a uniform swelling of the tissue–hydrogel material^12^, which can expand up to approximately 10-fold linearly when washed with deionized water^13,14^ or approximately 20-fold with sequential embedding rounds^15^. We hypothesized that with no proteolysis, detergent, or high-temperature treatment in tissue expansion, biomolecules, including lipids, protein-associated metabolites, peptides, and N-glycans, would be better retained through interactions with anchored, native-state proteins, which could offer an opportunity to use MSI for high-spatial-resolution mapping of these biomolecules in tissue (Fig. 1a).

We began by optimizing cryosectioning conditions for expansion-embedded tissue, hypothesizing that an uneven layer of hydrogel on the tissue surface could interfere with matrix-assisted laser desorption ionization (MALDI) laser penetrance and ion release. For our experiments, we used mouse cerebellar tissue embedded with a high-monomer, high-toughness gel^14^. We explored different sucrose concentrations and slice thicknesses and found that 30% sucrose yielded optimal results for cryosectioning the expanded tissue–gel material. Other sucrose concentrations did not produce viable slices for MSI. Obtaining intact tissue slices thinner than 30 µm became increasingly difficult (Supplementary Fig. 1). MALDI-MS analysis of the tissue sections indicated that the signal intensities exhibit no notable decline as the thickness increases from 10 to 30 µm (Supplementary Fig. 2).

Conventional tissue-expansion methods that use high temperature or proteolysis have recently been coupled to MSI^16–18^. However, we were concerned that these harsh treatments could compromise performance by causing both signal loss and signal delocalization^16^. To compare overall analyte retention, we extracted lipids from tissue sections that were expanded with and without proteinase K treatment, as well as unexpanded control sections, and then analyzed them using high-performance liquid chromatography–electrospray ionization tandem mass spectrometry (HPLC-ESI-MS/MS). Overall, the detected lipid species were only modestly reduced in the tissue expanded without proteinase K digestion compared with the control, whereas the tissue expanded with digestion exhibited strong signal loss (Supplementary Figs. 3 and 4). We also expanded mouse brain cerebella treated with proteinase K or trypsin digestion, following published methods^17,18^, and obtained only a few crack-free slices after drying that could be used for MSI (Supplementary Fig. 5 and Supplementary Note 1). During MSI experiments, we observed that without post-gel cryosectioning (as has been described recently^17,18^) the expanded tissue was sometimes located beneath an uneven layer of hydrogel, which prevented the MALDI laser beam from efficiently releasing material from the tissue. Moreover, the presence of hydrogel on the tissue surface interfered with the crystallization of analytes and the MALDI matrix, substantialy impacting the MSI results.

When expanding the tissue without denaturation, we found that full expansion in deionized water typically results in cracks or macroscale gel deformation. Thus, we began by limiting the expansion to ~1.5-fold using 1× PBS. Strikingly, even with this limited expansion, MSI with a 50-µm laser raster scan showed more structural features compared with unexpanded controls (Supplementary Fig. 6). These results confirm that denaturation-free expansion effectively enhances spatial resolution while elucidating chemical distribution. They also suggest that TEMI makes it possible to conduct tissue scanning with both high spatial resolution and high voxel throughput. In unexpanded samples, achieving the same effective spatial resolution requires a finer laser beam for raster scanning, which in turn increases acquisition time.

**Extended Data Fig. 1:**
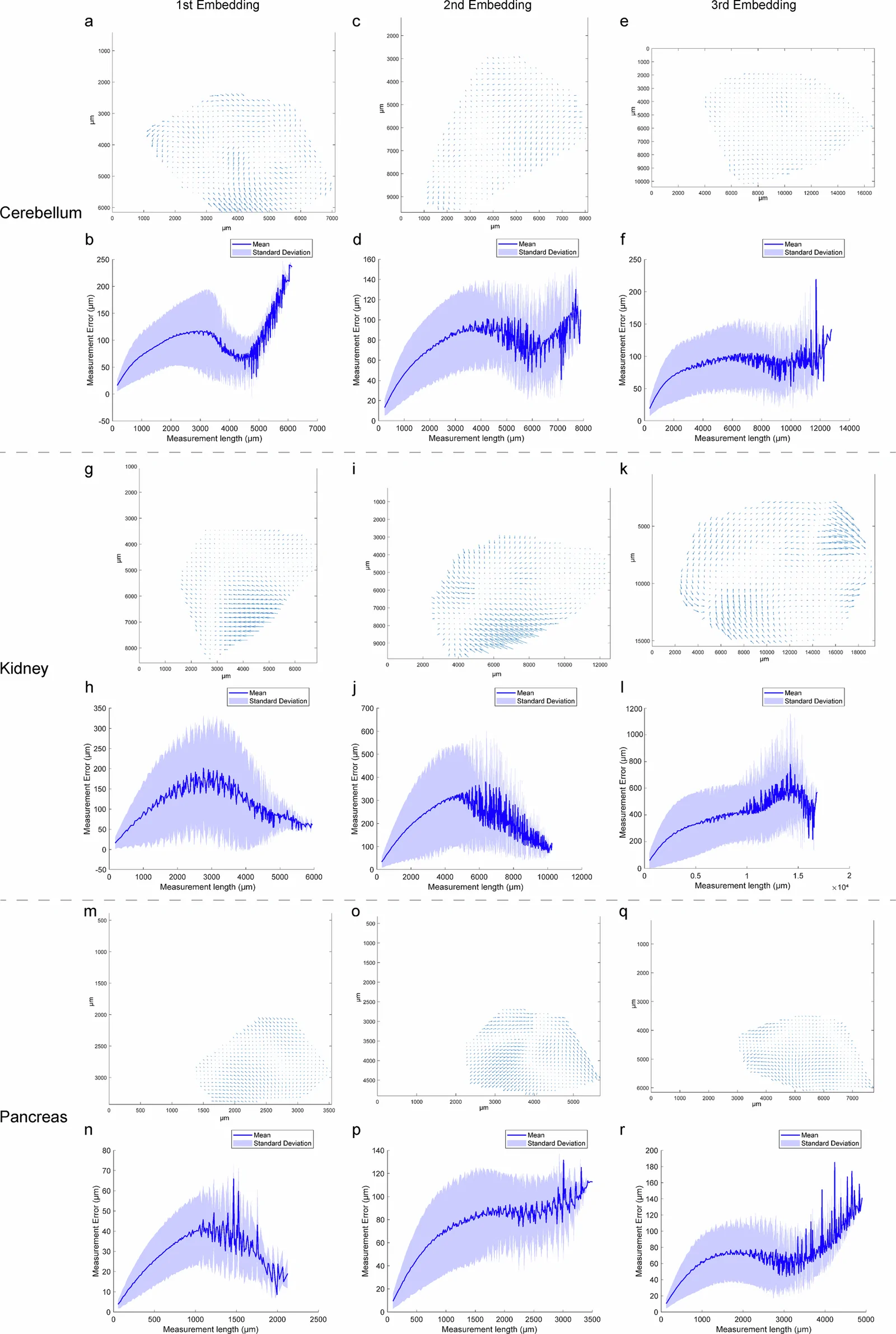
Deformation maps and quantification of measurement errors between the 1st, or 2nd, or 3rd embedding and pre-expansion of different mouse tissue types. Deformation map between: **a**. the 1st embedding and pre-expansion. **c**. the 2nd embedding and pre-expansion. **e**. the 3rd embedding and pre-expansion of cerebellum. **g**. the 1st embedding and pre-expansion. **i**. the 2nd embedding and pre-expansion. **k**. the 3rd embedding and pre-expansion of kidney. **m**. the 1st embedding and pre-expansion. **o**. the 2nd embedding and pre-expansion. **q**. the 3rd embedding and pre-expansion of pancreas. Quantification of measurement error (y-axis, deviation from expected distance assuming isotropic expansion) between pairs of points between: **b**. the 1st embedding and pre-expansion. **d**. the 2nd embedding and pre-expansion. **f**. the 3rd embedding and pre-expansion of cerebellum. **h**. the 1st embedding and pre-expansion, **j**. the 2nd embedding and pre-expansion, **l**. the 3rd embedding and pre-expansion of kidney. **n**. the 1st embedding and pre-expansion. **p**. the 2nd embedding and pre-expansion. **r**. the 3rd embedding and pre-expansion of pancreas for a measurement length in the expanded coordinates (x-axis). Source data

**Extended Data Fig. 2:**
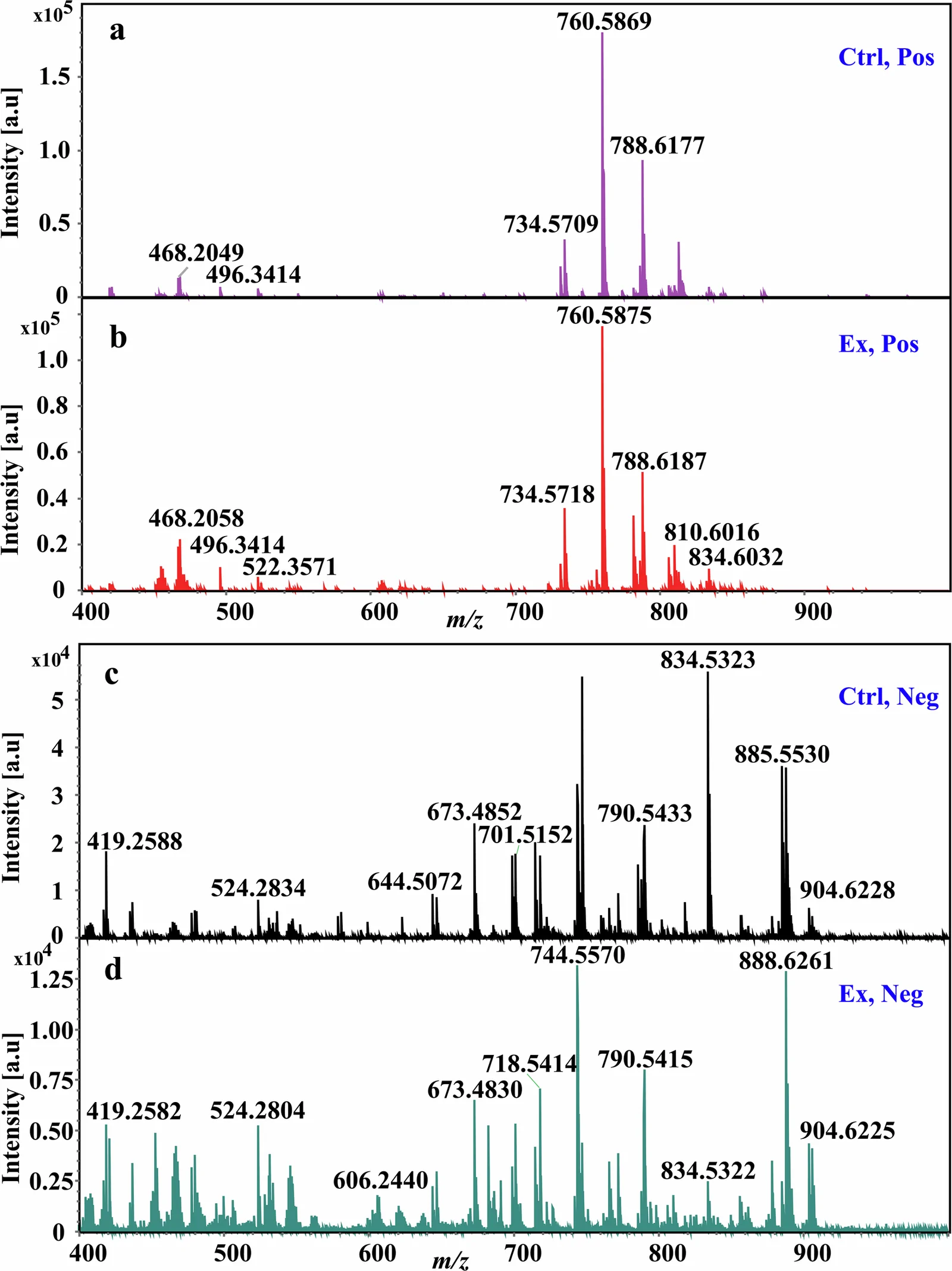
MALDI-MS spotting mass spectra from the corresponding area on control (Ctrl, unexpanded) and expanded (Ex, ~ 2.5-fold linear) mouse brain cerebellar tissue under positive (Pos) and negative (Neg) ionization mode. **a**. mass spectra of matched control tissue under positive mode; **b**. mass spectra of expanded tissue under positive mode; **c**. mass spectra of adjacent control tissue under negative mode; **d**. mass spectra of expanded tissue under negative mode.

We next reasoned that re-embedding the gelled tissue with another round of gelation might produce an interpenetrating gel network with the toughness required to expand non-denatured tissue isotropically down to the micrometer-scale features required for this application. After two rounds of gel embedding, mouse cerebellar slices expanded ~2.5-fold in 1× PBS. We observed that the expanded gels had some residual curvature but could easily be laid flat before freezing without macroscopic tissue distortion (Extended Data Fig. 1a–d and Supplementary Note 2). Expanded cerebellar tissue sections with anatomically matched unexpanded controls were imaged with 1,5-diaminonaphthalene (DAN) matrix. MALDI-MS spotting experiments showed comparable mass spectral patterns of lipid molecules from the corresponding areas on the control and expanded tissue slides in both positive and negative ionization modes (Extended Data Fig. 2). The expanded samples showed a clean background without contaminant signals from the hydrogel material (Supplementary Fig. 7). Notably, MS signal intensities were decreased slightly in the expanded tissue samples, which could be attributed to smaller effective voxels containing fewer analytes. In addition, targeted lipid signals were exclusively detected in the expanded tissue area, with no corresponding signal observed in the adjacent blank hydrogel area (Supplementary Fig. 8), indicating a lack of chemical delocalization in the expansion sample.

### Lipid distribution specificity across cerebellar layers assessed using TEMI

Non-denatured, ~2.5-fold-expanded cerebellar sections were next imaged with a 50-µm raster laser scan with dual-polarity ionization on the same tissue, for an effective lateral resolution of ~20 µm. Strikingly, the molecular layer (ML), granular cell layer (GrCL), and white matter layer (WML) were more clearly distinguished in each cerebellar folium compared with the unexpanded control sample (Fig. 1b–f and Supplementary Figs. 9 and 10). Furthermore, leveraging this enhanced spatial resolution, we identified lipid molecules that were specifically enriched in different functional layers, such as phosphatidylcholine (PC (32:0)), PC (38:1), and PC (38:6) for ML, GrCL, and WML, respectively (Fig. 1b–d). This enabled us to reveal the spatial biomolecular heterogeneity of the cerebellum in conjunction with its structural organization (Fig. 1e,f) and to uncover previously unknown lipid fingerprints for each functional layer (Supplementary Figs. 9–12 and Supplementary Tables 1 and 2).

**Fig. 2:**
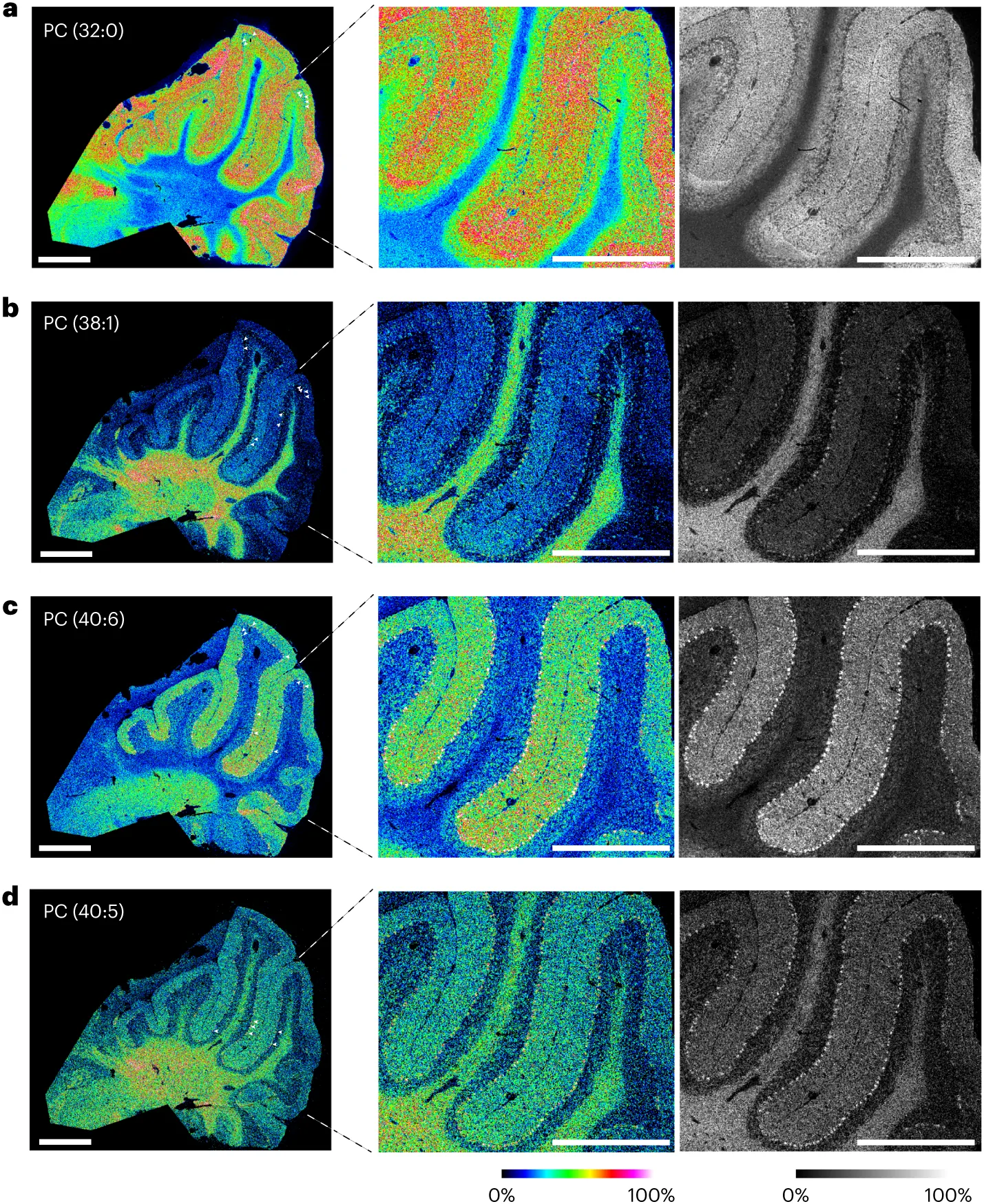
Single-cell-resolution TEMI mapping of Purkinje cells. **a**–**d**, Representative TEMI images of mouse cerebellum tissue expanded by ~3.5 fold, acquired through a 10-µm laser beam raster scanning in positive mode. **a**, PC (32:0) ([M+H]^+^, *m/z* 734.5747). **b**, PC (38:1) ([M+H]^+^, *m/z* 816.647). **c**, PC (40:6) ([M+H]^+^, *m/z* 834.603). **d**, PC (40:5) ([M+H]^+^, *m/z* 836.617). All the MSI images were obtained with a mass error tolerance of 10 ppm. Arrow heads highlight several Purkinje cells as examples. Dashed lines denote the regions magnified to the right, including corresponding colored and grayscale images. Scale bars, 2 mm.

**Extended Data Fig. 3:**
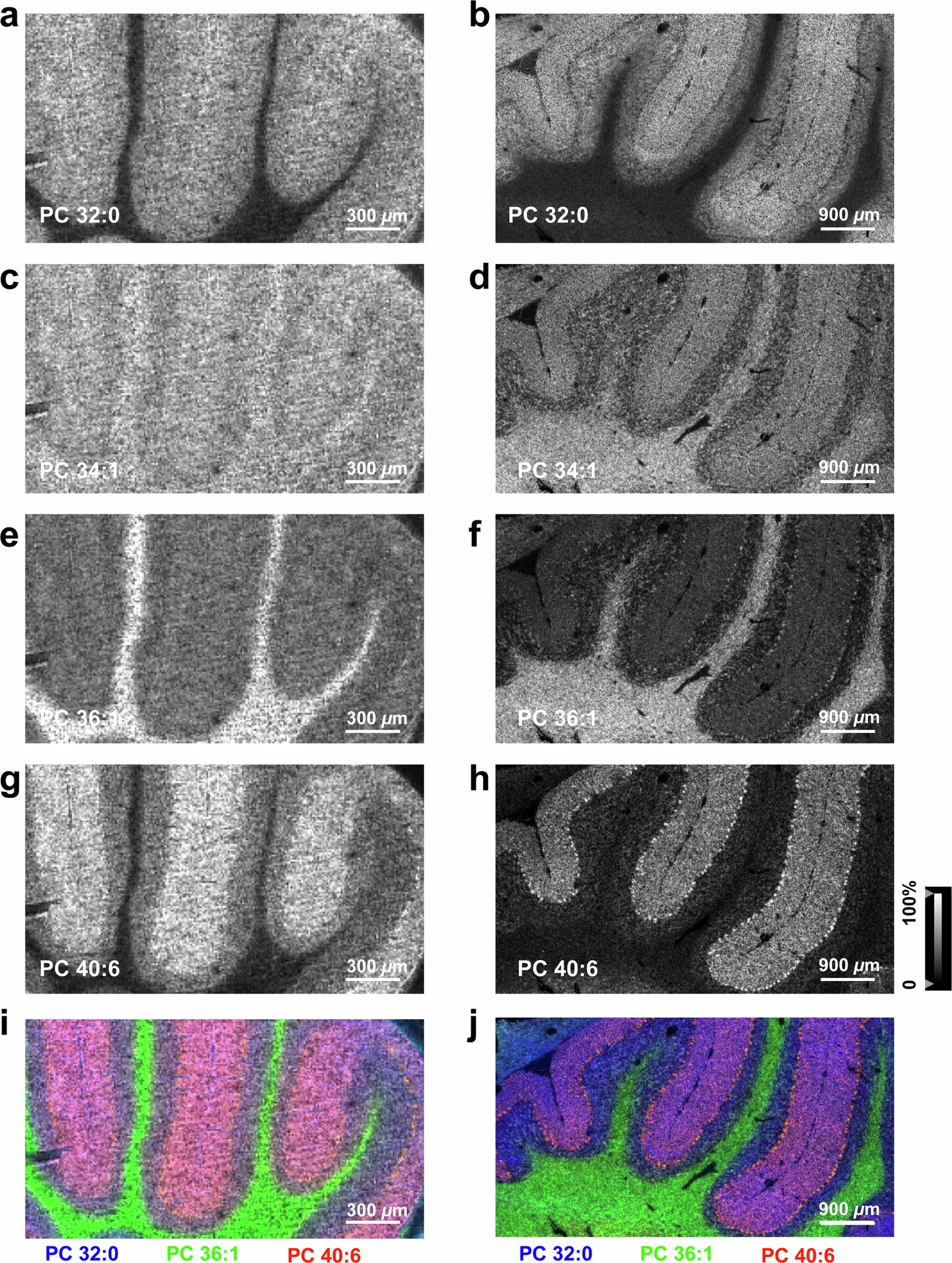
High spatial resolution TEMI of mouse cerebellum after 3 cycles of gel-embedding-expansion. The experiments were carried out using a 10 *µ*m laser beam raster scanning for both unexpanded control tissue (left: **a**, **c**, **e**, **g**, and **i**) and expansion treated tissue (right: **b**, **d**, **f**, **h**, and **j**), including **a** and **b**. For PC (32:0) ([M + H]^+^, *m/z* 734.575); **c** and **d**. For PC (34:1) ([M + H]^+^, *m/z* 760.587); **e** and **f**. For PC (36:1) ([M + H]^+^, *m/z* 788.621); **g** and **h**. For PC (40:6) ([M + H]^+^, *m/z* 834.603); overlapped ion images of PC (32:0) (in blue), PC (36:1) (in green), and PC (40:6) (in red) from the unexpanded control (**i**) and 3 cycles gel-embedding-expansion treated mouse cerebellum (**j**). The MSI images were obtained with mass error tolerance of 10 ppm.

We further explored TEMI’s potential to improve spatial resolution. Cerebellar slices were expanded through three rounds of embedding without denaturation, for a final expansion factor of ~3.5-fold. Imaging these tissue sections with a 10-µm raster laser scan, resulting in a ~2.9-µm effective scanning resolution, revealed greater detail compared with the ~2.5-fold expansion (Fig. 2 and Extended Data Fig. 3). At this resolution, individual Purkinje cells became visible (Fig. 2), revealing an enrichment of specific lipid species, such as PC (38:1), PC (40:5), PC (40:6); however, other lipid species, such as PC (32:0) and PC (34:1), were absent in these cells (Supplementary Table 3). By contrast, the individual Purkinje cells were barely distinguishable in the unexpanded control sample when using conventional MSI with the same laser beam scan (Extended Data Fig. 3).

### TEMI profiling of metabolite distribution in cerebella

**Fig. 3:**
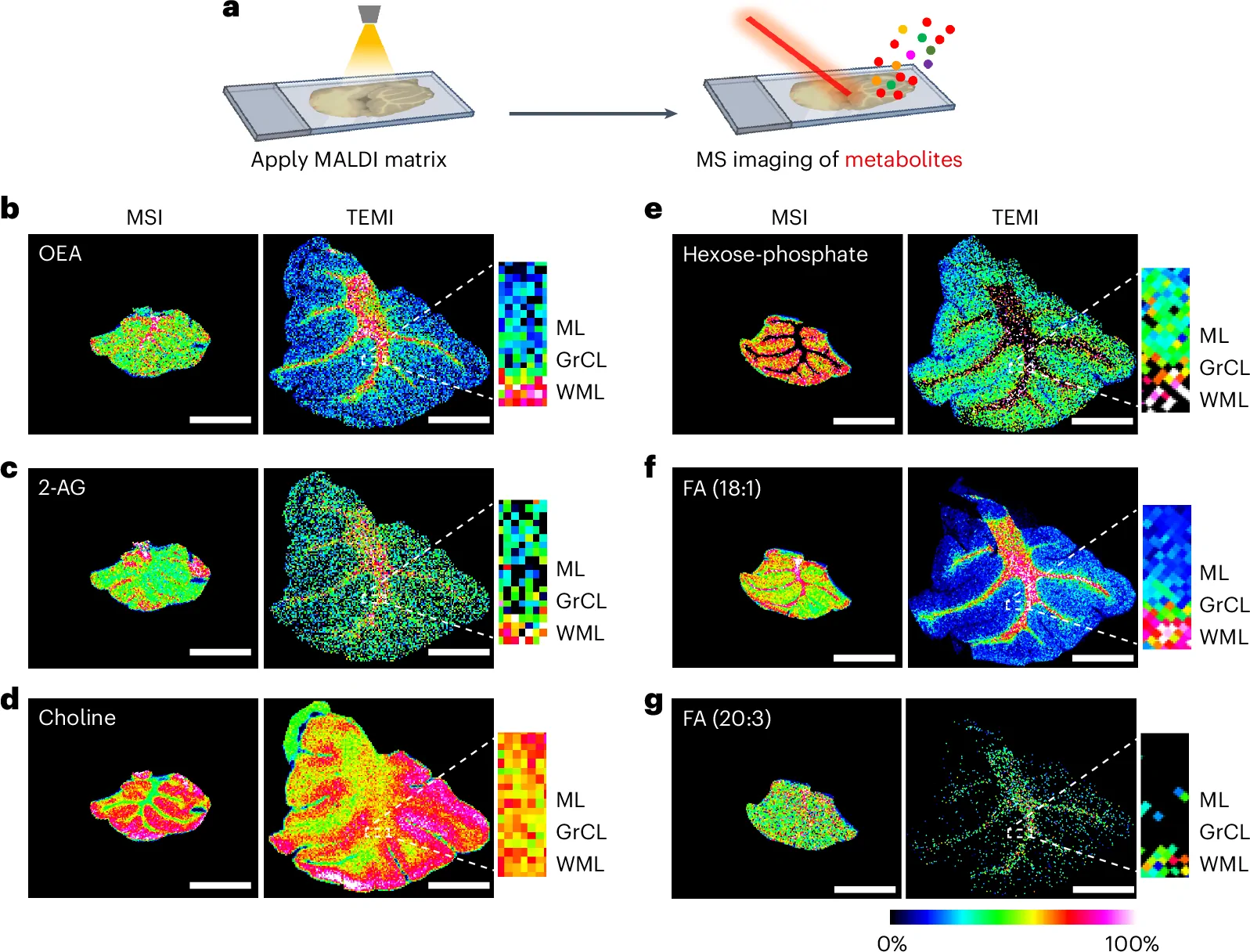
TEMI mapping of small metabolites in mouse cerebellum. **a**, Schematic workflow for detecting metabolites in MSI. **b**–**g**, Representative MS images of the unexpanded control (left, MSI) and double-embedded (right, TEMI) cerebellum in positive (**b**–**d**) and negative (**e**–**g**) ionization modes, with these small molecules specifically enriched in three distinctive layers: ML, GrCL, and WML. **b**, OEA: [M–H_2_O+H]^+^, *m/z* 308.296, WML. **c**, 2-AG: [M–H_2_O+H]^+^, *m/z* 361.277, WML. **d**, Choline: [M+H]^+^, *m/z* 104.107, ML. **e**, Hexose-phosphate: [M–H]^−^, *m/z* 259.022, ML. **f**, Fatty acid 18:1 (FA (18:1)): [M–H]^−^, *m/z* 284.249, WML. **g**, FA (20:3): [M–H]^−^, *m/z* 305.248, WML. The MSI experiments were carried out using a 50-µm laser raster scanning, and the MSI images were obtained with mass error tolerance of 10 ppm. Scale bars, 2 mm.

**Extended Data Fig. 4:**
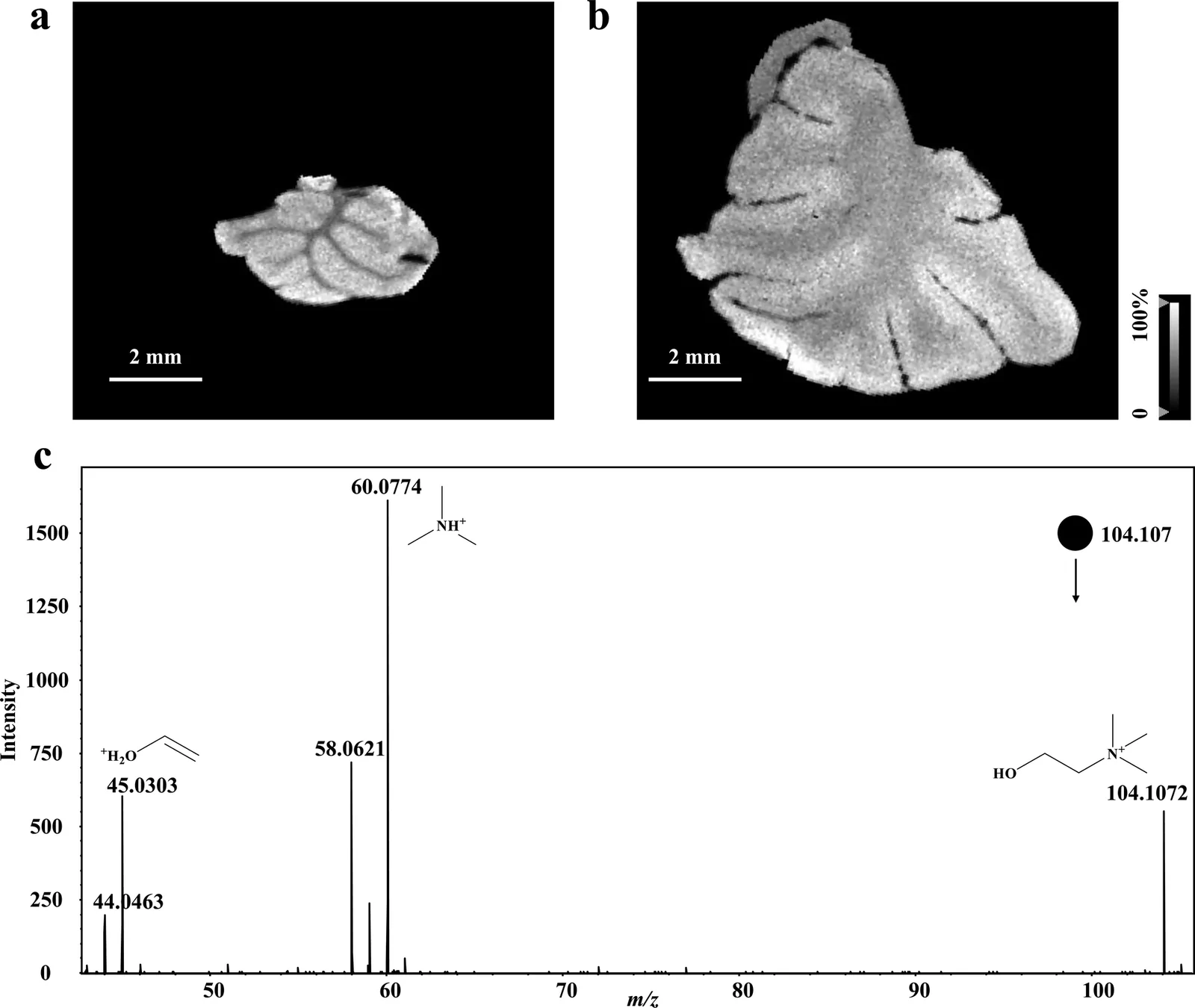
Gray MS images of choline (*m/z* 104.1072) from the unexpanded control and double-embedded cerebellum under positive mode. **a**. MS images of choline from the unexpanded control (MSI); **b**. MS images of choline from double-embedded cerebellum (TEMI); **c**. *In situ* MALDI-MS/MS of target ion of *m/z* 104.1072 from the expanded mouse cerebellum, which confirms the identification of Choline.

In addition to glycolipids and phospholipids, we also imaged metabolites in the mouse cerebella, which were expanded ~2.5-fold linearly. We applied 2,5-dihydroxybenzoic acid and *N*-(1-naphthyl)ethylenediamine dihydrochloride matrix for the positive and negative modes, respectively (Fig. 3a). Among the 187 detected metabolite features, some exhibited distinctive spatial organization among the ML, GrCL, and WML, which were not discernible in the unexpanded controls (Fig. 3b–g and Supplementary Figs. 13 and 14). This result further supports the spatial metabolic heterogeneity of the cerebellum in conjunction with its structural organization. Although we could annotate certain metabolites with their chemical identities, such as choline, hexose-phosphate, oleoylethanolamide (OEA), and 2-arachidonoylglycerol (2-AG) (Supplementary Fig. 15 and Extended Data Fig. 4), fully and accurately annotating all detected metabolites is difficult owing to the lack of a reference library based on MALDI-MSI.

### Mapping cerebellar proteins and N-glycans using TEMI

**Fig. 4:**
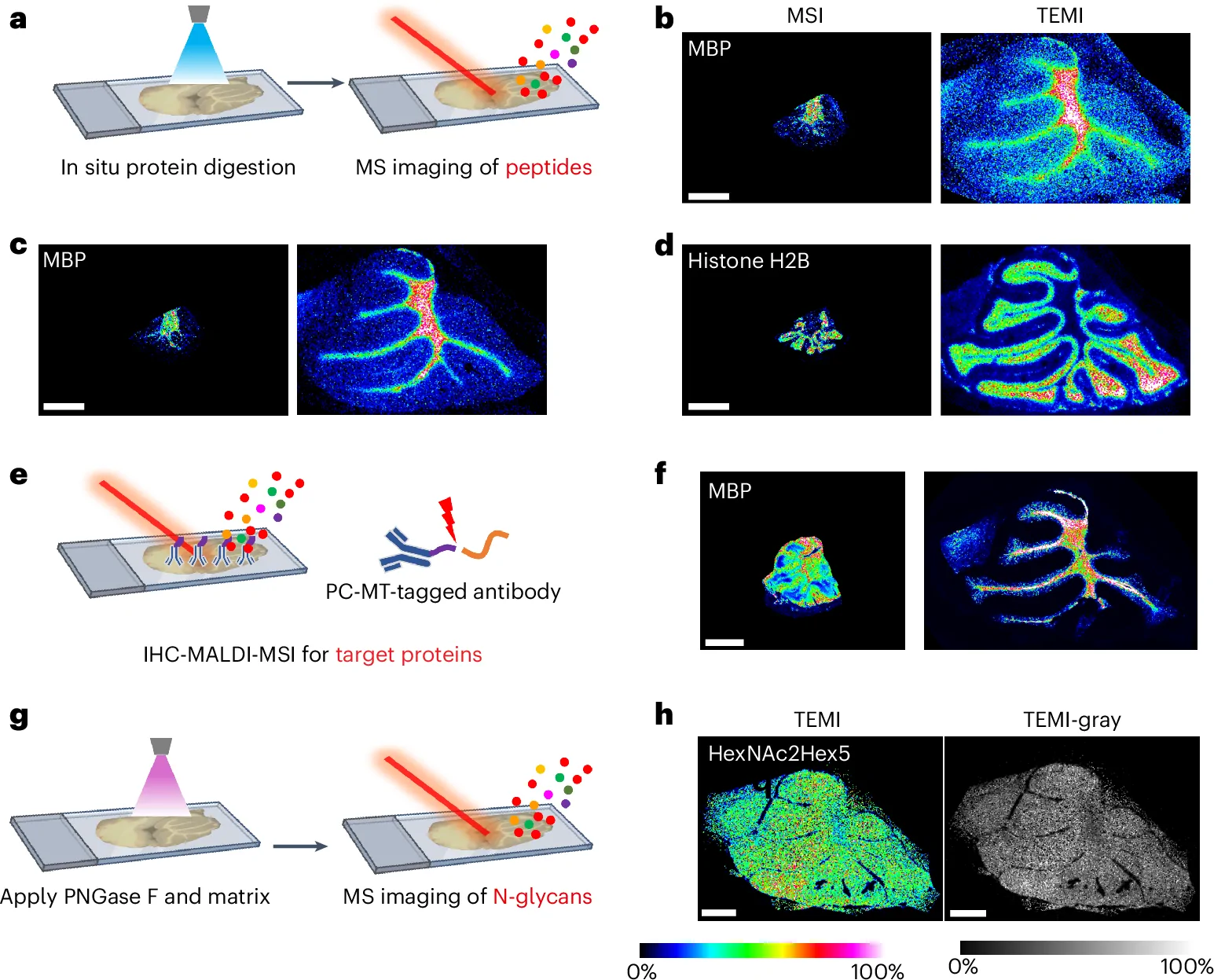
TEMI mapping of proteins and N-glycans in mouse cerebellum. **a**, Schematic of MSI tryptic peptides using in situ trypsin digestion of expanded tissue sections. **b**, Imaging of a peptide (sequence, TTHYGSLPQKSQHGR) from the MBP ([M+H]^+^, *m/z* 1696.856). **c**, Imaging of a peptide (sequence, HRDTGILDSIGRFFSGDRGAPK) from the MBP ([M+H]^+^, *m/z* 2402.242). **d**. Imaging of a peptide (sequence, E[+14]VQTAVRLLLPGELAK) from the histone H2B protein group ([M+H]^+^, *m/z* 1751.045). **e**, A schematic of the workflow for detecting proteins using IHC-MALDI-MSI. **f**, The distribution of MBP in the unexpanded and TEMI samples revealed by IHC-MALDI-MSI ([M+H]^+^, *m/z* 1366.042). **g**, A schematic of in situ N-glycans release using PNGase F for MSI. **h**, TEMI ion image of N-glycan HexNAc2Hex5 ([M+H]^+^, *m/z* 1257.429). The MSI images were obtained with mass error tolerance of 10 ppm. Scale bars, 2 mm.

**Extended Data Fig. 5:**
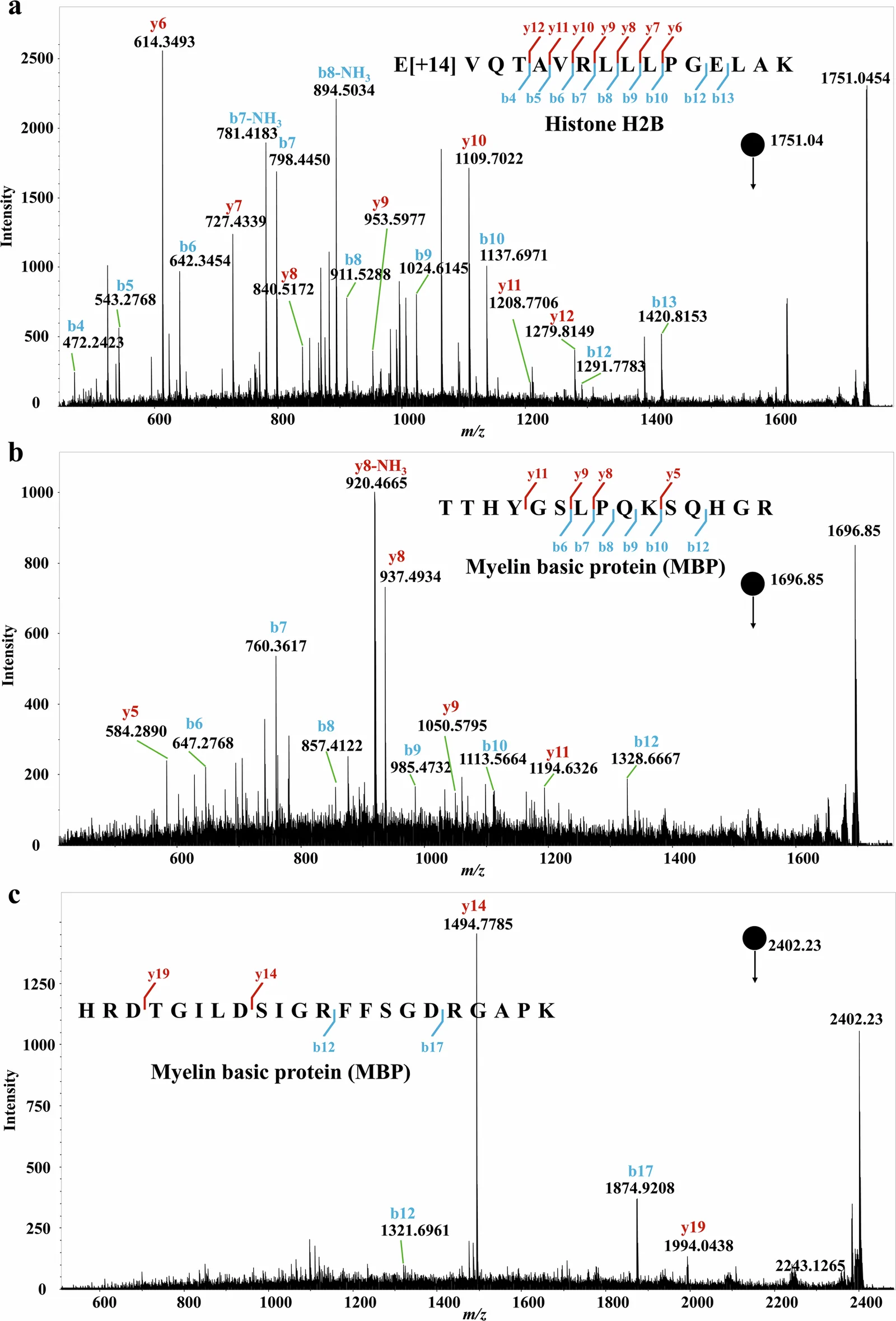
Representative tandem MS spectra of on-tissue tryptic peptides validation via *in situ* MALDI-MS/MS on the expanded mouse cerebellum. **a**. Annotated MALDI-MS/MS spectrum of precursor *m/z* 1751.04 that was acquired directly from the expanded tissue and identified as peptide E[ + 14]VQTAVRLLLPGELAK from Histone H2B, the E[ + 14] modification is caused by the Formaldehyde fixing; **b**. Annotated MALDI-MS/MS spectrum of precursor *m/z* 1696.85 and identified as peptide TTHYGSLPQKSQHGR from MBP; **c**. Annotated MALDI-MS/MS spectrum of precursor *m/z* 2402.23 and identified as peptide HRDTGILDSIGRFFSGDRGAPK from MBP.

We also imaged peptides, proteins, and N-glycans using TEMI in the mouse cerebella expanded ~3.5-fold linearly. To image proteins, we applied in situ tryptic digestion of both unexpanded and expanded tissue sections to release peptides for MSI and ultra-HPLC-nano-ESI-MS/MS proteomic profiling. Using the proteomics results and in situ MALDI–tandem mass spectrometry (Supplementary Table 4 and Extended Data Fig. 5), we identified 57 features within a mass tolerance of 5 ppm, including peptides from the myelin basic protein group (MBP), histone H2B protein group (H2B), and other proteins (Fig. 4b–d and Supplementary Fig. 16). Notably, these proteins exhibited distinctive distributions, with histone H2B protein groups enriched in the GrCL, whereas MBP was enriched in the WML.

Furthermore, we used immunohistochemistry (IHC) photocleavable mass-tag (PC-MT)-tagged antibodies on both unexpanded and TEMI samples (Fig. 4e). IHC-MALDI-MSI confirmed the distinctive pattern of MBP, as well as other proteins revealed by bottom-up tryptic digestion, in the TEMI sample (Fig. 4f and Supplementary Fig. 17). In parallel, we successfully detected up to 29 N-glycans in both unexpanded and TEMI samples (Fig. 4g,h, Supplementary Fig. 18, and Supplementary Table 5). Although most N-glycans are ubiquitously distributed, TEMI revealed that certain ones, such as HexNAc2Hex5, exhibited a moderate degree of regional distinctiveness (Fig. 4h).

### Revealing high metabolic heterogeneity in tumors using TEMI

**Fig. 5:**
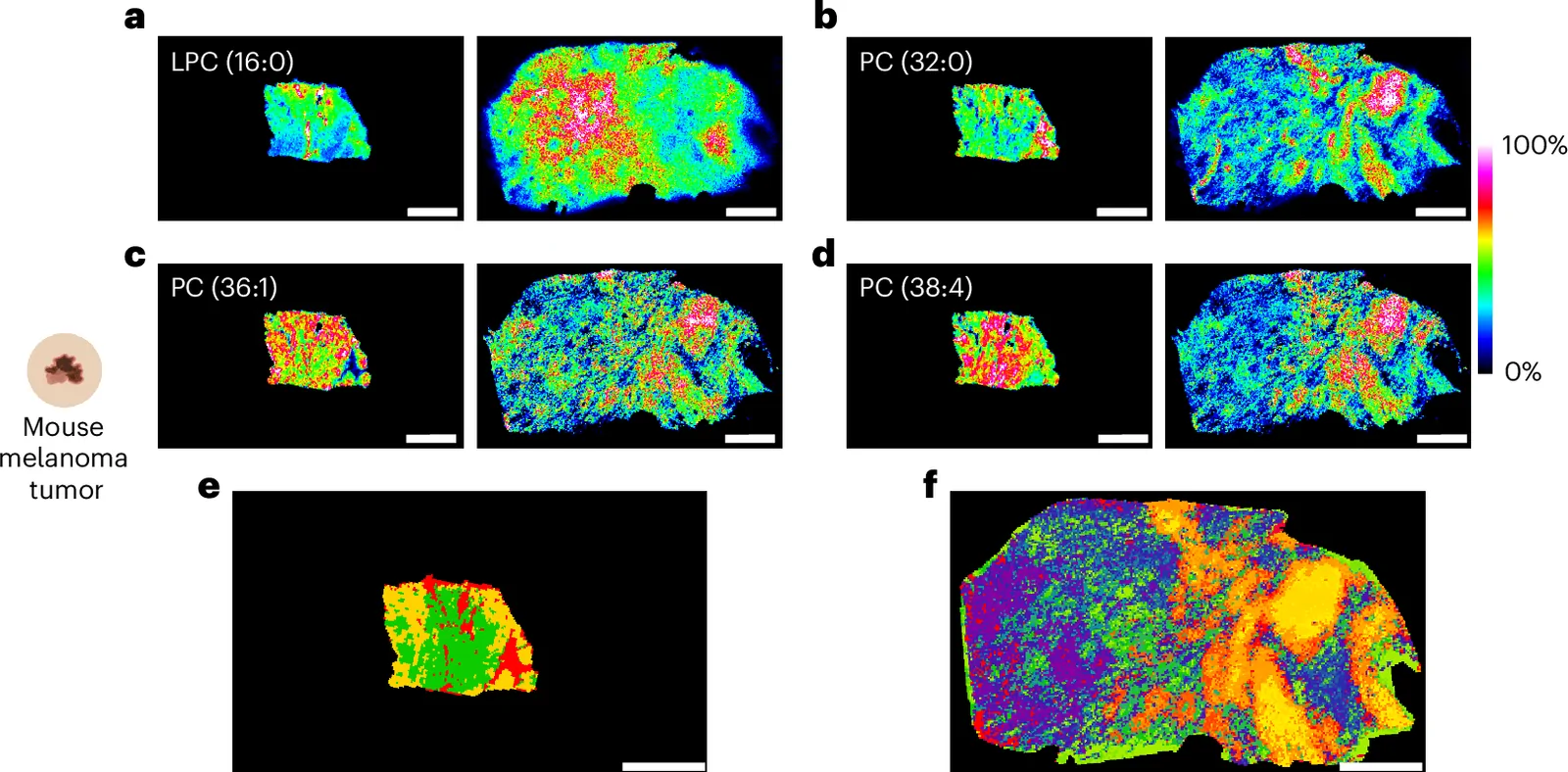
Revealing metabolic heterogeneity in mouse tumor with TEMI. **a**–**f**, MS images of representative lipid species detected from unexpanded control (MSI, left) and expanded tumor tissue (TEMI, right), including LPC (16:0) ([M+H]^+^, *m/z* 496.340) (**a**), PC (32:0) ([M+H]^+^, *m/z* 734.568) (**b**), PC (36:1) ([M+H]^+^, *m/z* 788.614) (**c**), and PC (38:4) ([M+H]^+^, *m/z* 810.598) (**d**). **e**,**f**, Spectra clustering segmentation of the tumor tissue into 3 dominant regions in the MSI control (**e**) and 21 dominant regions in the TEMI sample (**f**). The MSI experiments were carried out using a 50-µm laser beam raster scan, and the MSI images obtained with mass error tolerance of 10 ppm. Scale bars, 2 mm. Mouse tissue shapes created with BioRender.com.

In addition to cerebellum, we also tested the applicability of TEMI to in situ biomolecular mapping of cancer tissues. Tumor tissue from a murine melanoma model was subjected to a three-round gelation–expansion cycle, achieving ~3.5-fold linear expansion. With a consistent 50-µm laser raster scan, the expanded tumor tissue revealed more detailed features than did the control sample (Fig. 5a–d). The enhanced resolution enabled the spatial separation of metabolic features, such as phospholipids PC (36:1) and PC (38:4), which both had less-distinct spatial patterns without expansion (Fig. 5c,d). The tumor tissue exhibits high heterogeneity, as revealed from spatial distribution of the lipids. On the basis of spectra clustering using bisecting *k*-means, we were able to segment the tumor tissue into 21 dominant regions using the TEMI method; the unexpanded tissue could be segmented into only 3 dominant regions when we set the minimum cluster of 600 spectra (Fig. 5e,f). These results support the ability of TEMI to uncover metabolic heterogeneity in tumor tissues.

### Broad application of TEMI in organ imaging

**Fig. 6:**
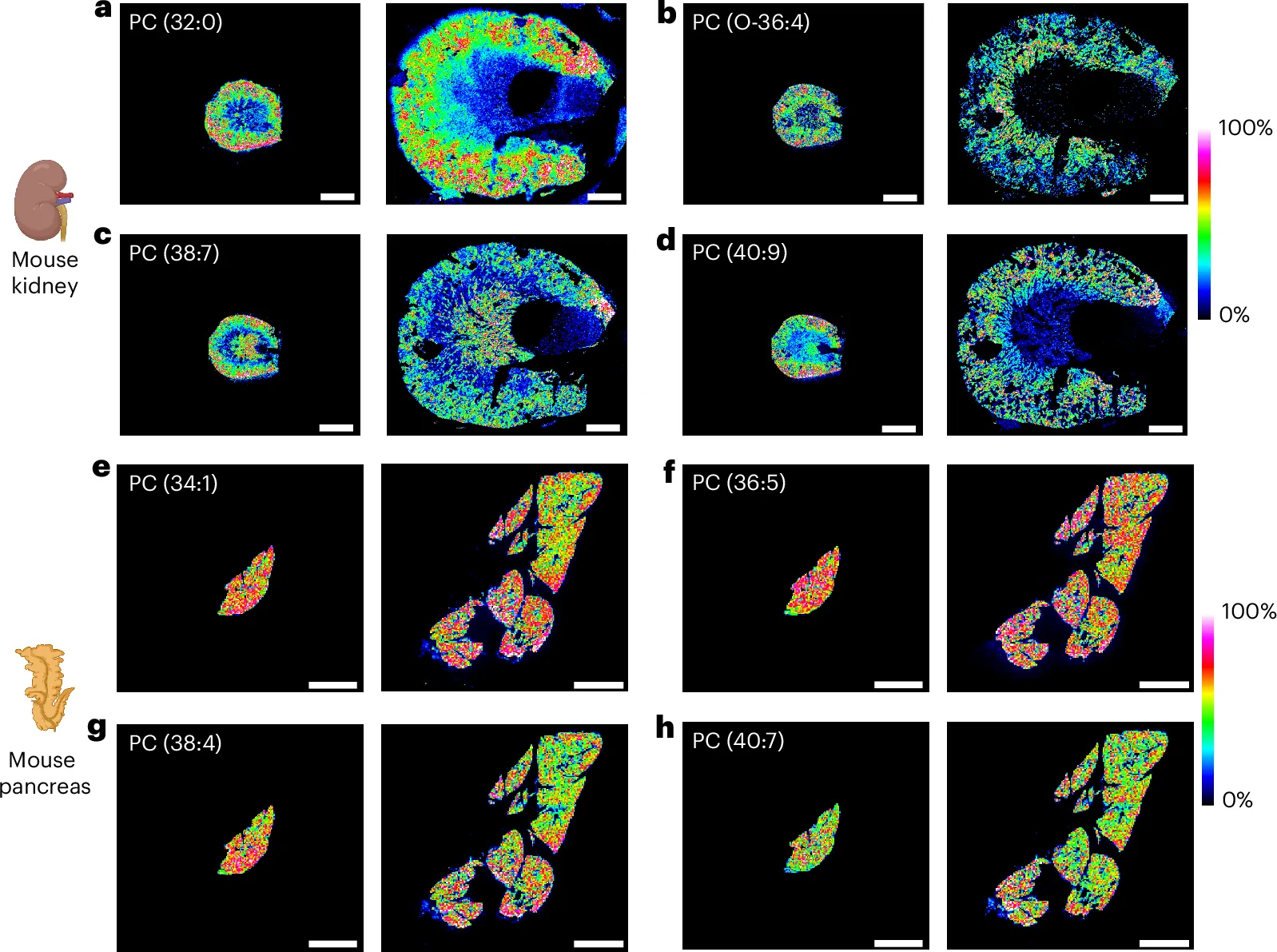
TEMI-based metabolic mapping of kidney and pancreas. **a**–**d**, MS images of representative lipid species detected from unexpanded control (MSI, left) and expanded mouse kidney tissue (TEMI, right), including PC (32:0) ([M+H]^+^, *m/z* 734.568) (**a**), PC (O-36:4) ([M+H]^+^, *m/z* 768.586) (**b**), PC (38:7) ([M+H]^+^, *m/z* 804.549) (**c**), and PC (40:9) ([M+H]^+^, *m/z* 828.550) (**d**). **e**–**h**, MS images of representative lipid species detected from unexpanded control (MSI, left) and expanded mouse pancreas tissue (TEMI, right), including PC (34:1) ([M+H]^+^, *m/z* 760.583) (**e**), PC (36:5) ([M+H]^+^, *m/z* 780.551) (**f**), PC (38:4) ([M+H]^+^, *m/z* 810.600) (**g**) and PC (40:7) ([M+H]^+^, *m/z* 832.581) (**h**). The MSI experiments were carried out using a 50-µm laser beam raster scan, and the MSI images obtained with mass error tolerance of 10 ppm. Scale bars, 2 mm. Mouse tissue shapes created with BioRender.com.

Furthermore, we have applied TEMI for biomolecular mapping of other organs, including mouse kidney and pancreas (Fig. 6), and revealed the spatial distribution of different lipid species in those tissues. Together, these results support broad application of TEMI for spatial molecular mapping. Furthermore, at each stage of gel embedding, we measured the tissue deformations in the cerebellum, kidney, and pancreas, finding that the maximum measurement errors were under 12% (Extended Data Fig. 1 and Supplementary Table 6).

## Discussion

Together, the results of this work demonstrate that the TEMI method can be used for spatially resolved omics in intact tissues at high spatial resolution and chemical sensitivity, thereby revealing in situ biomolecular heterogeneity. In this proof-of-concept study, TEMI was applied not only to lipidomics and metabolomics, but also to the systematic profiling of proteins and N-glycans. Recently, three published and two preprint studies have utilized tissue-expansion protocols with varying harsh denaturing treatments, including high temperature, detergent, proteinase K, or trypsin digestion followed by MALDI-MSI^16–20^. These published methods have overlooked the importance of preserving non-denatured tissue during tissue expansion and the essential step of cryosectioning afterward, which we have found to be important for robust analyte detection. Harsh denaturing treatments can diminish signal strength, making detection sensitivity a limiting factor for method performance and potentially leading to severe biomolecule delocalization upon expansion^16^. By contrast, the new multi-embedding expansion protocol used in the TEMI approach presented here achieves expansion without denaturation, enabling the preservation of biomolecules in expanded tissues and revealing their distribution with demonstrated single-cell resolution. Whereas a previously reported tissue stretching strategy using glass beads embedded in parafilm improves MSI spatial resolution by fragmenting the tissue at the cellular level^21^, TEMI fragments tissue at the molecular scale, and it is compatible with standard MSI processing and correlative light microscopy.

Our tissue-expansion method is capable of yielding a higher expansion factor, and preliminary testing on mouse cerebellum suggests that after seven rounds of re-embedding, a tenfold linear expansion can be achieved (data not shown). Increasing the expansion factor will enhance spatial resolution, especially for single cells or tiny tissues such as organoids or spheroids. However, as expansion increases, the in situ biomolecular density decreases, necessitating further investigation into the optimal thickness for tissue sections and other parameters for improved MSI performance. Furthermore, detection could be limited by the sensitivity of MSI. Based on its design, TEMI can detect small molecules that are non-covalently bound to lipids or proteins. Truly ‘free’ small molecules, especially water-soluble ones, might still diffuse or be partially lost during the expansion step. Developing new anchoring methods to immobilize small molecules will help improve their imaging with TEMI in the future.

In this proof-of-concept study, we used MALDI-MSI for imaging; however, our tissue-expansion protocol is readily compatible with other mass-spectrometry imaging modalities, such as desorption electrospray ionization mass spectrometry imaging and laser-ablation electrospray ionization mass spectrometry imaging. TEMI is compatible with tissue volume imaging by arraying cryoslices, but the *z*-dimension resolution is limited by the section thickness. Of note, covalent chemical anchoring of the tissue could impact detection sensitivity of biomolecules containing primary amines, owing to reactions with primary amine moieties during tissue gelation. As mentioned in the method section, when it comes to detecting peptides or proteins, we suggest avoiding staining with NHS ester dyes, as they react with the primary amines of lysine and amino termini of proteins, which could consequently affect peptide detection. To advance multiplex IHC carried out after expansion with TEMI, further optimization is required, because chemical anchoring of the target protein antigens might affect antibody binding efficiency. With further development and optimization of sampling protocols, we envision that TEMI will offer a promising, crucial platform to uncover the spatial distribution and chemical specificity of various biomolecules and reveal biomolecular heterogeneity across multiple scales in diverse biomedical samples.

## Methods

### Chemicals and materials

Reagents including Optima LC–MS-grade methanol, ethanol, acetonitrile, 2-propanol (IPA), water, and formic acid were purchased from Fisher Scientific; 6-((acryloyl)amino) hexanoic acid succinimidyl ester (AcX), sodium acrylate, acrylamide, *N*,*N*′-methylenebis acrylamide (Bis), *N*,*N*,*N*′,*N*′-tetramethyl ethylenediamine (TEMED), ammonium persulfate (APS), 4-hydroxy TEMPO, 1,5-diaminonaphthalene (DAN), 2,5-dihydroxybenzoic acid (DHB), *N*-(1-naphthyl)ethylenediamine dihydrochloride (NEDC), 4′,6-diamidino-2-phenylindole (DAPI), ethylenediaminetetraacetic acid (EDTA), triphenylphosphine (TPP), butylated hydroxytoluene (BHT), *tert*-butyl methyl ether (MTBE) (≥99.5%), and ammonium formate (Crystals, ≥99.0%, Eluent additive for LC–MS) were purchased from Sigma-Aldrich. SPLASH Lipidomix Mass Spec Standard was purchased from Avanti Polar Lipids. Metabolomics QReSS Standard Mix 1 was purchased from Cambridge Isotope Laboratories. D1032-30 prefilled 2.0-ml tubes with 3.0-mm-diameter triple-pure high-impact zirconium beads were purchased from Benchmark Scientific. Indium tin oxide (ITO)-coated glass microscope slides (25 mm × 75 mm × 1.1 mm) were purchased from Delta Technologies.

Animal experiments received approval from the Animal Care and Use Committee at the University of Wisconsin–Madison. Mouse brain samples were collected from female wild-type C57BL/6 mice aged 6–8 months. Mice were housed in facilities with a standard light–dark cycle and humidity of 50% at 24 °C. The mice were housed and treated under a protocol approved by the Institutional Animal Care and Use Committee at the University of Wisconsin–Madison or Howard Hughes Medical Institute (Janelia Research Campus).

For collection of fixed mouse brain tissues, animals were transcardially perfused with 4% formaldehyde in 1× PBS, followed by postfixation of the brain in the same solution overnight at 4 °C. Fixed tissue was washed in 1× PBS and sectioned using a vibratome. For collection of fresh mouse brain tissues, mice were euthanized, followed by decapitation. Whole brain tissues were surgically collected and snap frozen in liquid nitrogen immediately. The mouse brain tissue samples were stored at −80 °C until use.

Mouse tumor samples were collected from a murine melanoma model. For tumor experiments, female wild-type C57BL/6 mice aged 10–14 weeks were purchased from Taconic Biosciences. The murine melanoma B78-D14 (B78) cell line, supplied by ATCC, was cultured at 37 °C with 5% CO_2_ in RPMI-1640 medium (Gibco) containing 10% fetal bovine serum (FBS) (Gibco) and 1% penicillin–streptomycin solution (Gibco). B78 tumors were engrafted by subcutaneous flank injection of 4 × 10^6^ murine melanoma B78-D14 (B78) cells. Tumor size was determined using calipers, and volume was approximated using the formula (width^2^ × length) / 2. Tumor samples were collected when they reached a size of ∼600 mm^3^, which was done approximately 5 weeks following engraftment.

Fresh mouse brain and tumor samples were fixed in 4% formaldehyde 1× PBS for 4 h at room temperature, followed by washing with 1× PBS three times. Then, the tissue blocks were embedded in 6% (wt/wt) agarose and sectioned into tissue slices at 500-µm thickness using a vibratome (Leica VT1000 S).

### Sample preparation for TEMI

The schematic workflow of sample preparation for TEMI is presented in Fig. 1. Tissue expansion was initially based on prior implementations of expansion microscopy^22^; however, adjustments were made to tailor the expanded samples specifically for MALDI-MS imaging purposes. Mice were perfused with formaldehyde as described above, organs were dissected, and the adjacent tissue slices were cut into 500 µm and 300 µm for unexpanded control and for TEMI sample preparation, respectively.

To anchor proteins before gelation, 300-µm tissue slices were treated in a solution of 0.2 mg ml^–1^ AcX in 1× PBS for 1 h at room temperature, followed by washing with 1× PBS. Stock solutions of monomer solution components were prepared and stored frozen as in our previous study^14^: 40% acrylamide, 4 M sodium acrylate, 1% (wt/vol) bisacrylamide, 0.5% (wt/vol) 4-hydroxy TEMPO (4HT), 10% (vol/vol) TEMED, and 10% (wt/vol) APS in deionized water. The TREx1000 gelation solution was prepared according to the recipe from our previous study^14^, with final concentrations of 1 M sodium acrylate, 14% acrylamide, 0.1% bisacrylamide, 1× PBS, 0.01% 4HT, 0.2% TEMED, and 0.2% APS. All components, except for 4HT, TEMED, and APS, were mixed to produce monomer solution, aliquoted, and stored frozen. 4HT, TEMED, and APS (gelation inhibitor, accelerator, and initiator) stocks were thawed and added to monomer solution on ice to produce complete gelation solution within minutes of use. Tissue slices were incubated in complete gelation solution on ice for 30 min with shaking before being mounted in gelation chambers and gelled in a humidified 37 °C incubator for 2 h as previously described^14^.

After gelation, the gelation chamber was carefully disassembled, and the gelled tissue was trimmed out using a scalpel. Then, the gelled tissue was collected and incubated in 1× PBS for 30 min at 4 °C and re-embedded in the same gelation solution for each additional round of gelation. After the desired number of rounds of embedding and expansion, both unexpanded and expanded tissues were optionally stained with 0.5 μg ml^–1^ DAPI and/or 1 μg ml^–1^ Alexa Fluor 488 NHS ester dye in 1× PBS overnight or for 1 h; following staining, tissues were washed with 1× PBS three times for 30 min for fluorescence imaging of DNA and/or proteins, respectively. Depending on the specific application in MSI, staining choices should be made accordingly: if one is interested in detecting DNA, DAPI staining should be skipped; if one is interested in detecting peptides or proteins, Alexa Fluor 488 NHS ester dye staining should be skipped because it occupies the primary amines of lysine and N termini of proteins.

Both unexpanded and expanded tissues were incubated in 30% (wt/wt) sucrose cryoprotectant in 1× PBS for 4 h (the sucrose solution was replaced with new solution every hour), to protect against the formation of large ice crystals during cryosectioning. Then, the gel was embedded in 10% (wt/wt) gelatin after extra gel was trimmed outside the tissue, gently pressed flat in case of mild curvature, and snap-frozen on dry ice before cryosectioning at 30 µm in a cryostat (Thermo Fisher Scientific) at −30 °C. Tissue sections were mounted on ITO slides coated in poly-l-lysine (Electron Microscopy Sciences) and dried in a desiccator for 20 min, and then stored in −80 °C until MSI processing.

The tissue slides were thawed and dried again in a desiccator for 30 min before the application of the MALDI matrix. For dual-polarity MS imaging of lipid species, DAN matrix (10 mg ml^–1^) in acetonitrile/water (vol/vol, 80:20) solution was sprayed onto the tissue samples at a flow rate of 75 μl min^–1^ with tracking space of 3 mm for 6 passes using a M5-Sprayer (HTX Technologies). The moving velocity of the nozzle was set to 1,000 mm min^–1^, the nozzle temperature was set to 75 °C, and the nozzle nitrogen gas pressure was 10 psi. A drying time of 30 s was applied between each pass. For small metabolites, *N*-glycan, and tryptic peptide analyses in positive ionization mode, DHB (40 mg ml^–1^ with 0.1% formic acid in acetonitrile/water (vol/vol, 80:20) solution) was used under the same parameters setting of the M5-Sprayer, whereas for small metabolite analysis in negative mode, NEDC matrix (10 mg ml^–1^ with 0.2% ammonium hydroxide in acetonitrile/water (vol/vol, 80:20) solution) was used with the same M5-Sprayer settings.

For N-glycan and tryptic peptide MS imaging on the same tissue slides after lipid or metabolite analysis, the remaining MALDI matrix on the tissue was removed by washing the slides three times in 100% ethanol, 95% ethanol, and 70% ethanol for 5 min per wash. Then, the tissue slides were dried in a fume hood at room temperature, followed by immersion in antigen-retrieval buffer containing 100 mM acidic Tris and 1 M hydroxylamine hydrochloride for 2 h at 60 °C. A 10% (vol/vol) TFA solution was used to adjust the pH of the antigen retrieval buffer to 3. After the decrosslinking-inducing antigen-retrieval treatment, the slides were washed three times with 95% and 70% ethanol solutions for 2 min each.

For in situ releasing of N-glycans, 20 μl of PNGase F (20 U, Promega) was dissolved in 380 μl of 20 mM ammonium bicarbonate (ABC) buffer, which was sprayed onto tissue sections using the TM-Sprayer with the following setup: flow rate of 20 μl min^–1^, nitrogen gas pressure at 8 psi, nozzle temperature at 35 °C, nozzle velocity of 800 mm min^–1^ and tracking space of 3 mm. Following incubation in a humid chamber at 37 °C for 8 h, sections were sprayed with DHB (40 mg ml^–1^ with 0.1% formic acid in acetonitrile/water (vol/vol, 80:20)) using the TM-Sprayer with the aforementioned DHB matrix spraying settings.

For label-free protein MS imaging, in situ tryptic digestion was performed. In brief, tissue sections were washed with 70% ethanol to remove the remaining DHB matrix and released N-glycans, placed in a fume hood until dry, then sprayed with freshly prepared 50 ng µl trypsin (Promega) in 20 mM ABC buffer using the same TM-Sprayer parameters described above for PNGase F deposition. After incubation in a humid chamber at 37 °C for 8 h, sections were sprayed with DHB (40 mg ml^–1^ with 0.1% formic acid in acetonitrile/water (vol/vol, 80:20)) using TM-Sprayer with the same TM-Sprayer parameters described above for matrix application.

For antibody-based targeted protein MSI, photocleavable mass-tag (PC-MT) MALDI-immunohistochemistry (IHC) was performed. PC-MT tagged antibody (AmberGen) staining was based on AmberGen’s protocol^23^. The working antibody probes were diluted in tissue blocking buffer (2% (vol/vol) normal rabbit serum, 2% (vol/vol) normal mouse serum, and 5% (wt/vol) BSA prepared in TBS (50 mM Tris-HCl, pH 7.5, 200 mM NaCl) with 0.05% (wt/vol) octyl β-d-glucopyranoside) to a final concentration of 2.5 μg ml^–1^. The PC-MT probes on the stained tissue sections were photocleaved by illumination with 365-nm ultraviolet (UV) light using a Phrozen UV curing lamp for 10 min (3 mW cm^2^) to achieve maximum photocleavage before applying α-cyano-4-hydroxycinnamic acid matrix (10 mg ml^–1^ with 0.1% formic acid in acetonitrile/water (vol/vol, 80:20) solution) on the samples. The TM-Sprayer was configured with the following settings: flow rate of 100 μl min^–1^ for 6 passes, drying time of 30 s between each pass, nozzle nitrogen gas pressure at 10 psi, nozzle temperature at 75 °C, nozzle velocity of 1,000 mm min^–1^, and tracking space of 3 mm.

### Extraction of mouse brain lipidome for liquid chromatography–mass spectrometry analysis

Cerebellar slices were cut with a thickness of 300 µm. Three groups, control (without expansion), Ex (expanded without applying proteinase K), and ExP (expanded with applying proteinase K) were used for lipidomics analysis. The wet mass of sample slices was around 5 mg each for unexpanded controls, or 70 mg after expansion. All the slices were washed in ddH_2_O twice for 1.5 h each in a bead-beater tube. After all the water was removed, 200 µl of LC–MS water with 5 µl antioxidant stock solution (0.2 mg ml^–1^ TPP, 0.2 mg ml^–1^ BHT, and 1 mg ml^–1^ EDTA) was added. Then, the sample was homogenized with 3.0-mm zirconium beads at maximum speed for 3 min (6 cycles of 30-s intervals). Then, all the homogenized solutions were transferred into a 4-ml wide-mouth glass vial, and 5 µl of SPLASH LIPIDOMIX Mass Spec Standard was added. Next, the lipids were extracted by adding methanol (LC–MS grade) and vortexed for 1 min, followed by the addition of MTBE and shaking for 1 h at 25 °C. Phase separation was induced by adding LC–MS-grade water, with a final mixing ratio of MTBE/methanol/water of 10:3:2.5 (vol/vol/vol)^24^. Because the expanded sample was swollen with water by the hydrogel, there was no extra water needed for these specimens. The upper (organic) phase was collected and dried under a stream of nitrogen gas. The lipid extracts were stored at −80 °C before analysis.

### Extraction of polar metabolome from mouse cerebellum

The polar metabolites were extracted from the mouse cerebellum using dry-ice-cold 80% methanol. The sample, mixed with 2 µl of Metabolomics QReSS Standard Mix 1, following the manufacturer’s instructions, was homogenized with 3.0-mm zirconium beads at the highest speed for six cycles of 30 s, with a 5-min cool down in between the rounds.

### Extraction of tryptic peptides for liquid chromatography–tandem mass spectrometry analysis

The tryptic peptides on the tissue sections were extracted with 10 µl of 50% acidic acetonitrile (0.1% formic acid (FA)) and 80% acidic acetonitrile (0.1% FA) five times. In brief, an aliquot of 10 µl solvent was deposited onto the tissue section using a pipette; after 3-s extraction, the solvent was retracted back into the pipette and transferred into a 0.6-ml vial. All peptide extraction solutions from three tissue section replicates were combined and dried in a SpeedVac. The samples were reconstituted in 100 µl 0.1% FA in water, and desalting was performed using OMIX C18 Pipette Tips (Agilent). The process began with wetting of the tip with three aspirating cycles of acetonitrile. Next, the tip was equilibrated by aspirating a 0.1% formic acid water three times. Following this, a 90 µl sample volume was aspirated into the pipette tip and cycled at least ten times for thorough enrichment. The tip was then washed three times with the 0.1% formic acid water solution. Two clean microcentrifuge tubes were prepared, each containing 100 µl of 50% and 80% acetonitrile water mixture (vol/vol) with 0.1% formic acid. The solutions were used to aspirate and dispense eluent through the C18 pipette tip, with cycling at least 10 times to ensure effective elution of the desalted peptides. Finally, the two eluents were combined and dried in vacuo.

### Data acquisition and analysis

All MALDI-MSI experiments were conducted using a TimsTOF fleX mass spectrometer (Bruker Scientific), with timsContol and flexImaging for data acquisition. The system was coupled with a SmartBeam 3D 10-kHz-frequency tripled Nd:YAG laser (355 nm). The laser settings used were 10–50 μm diameter at smart mode, 100 shots per pixel, and a raster step size corresponding to the laser spot size during MS imaging. The laser power was set to 40–80%, with 100–250 laser shots. For lipid analysis, MS imaging data were collected under positive ionization mode over a mass range of 400–1,000 Da; for the subsequent negative imaging run on the same tissue sample, 100 shots with a laser energy of 45% were conducted. It is important to note that, in dual ionization of the same tissue section, it is crucial to carefully control the laser energy at an appropriate level and refrain from using excessive laser energy during the first MSI run, as excessive laser energy could damage the tissue and ultimately compromise the performance in the subsequent MSI run. A varied mass range of 100–3,000 Da was used for imaging small metabolites, tryptic peptides, and N-glycans. The instrument offers a mass resolving power of approximately 40,000 (full width at half-maximum) in the lipid mass range, resulting in high mass accuracy in MS imaging experiments. Other instrumental parameters include collision RF of 800–4000 volts peak-to-peak (Vpp), ion transfer time of 20–80 μs, prepulse storage time of 5–10 μs, and multipole RF of 350 Vpp. In MALDI–tandem mass spectrometry (MS/MS) experiments, target precursor lipid ions were isolated at a mass window of 1.5 Da for higher-energy collisional dissociation (HCD) fragmentation with a collision voltage of 30–60 eV. MS data were analyzed with Compass Data Analysis (Bruker), and the MSI images were visualized using SCiLS Lab Pro (Bruker) with data normalized to total ion count (TIC) and no denoising processed. Unsupervised statistical analysis tool of Bisecting *k*-means was used for an arbitrarily segmentation of all single spectrum from the sample based on the detected peaks of lipids; each segment is annotated with a different color. Lipids, small metabolites, and tryptic peptides detected from the tissue samples were identified on the basis of the combination of in situ MALDI–MS/MS of target analytes, as well as referring to high-performance liquid chromatography (HPLC)–nanoESI–MS/MS results of metabolite, lipid, and tryptic peptides extracts from the tissue samples. The compositions of N-glycans observed were tentatively assigned through searches in the GlyGen (http://www.glygen.org) and GlycoWorkbench (https://code.google.com/archive/p/glycoworkbench/) with a mass tolerance of 10 ppm.

For conventional lipidomic analysis, the lipid extracts were re-dissolved in 55 µl of IPA/methanol (1:1), and 12 µl was injected to run LC–MS. The experiments were performed on an Orbitrap Fusion Lumos Tribrid Mass Spectrometer equipped with Ion Max API source housing with HESI-II probe and Vanquish UHPLC System (Thermo Fisher Scientific). The analytes were separated on a Accucore Vanquish C18 + UHPLC Column (2.1 × 150 mm, 1.5 µm) column operated at 55 °C and a flow rate of 150 µl min^–1^ using a 52-min gradient. The gradient is shown in Supplementary Table 7. Mobile phase A consisted of 60:40 (vol/vol) acetonitrile/water with 10 mM ammonium formate and 0.1% formic acid; mobile phase B consisted of 90:10 (vol/vol) IPA/acetonitrile with 10 mM ammonium formate and 0.1% formic acid. MS spectra were acquired with Xcalibur software (Thermo Fisher Scientific). We conducted a comprehensive data-dependent HCD MS2 experiment with conditional CID MS2 and MS3 data acquisition. Alternating positive- and negative-ion data-dependent HCD MS2 experiments were performed using two 1.0-s cycles (retention time range, 0–31.2 min) with stepped collision energy (24%, 27%, 30%). Additional CID MS2 scans were triggered on the same precursor ion for PC lipids with a diagnostic fragment ion (*m/z* 184.0733) detected from the positive HCD MS2 data. For the retention time range of 31.2–52 min, a positive-ion, data-dependent MS2 experiment was performed using a 2-s cycle time. Additional CID MS3 scans were subsequently triggered on the three largest HCD product ions that had lost neutral fatty acid plus ammonia.

The raw files were analyzed by LipidSearch5 (Thermo Fisher Scientific). The lipids were identified by matching product ion spectra to the LipidSearch library (Supplementary Data 1). Both precursor and product ion mass tolerance were set at 5 ppm and aligned on the basis of retention time tolerance of 0.05 min. After manually removing the duplicates and checking the peak and product ions, as well as selecting the main ion for each lipid, the final dataset with 659 lipids was used for further analysis. Positive ions were normalized to the internal reference PE (18:1_15:0) (d7) and negative ions to the internal reference of PC (15:0_18:1) (d7) in each sample. After normalization, all data were grouped and filtered on the basis of at least three valid values in at least one group. The data were log_2_-transformed, and the missing values were imputed on the basis of a normal distribution. The analysis of variance (ANOVA) test was performed for significance analysis, followed by post hoc Tukey’s HSD test (Supplementary Table 8). A Benjamini–Hochberg adjusted *P* value of less than or equal to 0.05 was considered significant using Perseus^25,26^.

For LC–MS/MS analysis of polar metabolites, an Orbitrap Fusion Lumos Tribrid Mass Spectrometer equipped with Ion Max API source housing with HESI-II probe and the Vanquish UHPLC System (Thermo Fisher Scientific) was used. Four microliters of the resuspended sample in methanol were injected onto a Waters Acquity UPLC BEH Amide column (150 mm × 2.1 mm, 1.7 µm) operated at 45 °C with a flow rate of 200 µl min^–1^ using a 68-min gradient. The gradient is detailed in Supplementary Table 9. Mobile phase A consisted of water with 10 mM ammonium formate and 0.125% formic acid; mobile phase B consisted of acetonitrile/water (95/5, col/col) with 10 mM ammonium formate and 0.125% formic acid. The ion source conditions were set as follows: spray voltage of 3.5 kV for positive mode, 2.5 kV for negative mode; sheath gas at 50 arbitrary units; aux gas at 10 arbitrary units; sweep gas at 1 arbitrary unit; ion transfer tube temperature at 325 °C; and vaporizer temperature at 350 °C. The following acquisition parameters were used for MS1 analysis: resolution 120,000; AGC target, standard; maximum injection time (IT), auto; scan range, 60–700 *m/z*; data type, profile. Data-dependent MS/MS parameters were: resolution, 30,000; AGC target, standard; maximum IT, auto; isolation window, 0.7 *m/z*; activation type, HCD; a stepped collision energy mode of 20, 30, and 40; data type, centroid. The raw files are analyzed using Compound Discoverer (Thermo Fisher Scientific) for untargeted metabolomics.

For LC–MS/MS analysis of extracted tryptic peptides, an Orbitrap Ascend Tribrid Mass Spectrometer equipped with the Vanquish Neo UHPLC System (Thermo Fisher Scientific) was used. Five microliters of the sample were injected onto an Aurora Ultimate XT 25×75 C18 UHPLC column (25 cm × 75 µm, 1.7 µm) operated at 35 °C and a flow rate of 200 nl min^–1^ using a 130 min gradient. The gradient is detailed in Supplementary Table 10. Mobile phase A consisted of water with 0.1% formic acid; mobile phase B consisted of 80% acetonitrile with 0.1% formic acid. The ion source conditions were as follows: spray voltage, 1.9 kV in positive mode; ion transfer tube temperature, 275 °C. The following acquisition parameters were used for MS1 analysis: resolution, 120,000; normalized AGC target, 250%; maximum IT, 50 ms; scan range, 300–2,000 *m/z*; data type, profile. Data-dependent MS/MS parameters were: resolution, 15,000; AGC target, standard; maximum IT, auto; isolation window, 1.1 *m/z*; activation type, HCD; collision energy mode, normalized, 25%; data type, centroid.

The raw files were analyzed using FragPipe-based MSFragger. The tryptic peptides were searched against a database containing the reviewed proteome for the *Mus musculus* database from Uniprot with the false-discovery rate for precursors set at 1%. The precursor mass tolerance was set to 10 ppm, and a maximum of four missed cleavages was allowed. Dynamic modifications including deamidation of asparagine and glutamine, oxidation on methionine and cysteine, and +12 Da, +14.017 Da, or +30.011 Da on any amino acids was allowed.

### Expansion deformation mapping

To measure non-uniformity of expansion, we stained the tissue with 0.5 μg ml^–1^ Alexa Fluor 488 NHS ester dye after the anchoring step, and tissues were imaged at each stage of gel embedding. We analyzed the images using BigWarp (Fiji) and MATLAB, as described previously^27,28^. In brief, the expanded tissue image was registered to the pre-expansion tissue image using manually picked landmarks. The deformation field produced from these landmarks was used to calculate the expansion factor and measure the degree of deformation due to expansion non-uniformity (Supplementary Note 2).

### Reporting summary

Further information on research design is available in the Nature Portfolio Reporting Summary linked to this article.

## Online content

Any methods, additional references, Nature Portfolio reporting summaries, source data, extended data, supplementary information, acknowledgements, peer review information; details of author contributions and competing interests; and statements of data and code availability are available at https://doi.org/10.1038/s41592-025-02664-9.

## Supplementary information

Supplementary Information Supplementary Figures 1–18, Supplementary Tables 1–10, and Supplementary Notes 1–3.

Reporting Summary

Supplementary Data 1 Results of lipidomics, metabolomics, and proteomics.

## Source data

Source Data Extended Data Fig. 1 Deformation maps and quantification of measurement errors between the 1st, or 2nd, or 3rd embedding and pre-expansion of different mouse tissue types.

## Data Availability

The MS imaging data included in this Article are available from the corresponding author upon request. All the LC–MS raw files have been deposited into MassIVE (dataset identifier MSV000096466). All the Source Data for Figs. 1–6 and Extended Data Figs. 3 and 4 of MALDI-MSI raw files have been deposited into MassIVE (dataset identifier MSV000097036). Source data are provided with this paper.

## Code availability

The code of data for the quantification of tissue expansion non-uniformity is available in Supporting Information materials as Supplementary Note 3.
