# Supplementary material for: TEMI: tissue-expansion mass-spectrometry imaging
Source: Nat Methods. 2025 Apr 22;22(5):1051–8. doi: 10.1038/s41592-025-02664-9 (PMC12074994; doi:10.1038/s41592-025-02664-9)
Supplement: Supplementary file 1 — Supplementary Figures 1–18, Supplementary Tables 1–10, and Supplementary Notes 1–3. [file 41592_2025_2664_MOESM1_ESM.pdf]

nature methods

Supplementary information https://doi.org/10.1038/s41592-025-02664-9

TEMI: tissue-expansion mass-spectrometry imaging

In the format provided by the authors and unedited

Supplementary Information

TEMI: Tissue Expansion Mass Spectrometry Imaging

Hua Zhang[1#], Lang Ding[2,3#], Amy Hu[2], Xudong Shi[4], Penghsuan Huang[5], Haiyan Lu[1], Paul W. Tillberg[2*], Meng C. Wang[2*], and Lingjun Li[1, 5*]

[1]School of Pharmacy, University of Wisconsin-Madison, Madison, Wisconsin 53705, USA

[2]Janelia Research Campus, Howard Hughes Medical Institute, Ashburn, VA 20147, USA

[3]Graduate Program in Chemical, Physical & Structural Biology, Graduate School of Biomedical Science, Baylor College of Medicine, Houston, TX 77030, USA

[4]Division of Otolaryngology, Department of Surgery, School of Medicine and Public Health, University of Wisconsin-Madison, Madison, Wisconsin 53792, USA

[5]Department of Chemistry, University of Wisconsin-Madison, Madison, Wisconsin 53705, USA

# These authors contributed equally to this work.

* Corresponding authors:

Paul W. Tillberg: tillbergp@janelia.hhmi.org

Meng C. Wang: mengwang@janelia.hhmi.org

Lingjun Li: lingjun.li@wisc.edu

Table of Content

Supplementary Figure 1. Cryosectioning of mouse cerebellum after tissue expansion treatment.

Supplementary Figure 2. MALDI-MS spectra of expanded mouse brain tissue sections.

Supplementary Figure 3. Lipidomics results.

Supplementary Figure 4. Representative MS spectra of LC-MS lipidomics.

Supplementary Figure 5. Method comparison of methodologies including TEMI, GAMSI, and Ex-MSI.

Supplementary Figure 6. TEMI of a half mouse brain with a single round gel embedding.

Supplementary Figure 7. Representative mass spectra from surrounding gelatin region and ~3.5-fold expanded tissue region.

Supplementary Figure 8. Single spectra of TEMI sample for chemical delocalization evaluation.

Supplementary Figure 9. MSI of ~2.5-fold linear expanded mouse cerebellum tissue under positive mode.

Supplementary Figure 10. MSI of ~2.5-fold linear expanded mouse cerebellum tissue under negative mode.

Supplementary Figure 11. TEMI images of a ~2.5 linearly expanded mouse cerebellum tissue.

Supplementary Figure 12. TEMI results from mouse cerebellum tissue with 3 times gel-embedding and expansion treatment.

Supplementary Figure 13. Representative MS images of the other unknown small molecules in positive mode.

Supplementary Figure 14. Representative MS images of the other unknown small molecules in negative mode.

Supplementary Figure 15. MS2 matching between fragmentation scan of our study and the matched reference scan from the mzCloud database.

Supplementary Figure 16. Representative MS images of on-tissue digested peptides expanded cerebellum.

Supplementary Figure 17. Representative MS images of unexpanded and TEMI samples by IHC-MALDI-MSI.

Supplementary Figure 18. Comparative spatial mapping of N-glycans from the mouse cerebellum between unexpanded control and TEMI.

Supplementary Table 1. List of the identified lipid species from mouse brain samples using TEMI under positive mode.

Supplementary Table 2. List of the identified lipid species from mouse brain samples using TEMI under negative mode.

Supplementary Table 3. Details of detected lipid species in Purkinje neuron cells using TEMI under positive mode.

Supplementary Table 4. List of the identified on-tissue tryptic peptides matched with LC-MS proteomics results from mouse cerebella using TEMI under positive mode.

Supplementary Table 5. N-glycans detected from mouse cerebellum using TEMI.

Supplementary Table 6. The expansion factor and the maximum measurement error of different tissue types under 1st, 2nd, and 3rd embeddings.

Supplementary Table 7. The LC-MS gradient for the lipidomic analysis.

Supplementary Table 8. The significantly changed lipids from the lipidomic analysis.

Supplementary Table 9. The LC-MS gradient for the metabolomics analysis.

Supplementary Table 10. The LC-MS gradient used for the proteomics analysis.

Supplementary Note 1. Measurement of expansion non-uniformity.

Supplementary Note 2. Comparison among TEMI, GAMSI, Ex-MSI.

Supplementary Note 3. Codes for Figure S7 and expansion factor.

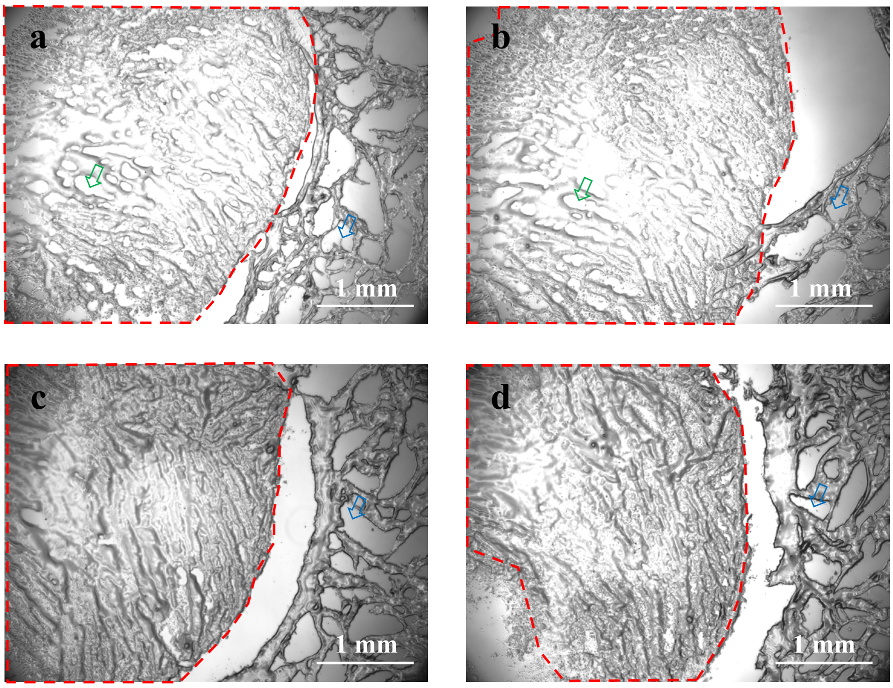

Supplementary Figure 1. Cryosectioning of mouse cerebellum after tissue expansion treatment: bright-field microscope images of expanded tissue sections cryosectioned with different thickness a. 10 μm. b. 12 μm. c. 20 μm. d. 30 μm. The dashed red circle represents the expanded tissue regions; the green hollow arrows on the tissue indicate where tears form due to shrinkage of the tissue upon drying; the blue arrows denote similar tears in the hydrogel adjacent to the tissue.

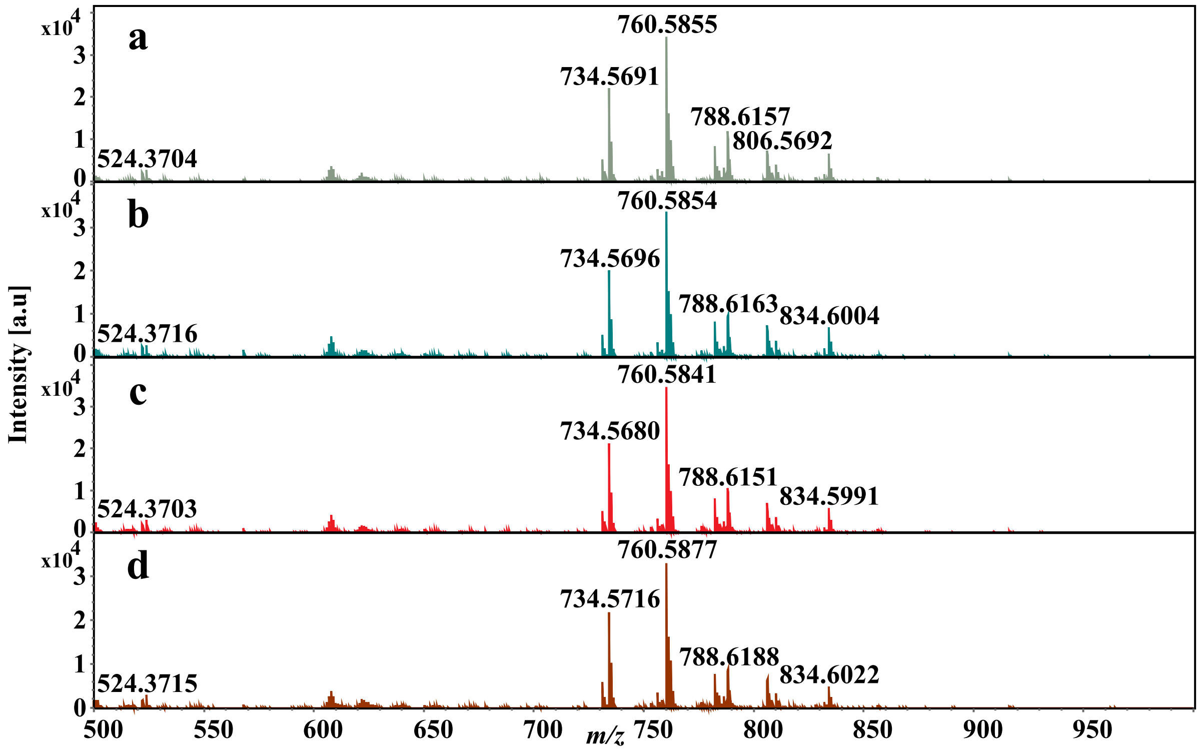

Supplementary Figure 2. MALDI-MS spotting mass spectra of expanded mouse brain tissue sections with varied sectioning thickness. a. 10 μm. b. 12 μm. c. 20 μm. d. 30 μm. MALDI-MS spotting of these tissue sections indicated that the signal intensities exhibit no significant decline as the sectioning thickness increases from 10 to 30 μm.

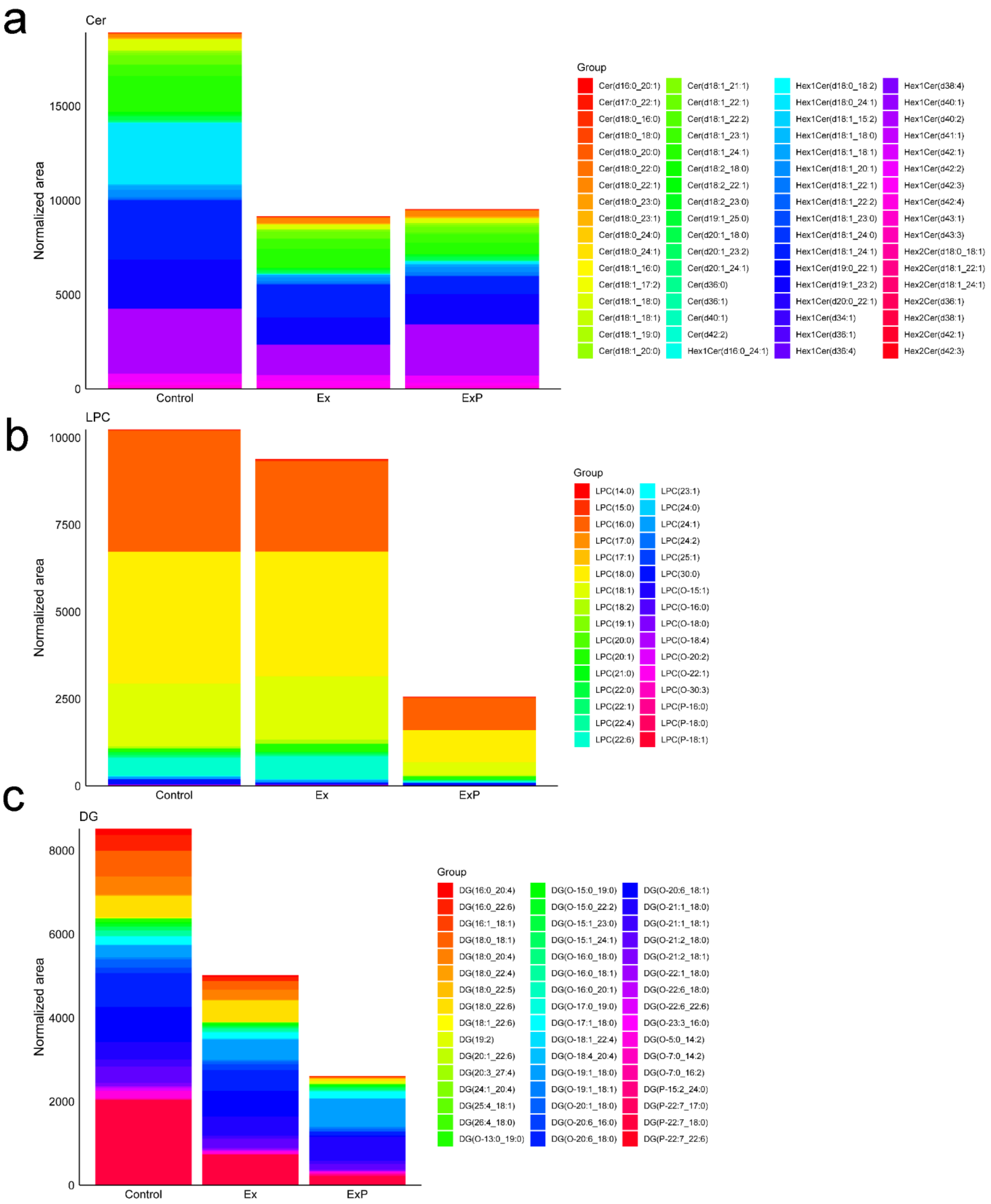

Supplementary Figure 3. Lipidomics results. Three groups: control (without expansion), Ex (expanded without applying ProK), ExP (expanded with applying ProK). a. Ceramide (Cer)-type lipids. b. Lysophosphatidylcholine (LPC)-type lipids. c. Diacylglycerol (DG)-type lipids.

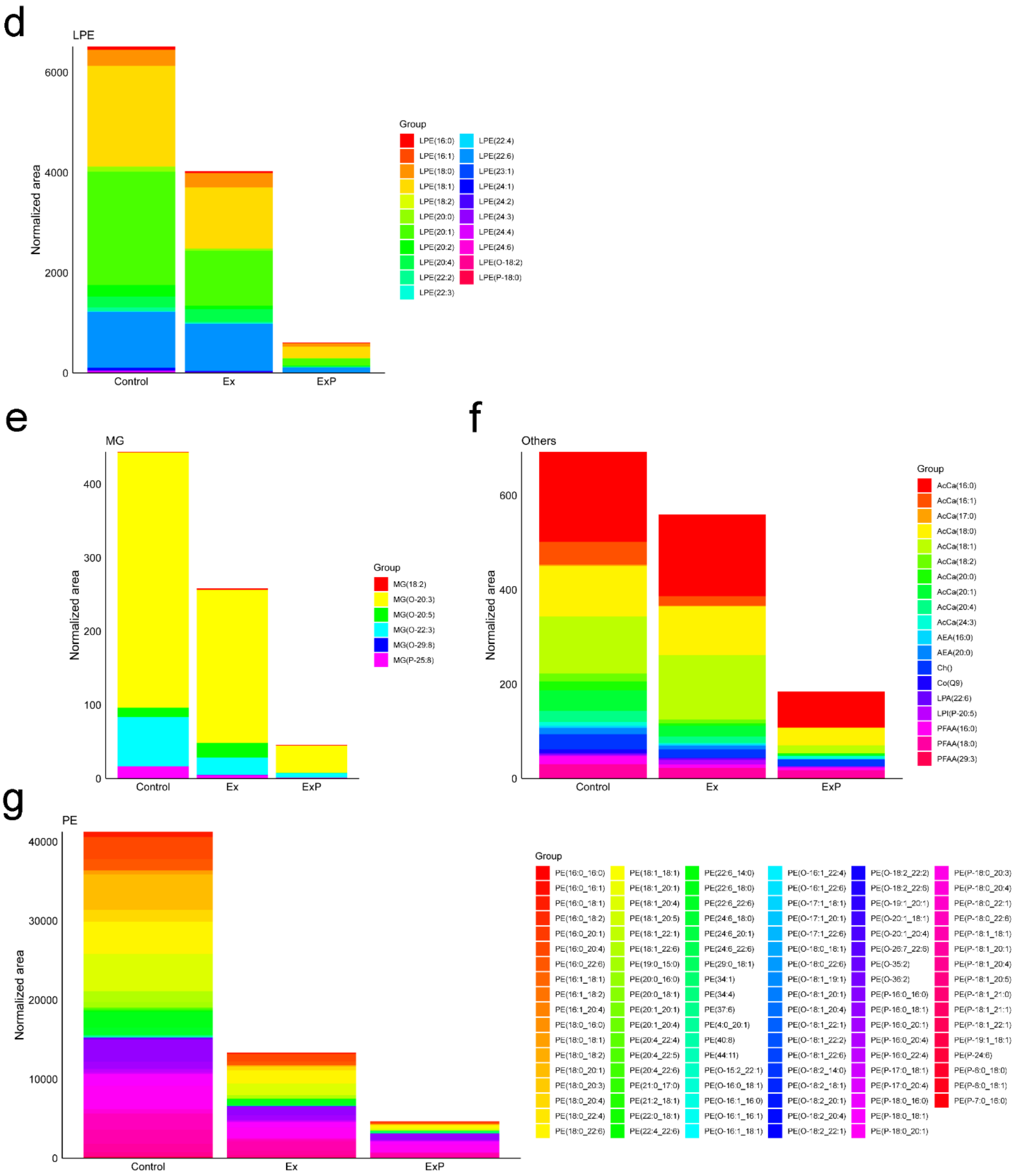

Supplementary Figure 3 (continued). Lipidomics results. d. Lysophosphatidylethanolamine (LPE)-type lipids. e. Monoradylglycerolipid (MG)-type lipids. f. The other types of lipids: Fatty acyl carnitines (AcCa), Cholesterol (Ch), Coenzyme Q (Co), Lysophosphatidic Acid (LPA), Lysophosphatidylinositol (LPI), Primary amides (PFAA). g. Phosphatidylethanolamine (PE)-type lipids.

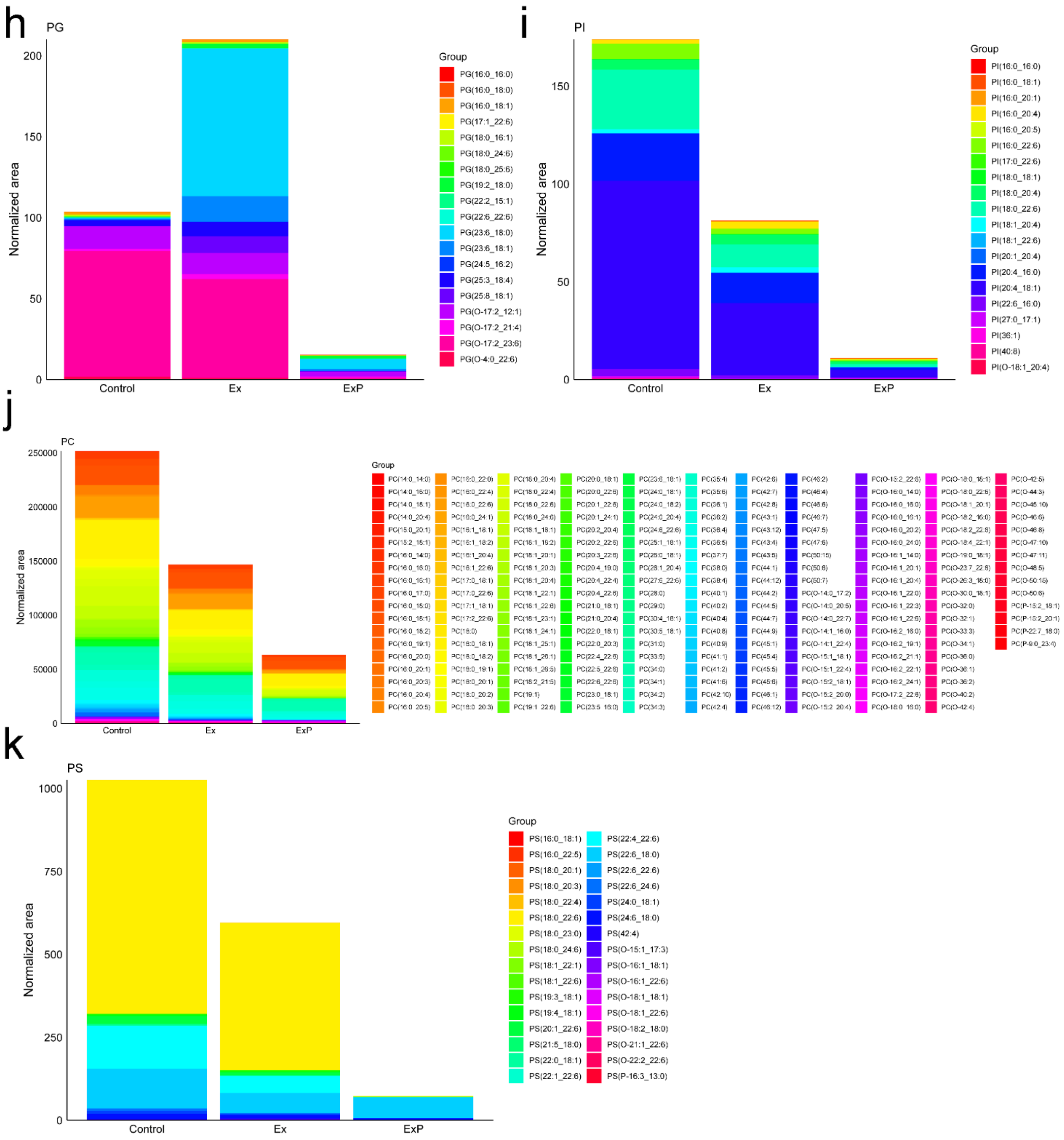

Supplementary Figure 3 (continued). Lipidomics results. h. Diacylglycerophosphoglycerol (PG)-type lipids. i. Phosphatidylinositol (PI)-type lipids. j. Phosphatidylcholine (PC)-type lipids. k. Phosphatidylserine (PS)-type lipids.

l

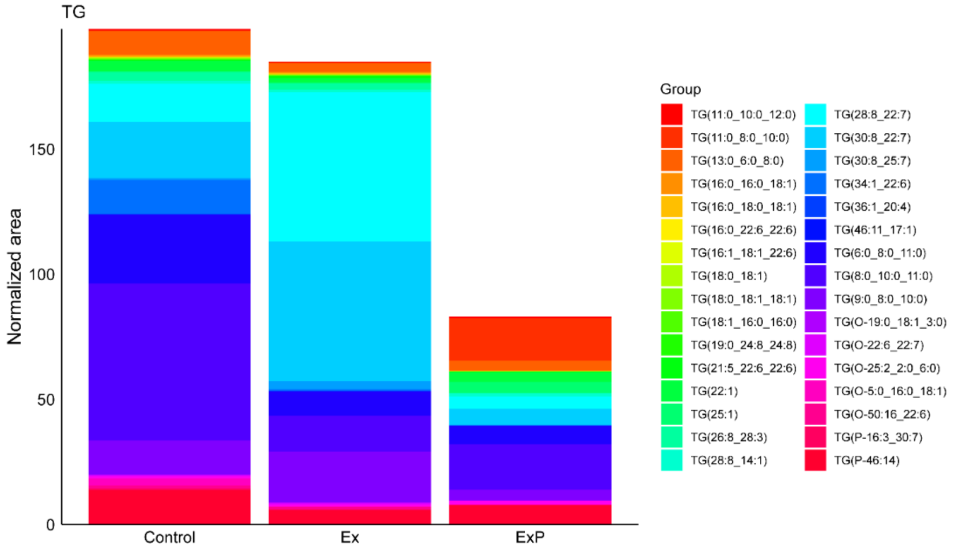

m

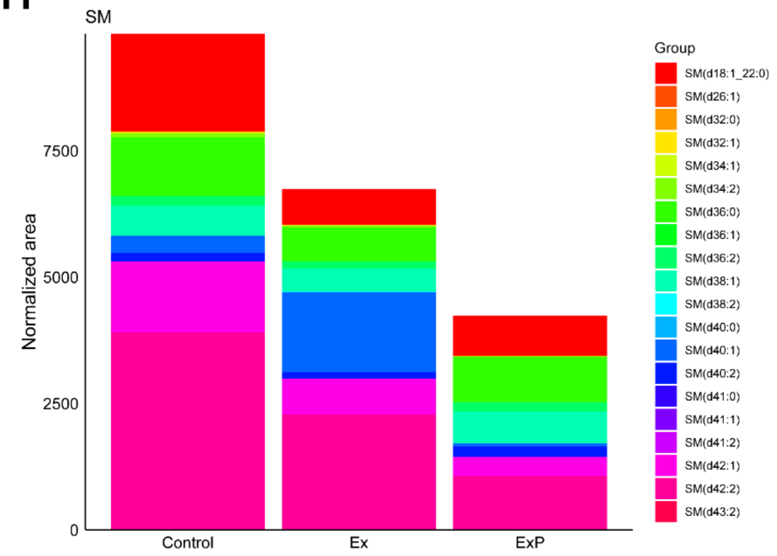

Supplementary Figure 3 (continued). Lipidomics results* l. Triacylglycerol TG-type lipids. m. Sphingomyelin (SM)-type lipids. *: The detailed information of detected lipids and significantly changed lipids among Control, Ex, and ExP groups is shown in **Table S4**.

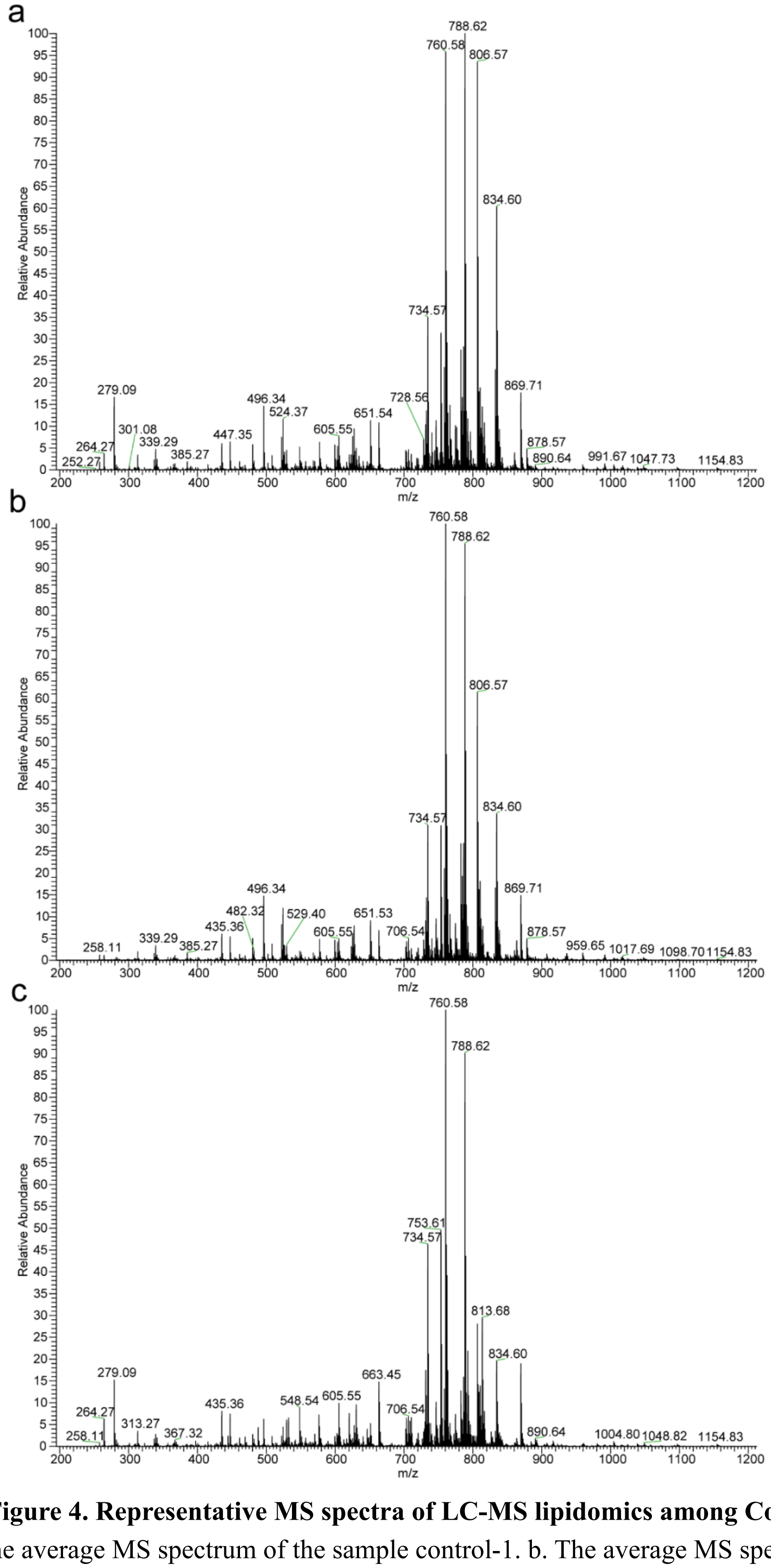

Supplementary Figure 4. Representative MS spectra of LC-MS lipidomics among Control, Ex, and ExP groups. a. The average MS spectrum of the sample control-1. b. The average MS spectrum of the sample Ex-1. c. The average MS spectrum of the sample ExP-1. The average MS spectra was generated by selecting the entire time region of the TIC using Qual Browser (Thermo Scientific).

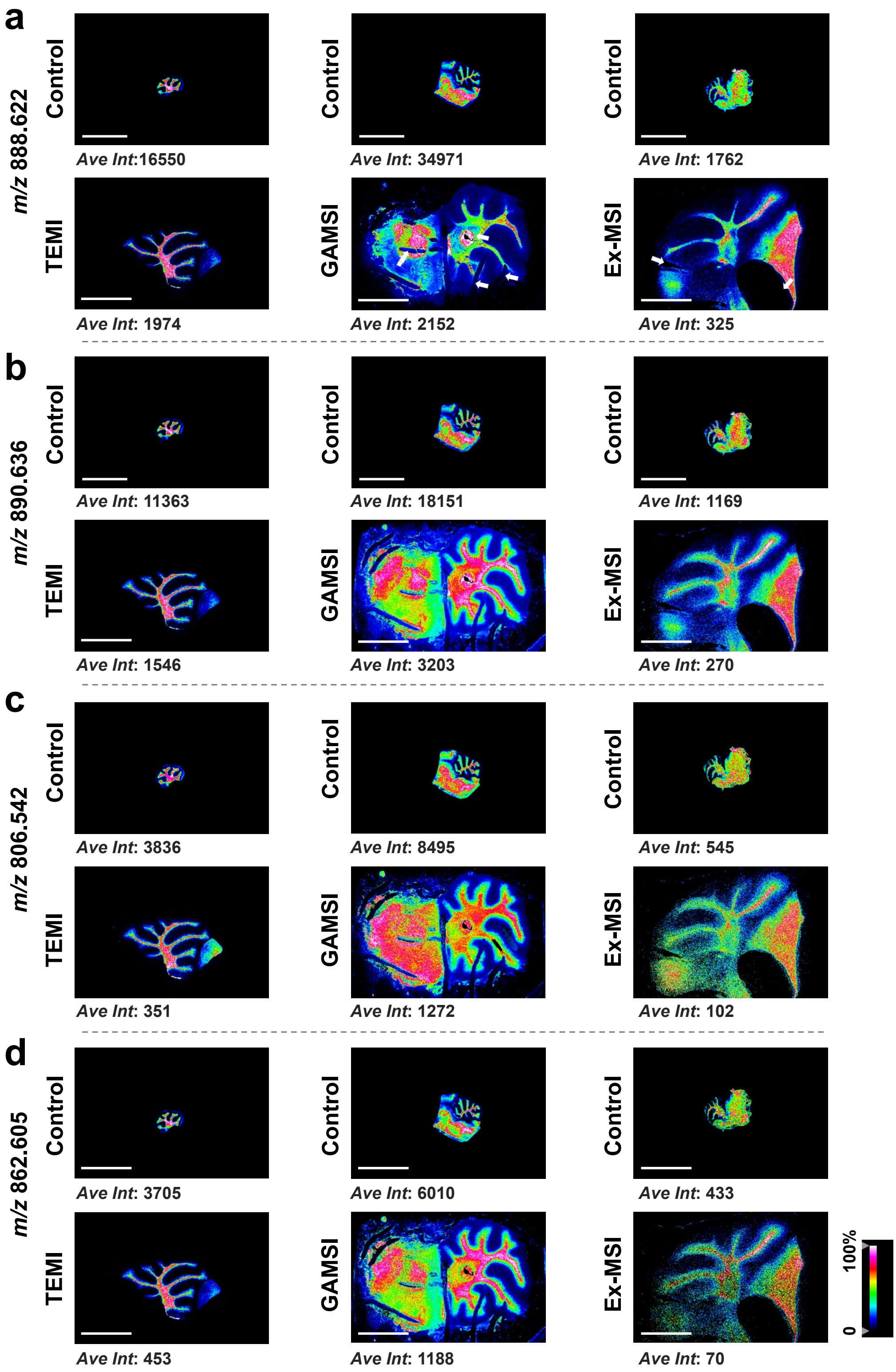

Supplementary Figure 5. Ion images of dominant lipid species obtained from the mouse cerebellum using different methodologies including TEMI (left), GAMSI (middle), and Ex-MSI (right). The ion images of a. SHexCer (42:2;O2) ([M–H]$^-$, *m/z* 888.622). b. PE (46:6;O) ([M–H]$^-$, *m/z* 890.636). c. PE (40:6;O) ([M–H]$^-$, *m/z*806.542). d. SHexCer (40:1;O2) ([M–H]$^-$, *m/z* 862.605). The scale bar is 6 mm, and all the experiments were carried out under 50 μm laser beam raster scanning. The "*Ave Int*" at the bottom of each ion image denotes the average signal intensity of the corresponding ion in the tissue region.

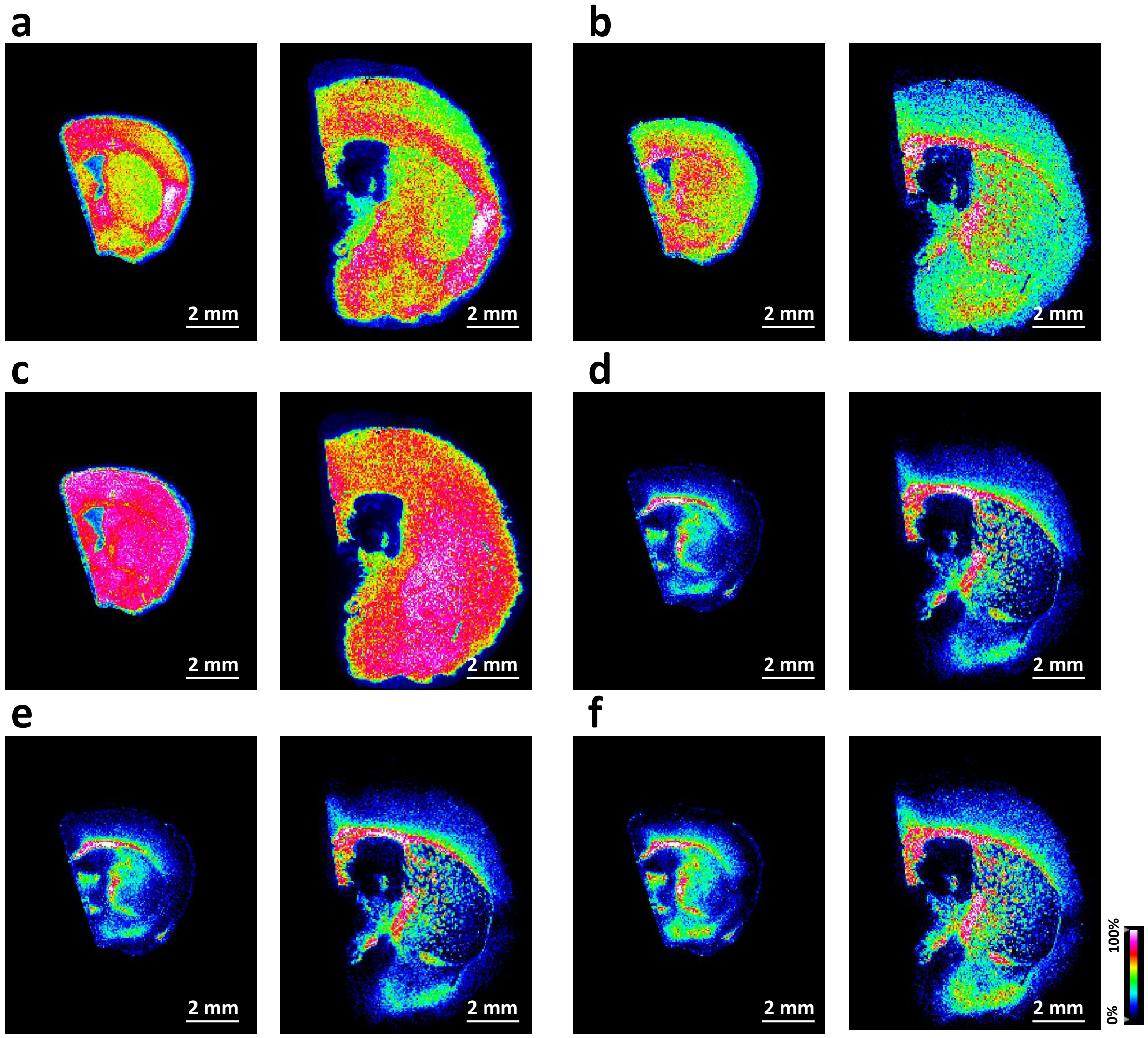

Supplementary Figure 6. MS imaging of a half mouse brain coronal tissue expanded with a single round of gel embedding. MS images of representative lipid species detected from control (left panel) and tissue expansion treated coronal tissue section (right panel). a. MS image of PC (32:1) ($[M + H]^+$, *m/z* 732.552). b. MS image of PC (34:5) ($[M + H]^+$, *m/z* 752.532). c. MS image of PC (34:1) ($[M + H]^+$, *m/z* 760.593). d. MS image of PC(O-40:5) ($[M + H]^+$, *m/z* 822.645). e. MS image of PE (42:0) ($[M + H]^+$, *m/z* 832.672). f. MS image of PC (O-42:6) ($[M + H]^+$, *m/z* 848.660).

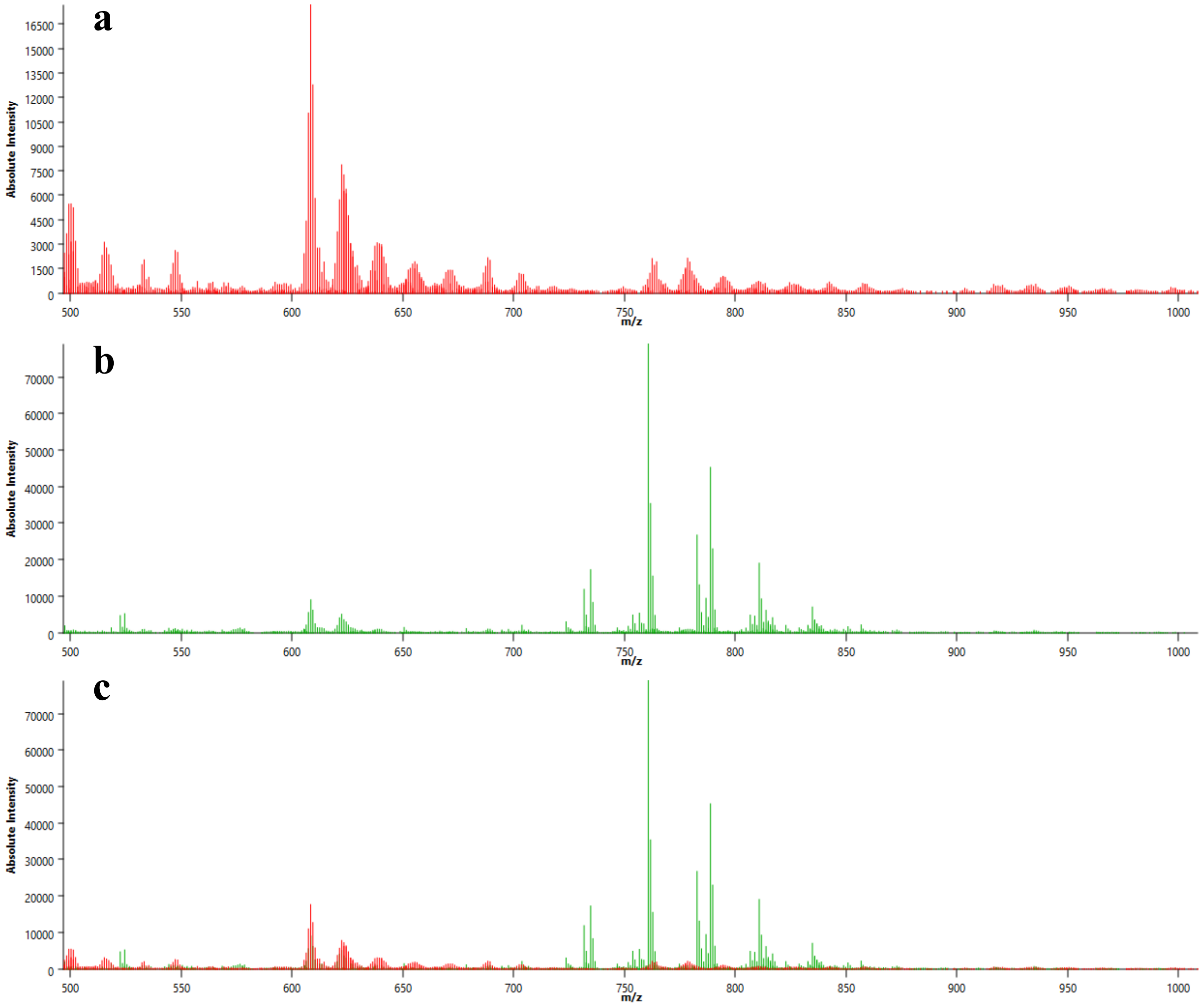

Supplementary Figure 7. Representative mass spectra from surrounding gelatin region and ~3.5-fold expanded tissue region under positive mode with DAN matrix. a. The gelatin region, in which the dominant peaks observed are from the DAN matrix. b. ~3.5-fold expanded tissue region, in which the dominant peaks observed are lipids species from the tissue; c. overlapped spectra from the gelatin region (red) and tissue region (green).

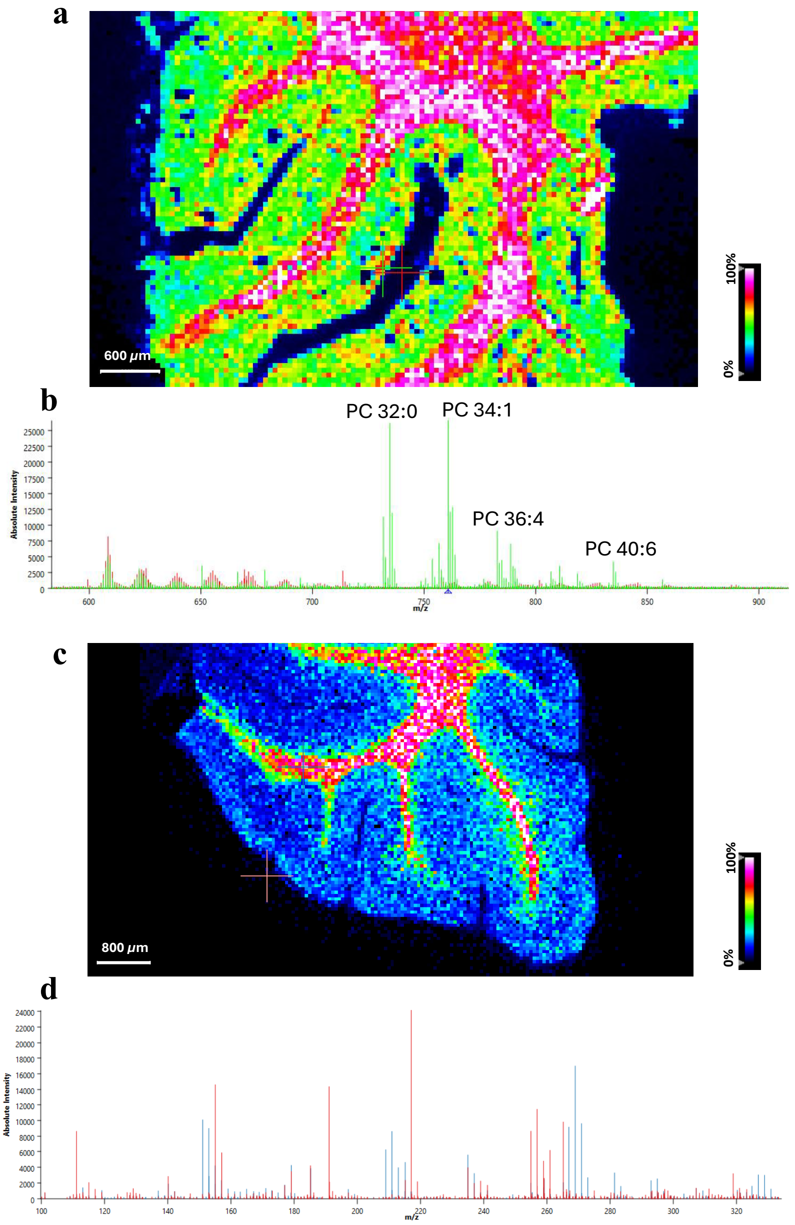

Supplementary Figure 8. TEMI of mouse cerebellum tissue. a. The MSI image of PC (34:1) ($[M+H]^+$, *m/z* 760.5901) from a ~2.5-fold linear expanded mouse cerebellum using DAN matrix under positive ionization mode, the scale bar is 600 μm. b. The representative single mass spectrum from the tissue regions (in green color) and its adjacent blank hydrogel area (in red color), corresponding single spectrum from the pixels highlighted on the above MS image. c. The MSI image of FA (18:1) ($[M-H]^-$, *m/z* 281.2498) from a ~2.5-fold linear expanded mouse cerebellum using NEDC matrix under negative ionization mode, the scale bar is 800 μm. d. The representative single mass spectrum from the tissue regions (in blue color) and its adjacent blank hydrogel area (in red color), corresponding single spectrum from the pixels highlighted on the above MS image. It is noted that high background signals from the MALDI matrix were observed during MS imaging of the tissue in the low mass range; however, most target small biomolecules were still resolvable due to the high mass resolution. Target lipid and small metabolite signals were solely observed from the tissue area whereas no signal of these lipids or metabolites was observed in the adjacent blank hydrogel area, indicating the delocalization of these biomolecules was minimal. MALDI-MSI performed with a 50 μm laser beam raster scanning.

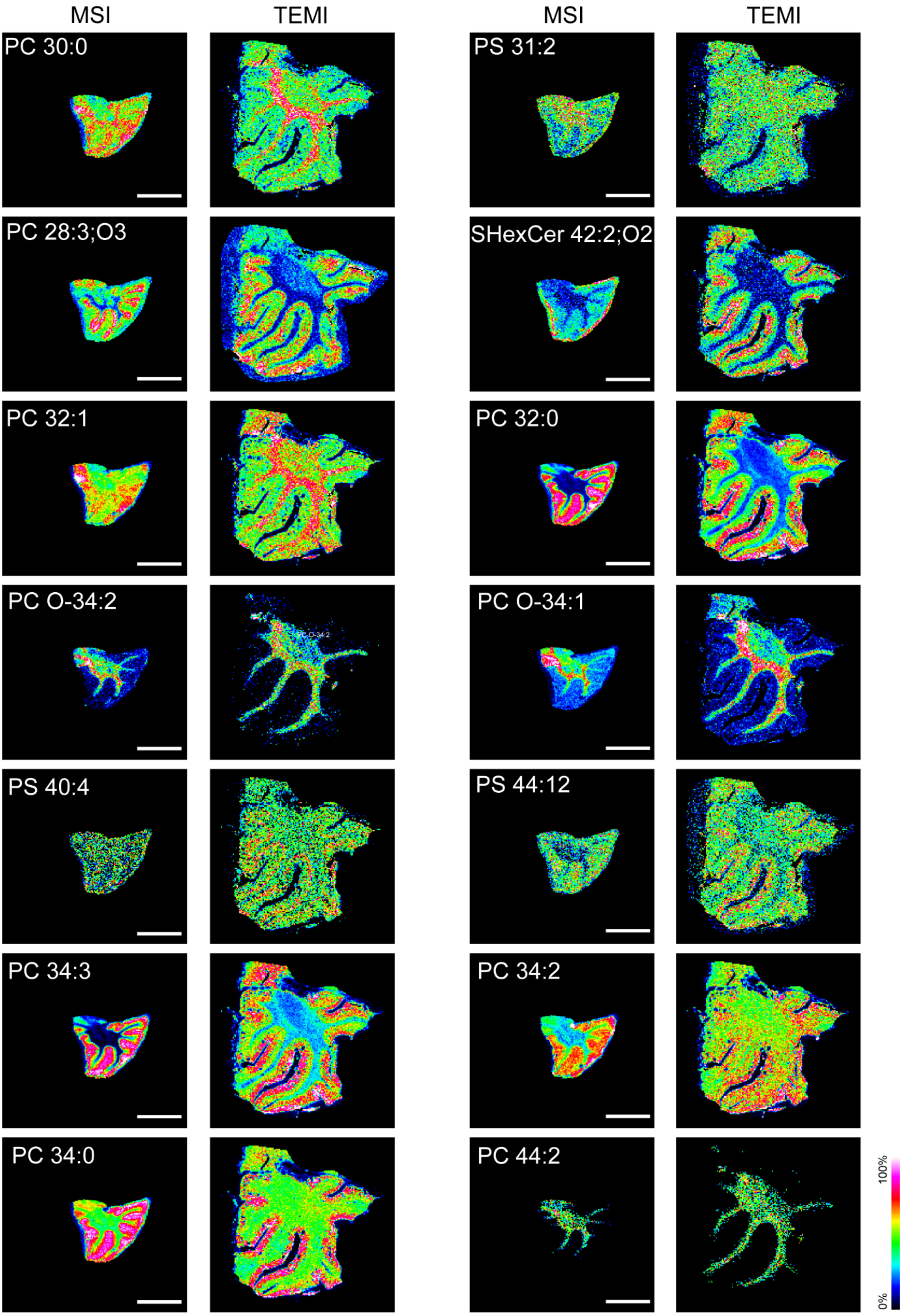

Supplementary Figure 9*. MS imaging of ~2.5-fold linear expanded mouse cerebellum tissue under positive mode. MSI: MS imaging of unexpanded tissue, TEMI: MS imaging of expanded tissue. *: Detailed information in **Supplementary Table 1**. The scale bar is 2 mm.

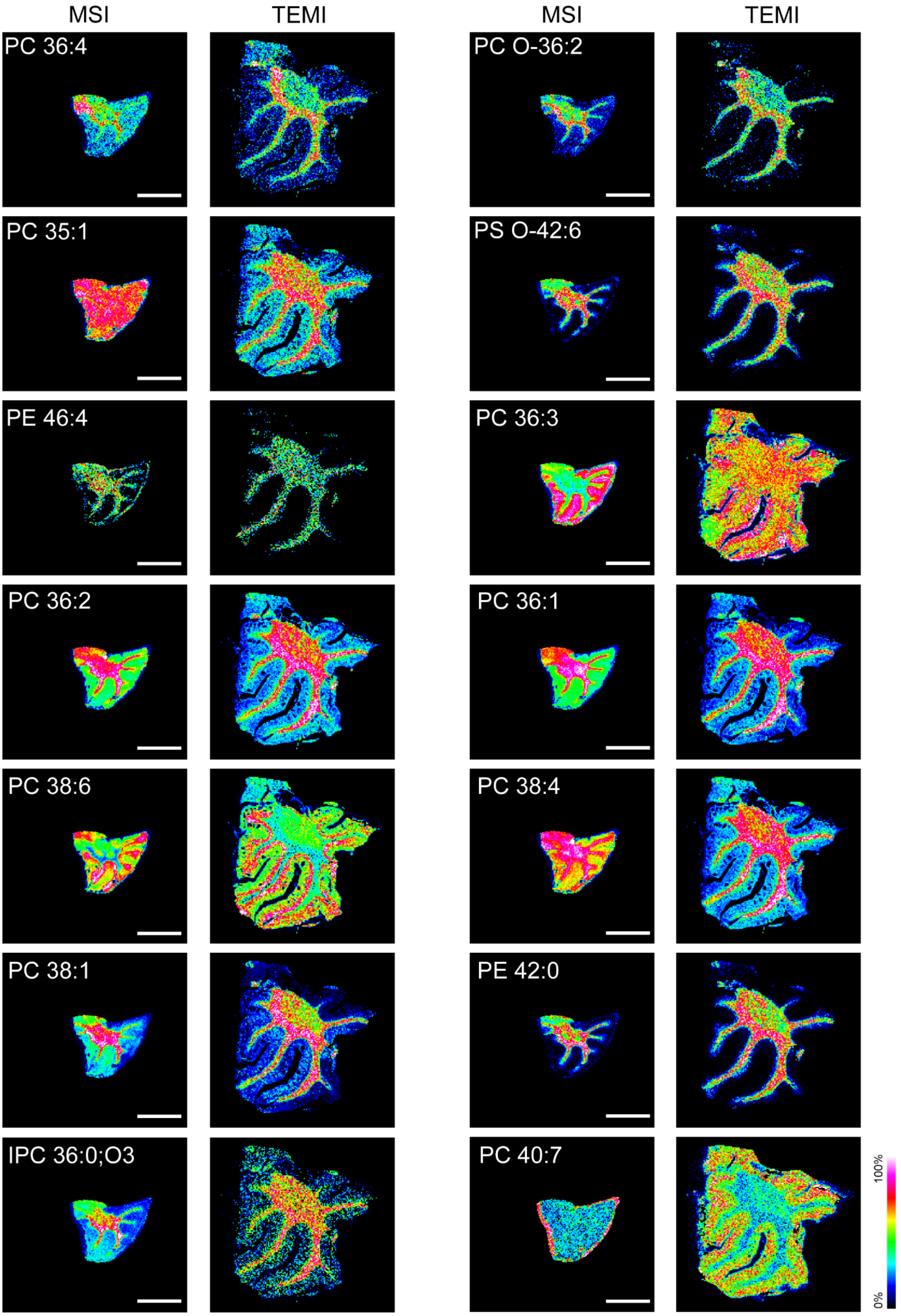

Supplementary Figure 9*. (continued) MS imaging of ~2.5-fold linear expanded mouse cerebellum tissue under positive mode. MSI: MS imaging of unexpanded tissue, TEMI: MS imaging of expanded tissue. *: Detailed information in **Supplementary Table 1**. The scale bar is 2 mm.

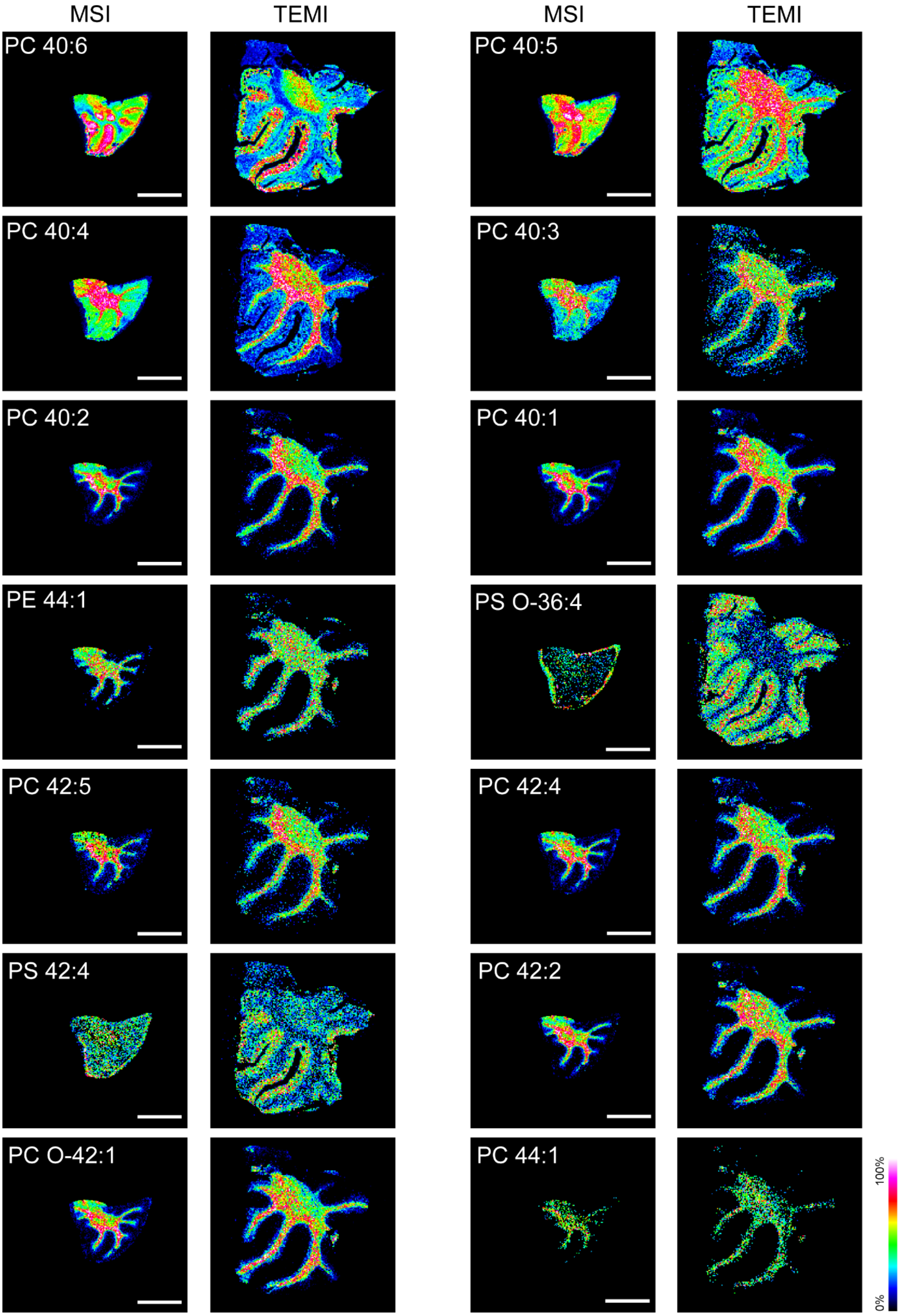

Supplementary Figure 9*. (continued) MS imaging of ~2.5-fold linear expanded mouse cerebellum tissue under positive mode. MSI: MS imaging of unexpanded tissue, TEMI: MS imaging of expanded tissue. *: Detailed information in **Supplementary Table 1**. The scale bar is 2 mm.

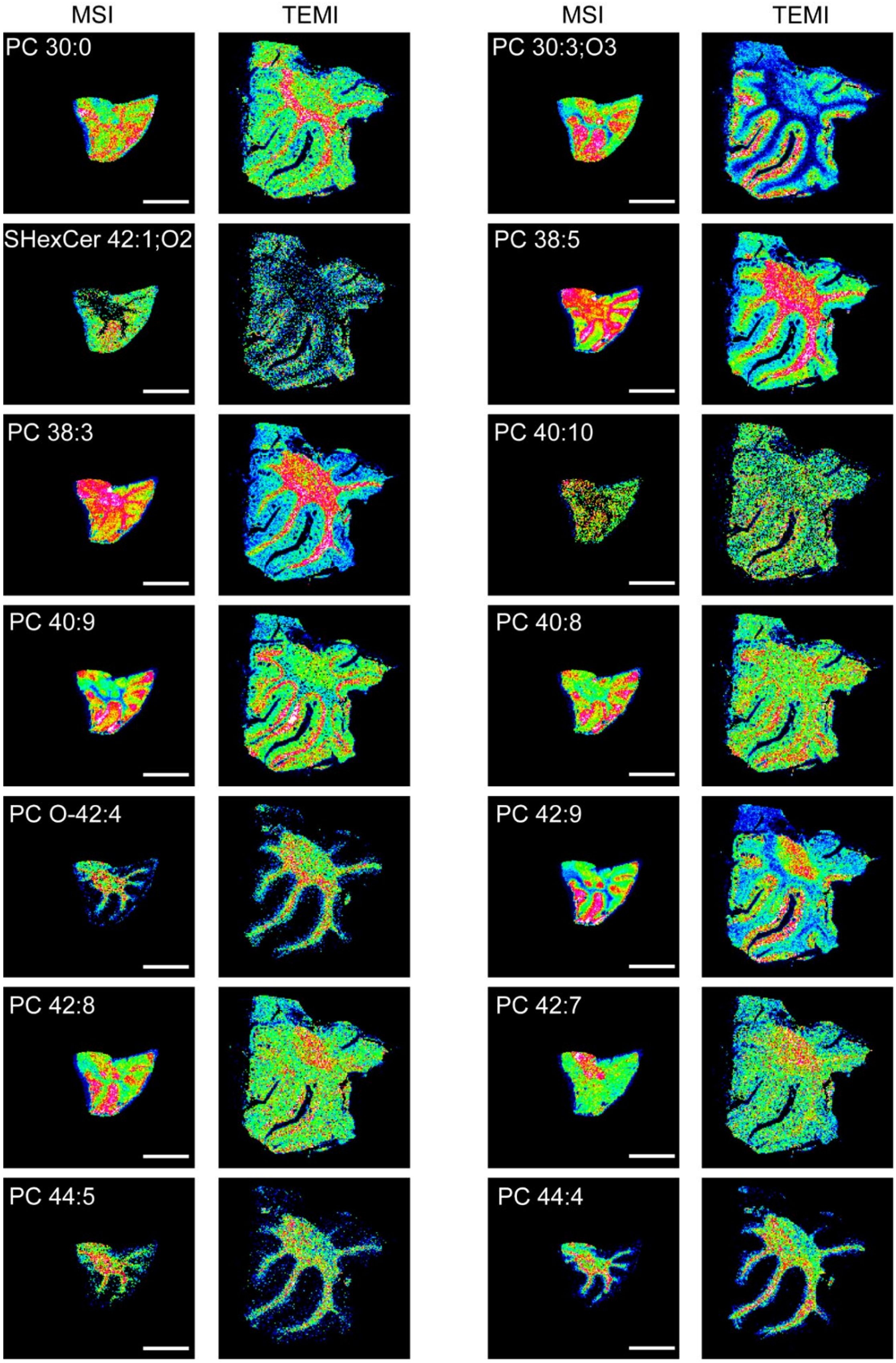

Supplementary Figure 9*. (continued) MS imaging of ~2.5-fold linear expanded mouse cerebellum tissue under positive mode. MSI: MS imaging of unexpanded tissue, TEMI: MS imaging of expanded tissue. *: Detailed information in **Supplementary Table 1**. The scale bar is 2 mm.

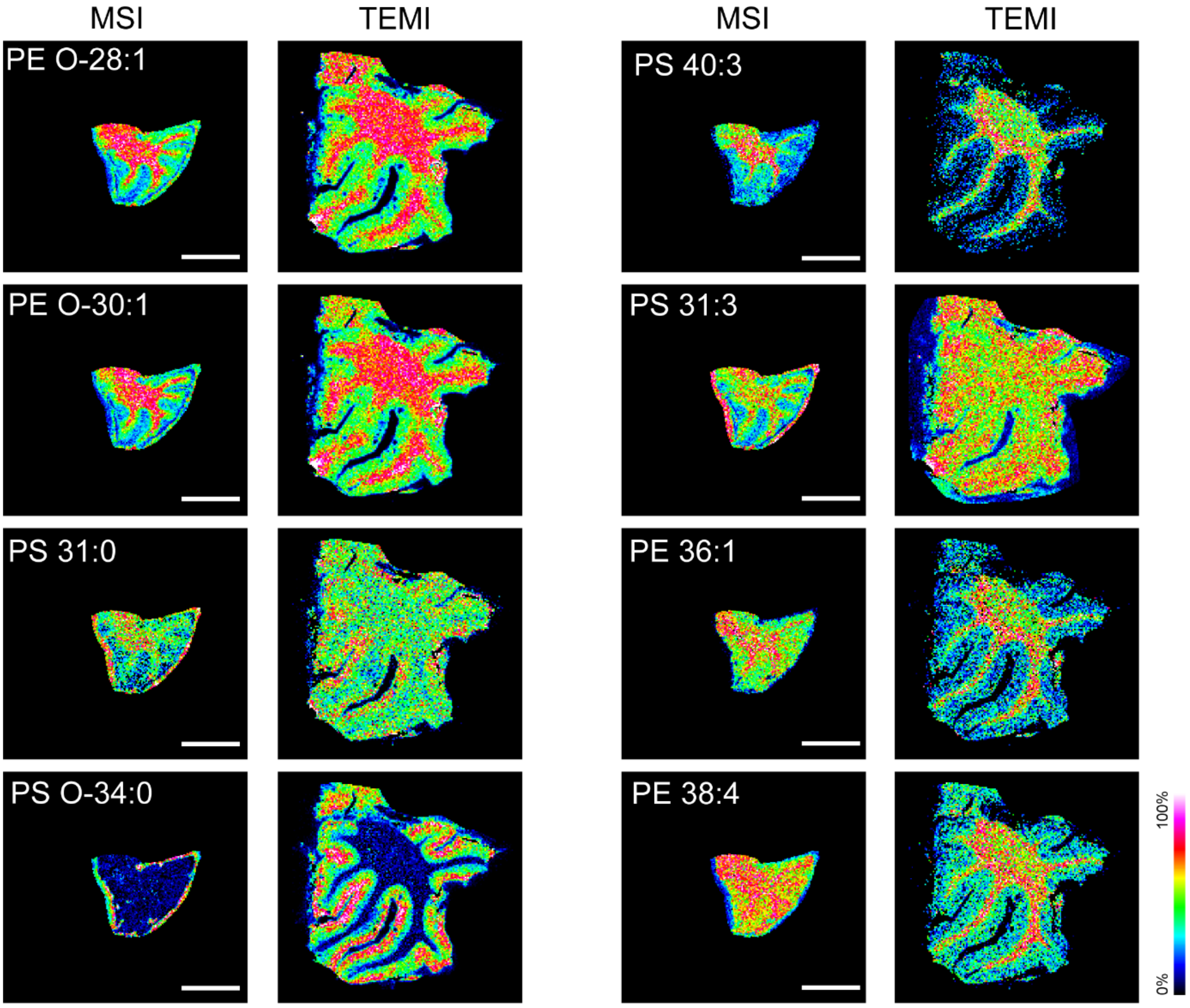

Supplementary Figure 9*. (continued) MS imaging of ~2.5-fold linear expanded mouse cerebellum tissue under positive mode. MSI: MS imaging of unexpanded tissue, TEMI: MS imaging of expanded tissue. *: Detailed information in **Supplementary Table 1**. The scale bar is 2 mm.

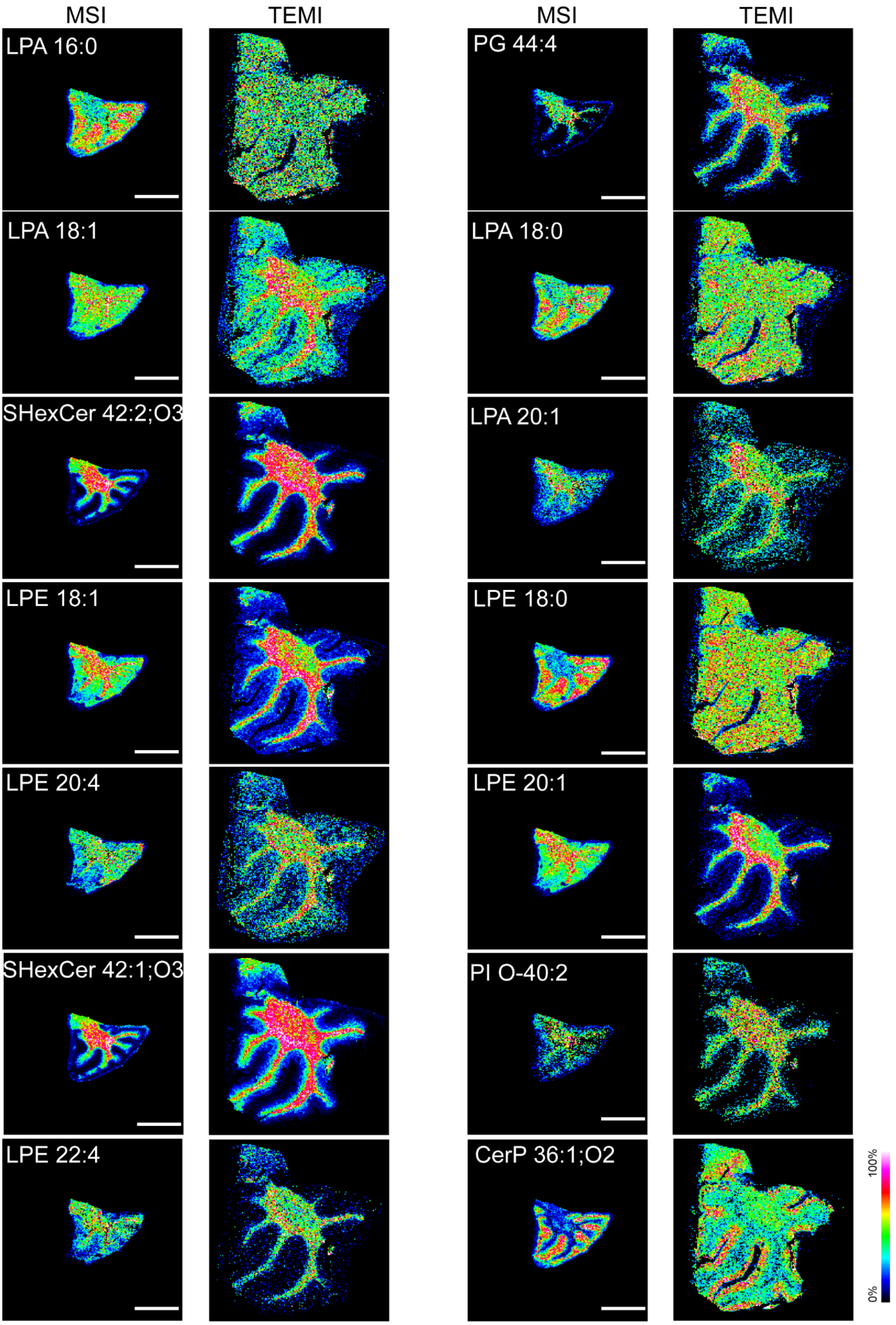

Supplementary Figure 10*. MS imaging of ~2.5-fold linear expanded mouse cerebellum tissue under negative mode. MSI: MS imaging of unexpanded tissue, TEMI: MS imaging of expanded tissue. *: Detailed information in **Supplementary Table 2**. The scale bar is 2 mm.

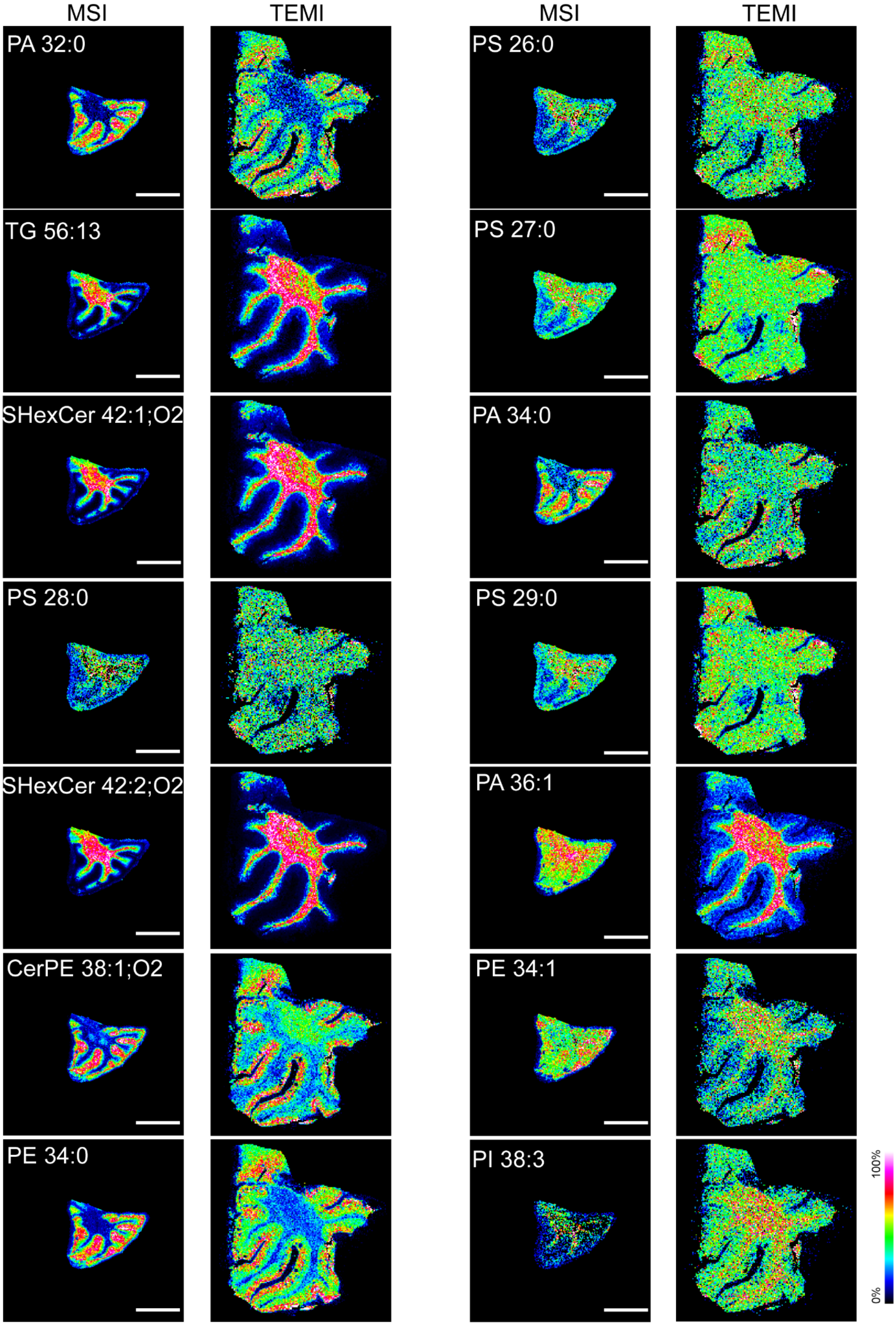

Supplementary Figure 10*. MS imaging of ~2.5-fold linear expanded mouse cerebellum tissue under negative mode. MSI: MS imaging of unexpanded tissue, TEMI: MS imaging of expanded tissue. *: Detailed information in **Supplementary Table 2**. The scale bar is 2 mm.

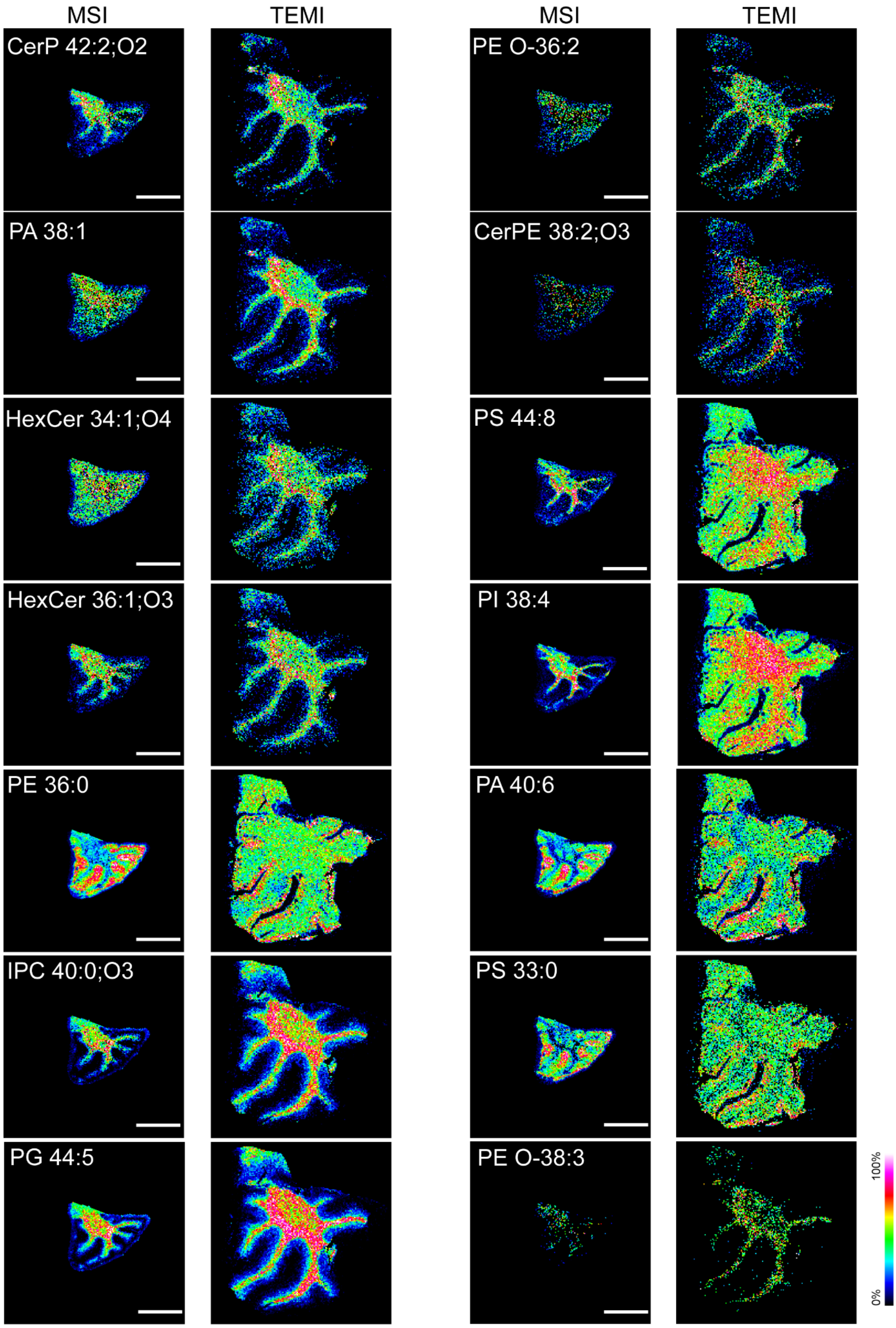

Supplementary Figure 10*. MS imaging of ~2.5-fold linear expanded mouse cerebellum tissue under negative mode. MSI: MS imaging of unexpanded tissue, TEMI: MS imaging of expanded tissue. *: Detailed information in **Supplementary Table 2**. The scale bar is 2 mm.

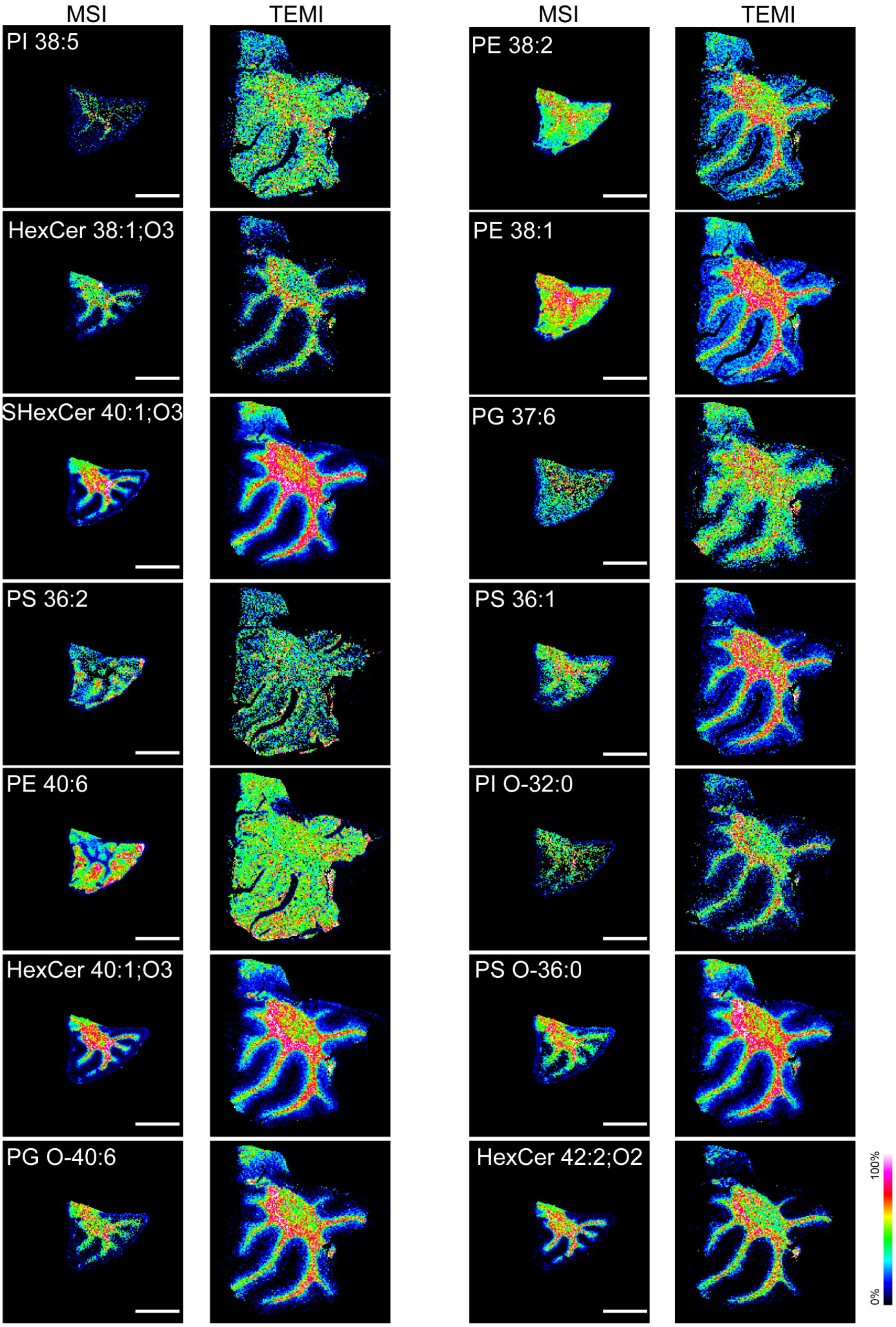

Supplementary Figure 10*. MS imaging of ~2.5-fold linear expanded mouse cerebellum tissue under negative mode. MSI: MS imaging of unexpanded tissue, TEMI: MS imaging of expanded tissue. *: Detailed information in **Supplementary Table 2**. The scale bar is 2 mm.

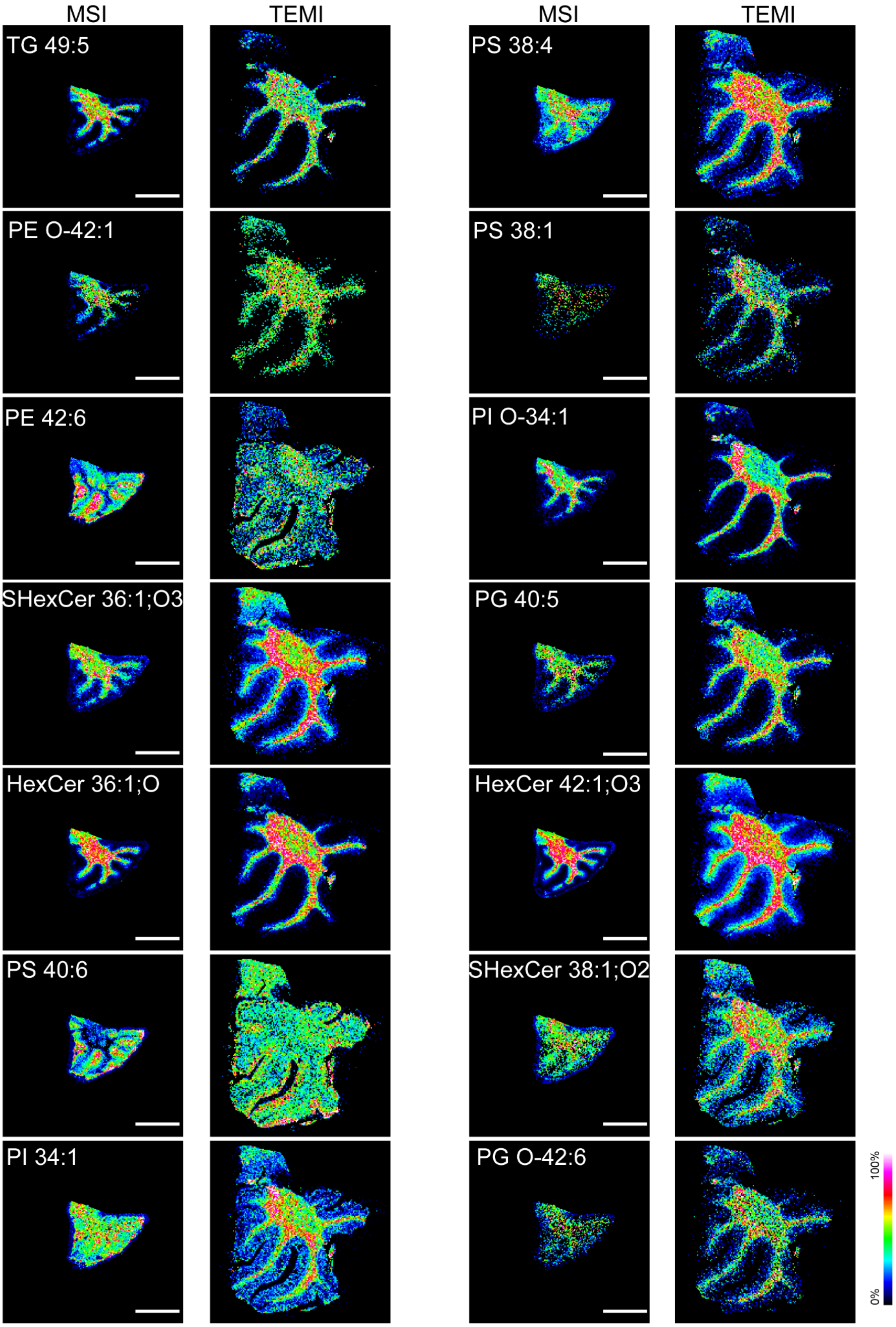

Supplementary Figure 10*. MS imaging of ~2.5-fold linear expanded mouse cerebellum tissue under negative mode. MSI: MS imaging of unexpanded tissue, TEMI: MS imaging of expanded tissue. *: Detailed information in **Supplementary Table 2**. The scale bar is 2 mm.

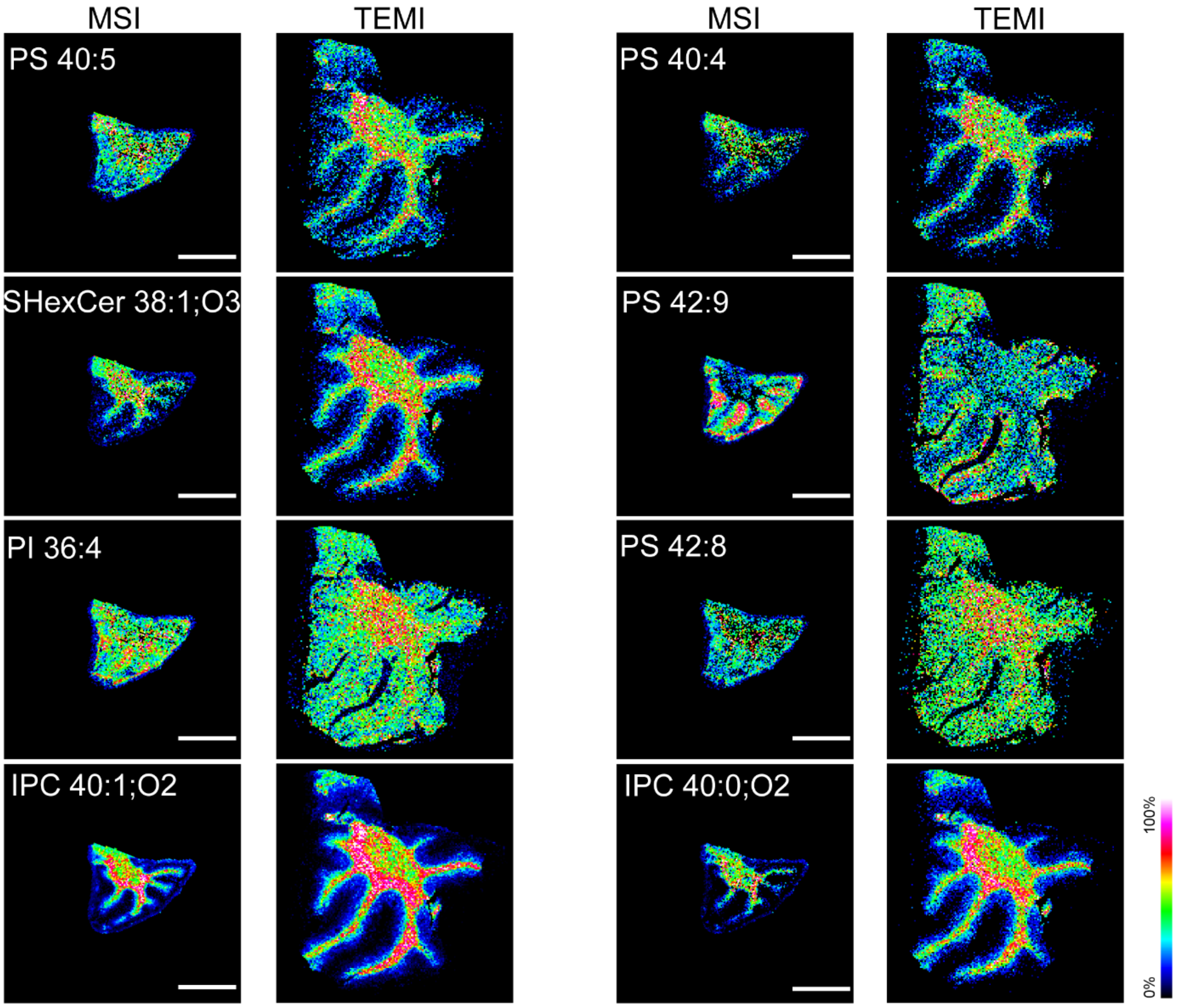

Supplementary Figure 10*. MS imaging of ~2.5-fold linear expanded mouse cerebellum tissue under negative mode. MSI: MS imaging of unexpanded tissue, TEMI: MS imaging of expanded tissue. *: Detailed information in **Supplementary Table 2**. The scale bar is 2 mm.

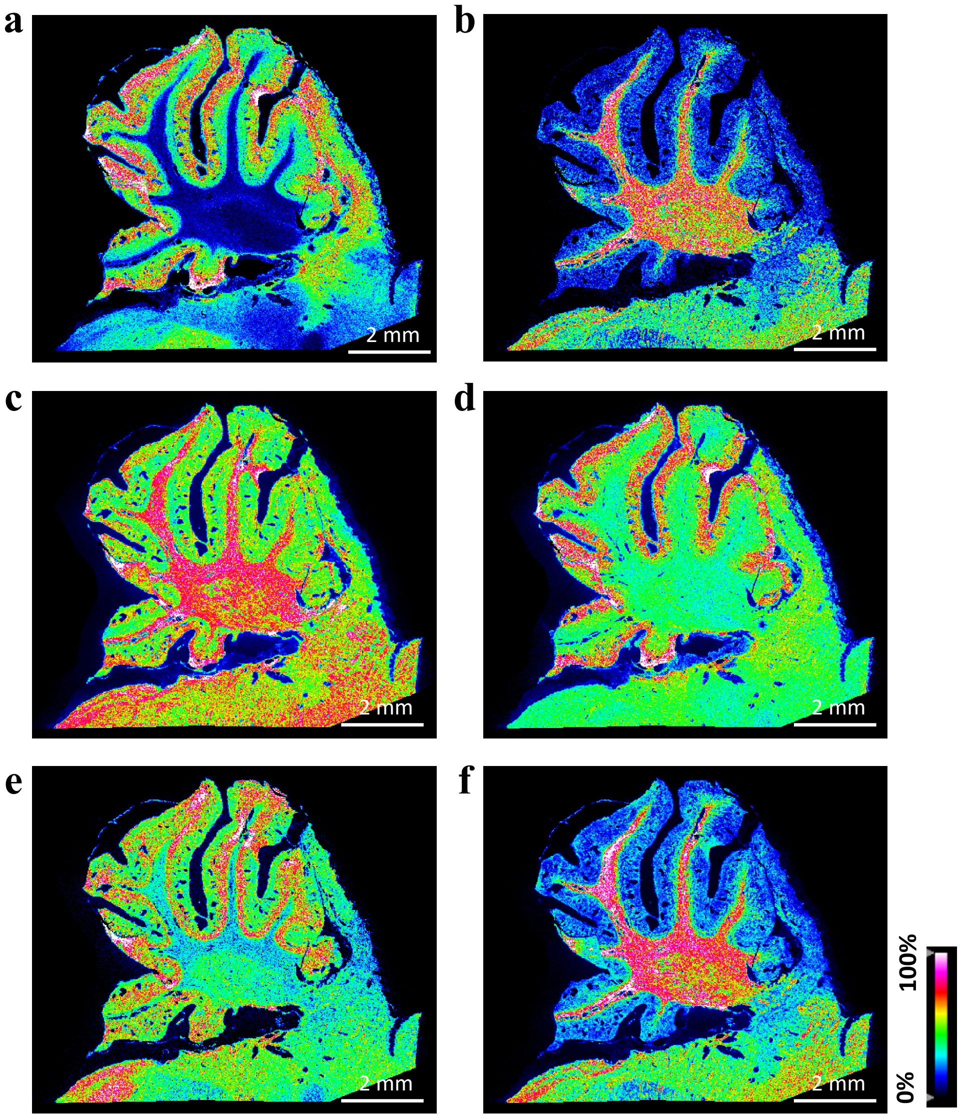

Supplementary Figure 11. Representative TEMI images of a ~2.5 linearly expanded mouse cerebellum tissue through a 20 µm laser beam raster scanning under positive mode. a. PC (32:0) ($[M + H]^+$, *m/z* 734.5694). b. PC (34:5) ($[M + H]^+$, *m/z* 752.529). c. PC (34:1) ($[M + H]^+$, *m/z* 760.586). d. PC (34:0) ($[M + H]^+$, *m/z* 762.597). e. PC (38:6) ($[M + H]^+$, *m/z* 806.565). f. PC (38:4) ($[M + H]^+$, *m/z* 810.596). The MSI images obtained with mass error tolerance of 10 ppm.

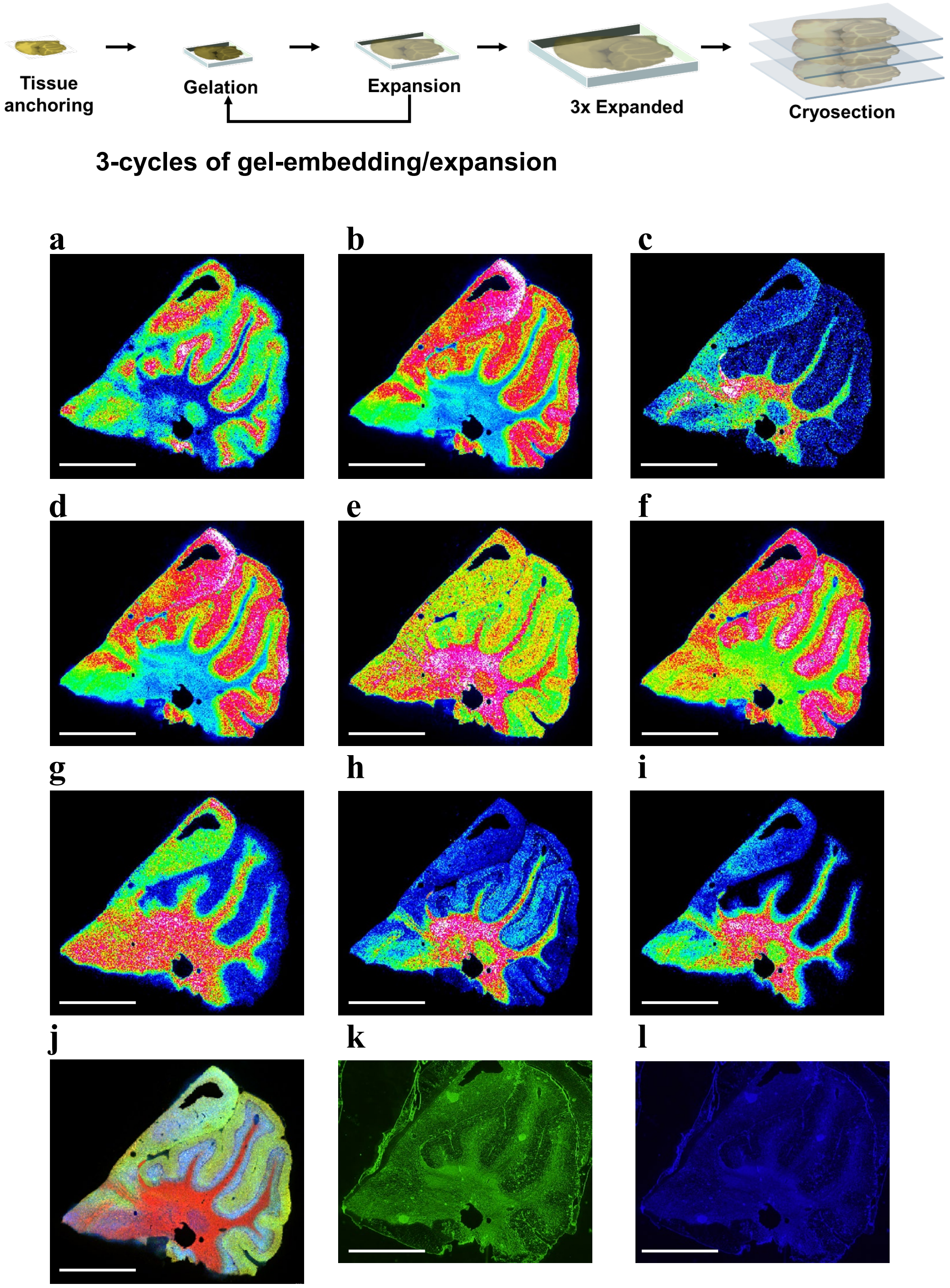

Supplementary Figure 12. TEMI results from mouse cerebellum tissue with 3 times gel-embedding and expansion treatment. On the top is the workflow of the 3 times gel-embedding-expansion with the cerebellum tissue; on the bottom are the TEMI and fluorescent images obtained from the ~3.5-fold-expanded cerebellum tissue section, including a. PC 28:3;O3 (*m/z* 720.432). b. PC 32:0 (*m/z* 734.567). c. PC O-34:1 (*m/z* 746.602). d. PC 34:3 (*m/z* 756.548). e. PC 34:1 (*m/z* 760.584). f. PC 34:0 (*m/z* 762.595). g. PE 38:7;O (*m/z* 778.509). h. PC 38:1 (*m/z* 816.646). i. PC O-40:5 (*m/z* 822.638). j. Overlay of TEMI images of PC 28:3;O3 (*m/z* 720.432) (blue), PC 32:0 (*m/z* 734.567) (green), and PC 34:1 (*m/z* 760.584) (red). k. Alexa Fluor 488 staining fluorescent image of the expanded cerebellum tissue. l. DAPI staining fluorescent image of the expanded cerebellum tissue. The MSI experiments were carried out using a 50 μm laser raster scanning and the MSI images obtained with mass error tolerance of 10 ppm. The scale bar is 4 mm.

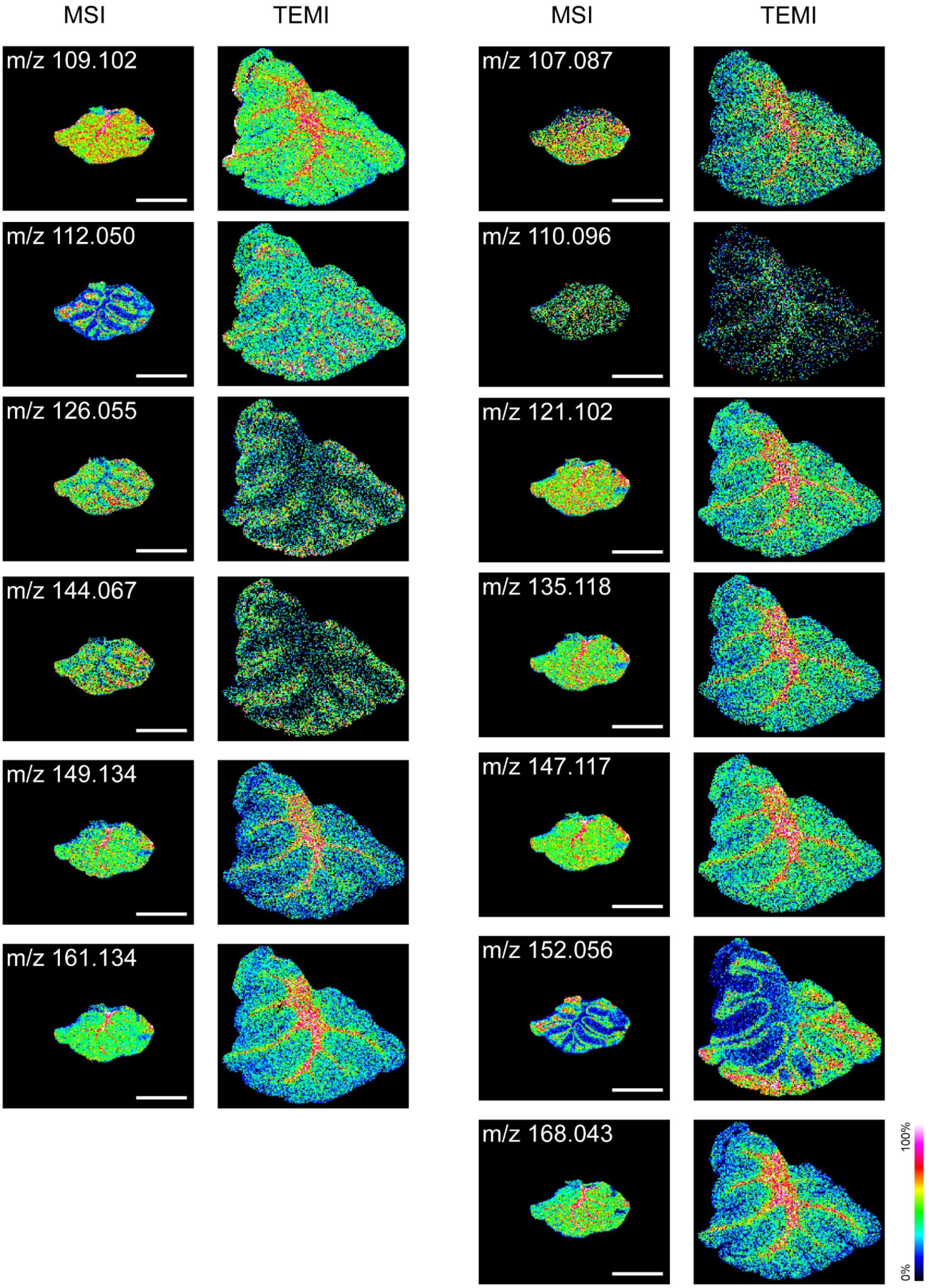

Supplementary Figure 13. **Representative mass spectrometry images of the other unknown small molecules observed from the ~2.5-fold linearly expanded mouse cerebellum under positive mode, the control (left panel, MSI) and tissue expansion treated (right panel, TEMI), *m/z* [M+H]$^+$.** The MSI experiments were carried out using a 50 µm laser raster scanning and the MSI images obtained with mass error tolerance of 10 ppm. The scale bar is 2 mm.

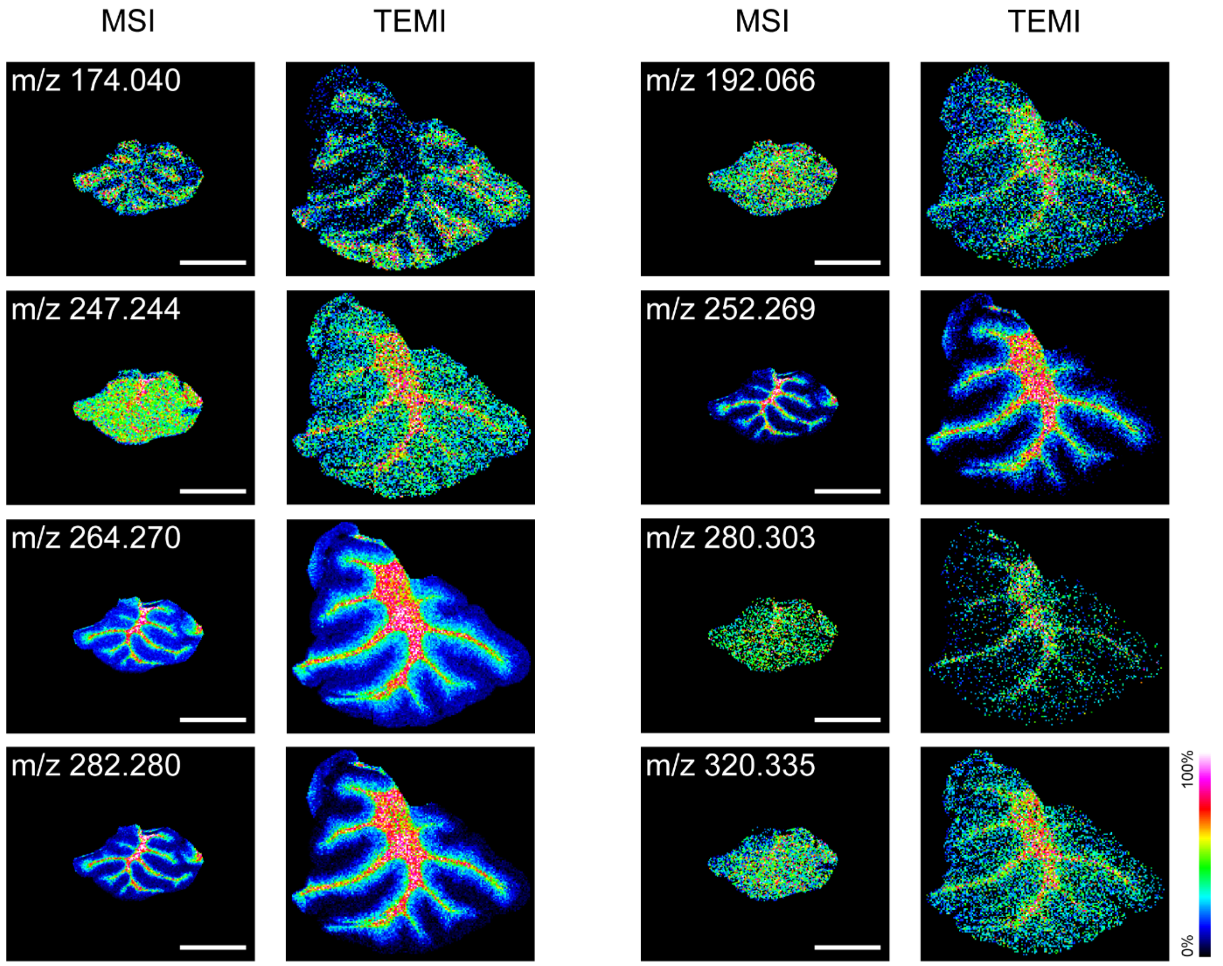

Supplementary Figure 13. (Continued) Representative of other unknown small molecules observed from the ~2.5-fold linearly expanded mouse cerebellum under positive mode, the control (left panel, MSI) and tissue expansion treated (right panel, TEMI). The MSI experiments were carried out using a 50 μm laser raster scanning and the MSI images obtained with mass error tolerance of 10 ppm. The scale bar is 2 mm.

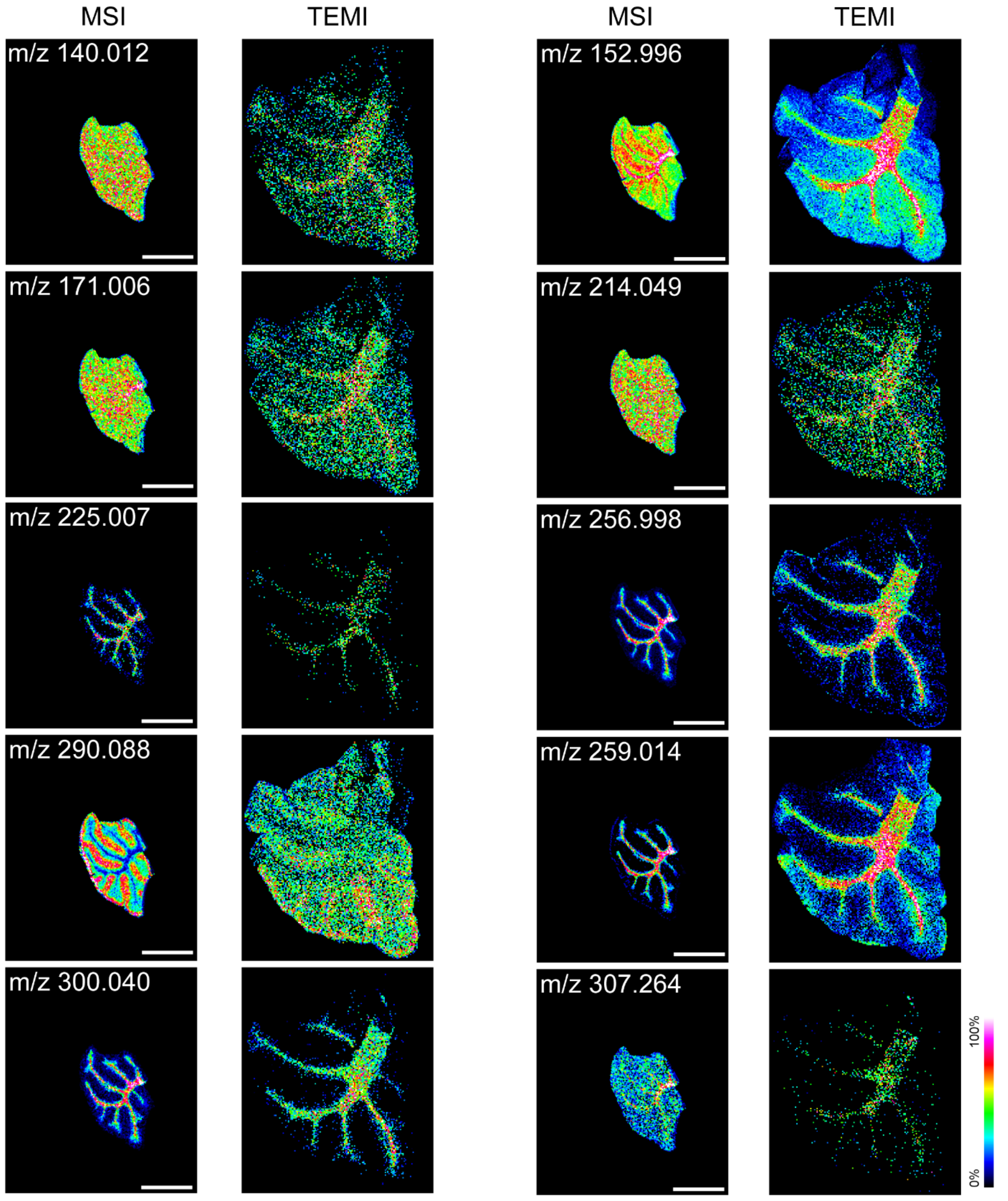

Supplementary Figure 14. **Representative mass spectrometry images of other unknown small molecules observed from the ~2.5-fold linearly expanded mouse cerebellum under negative mode, the control (left panel, MSI) and tissue expansion treated (right panel, TEMI).** The MSI experiments were carried out using a 50 μm laser raster scanning and the MSI images obtained with mass error tolerance of 10 ppm. The scale bar is 2 mm.

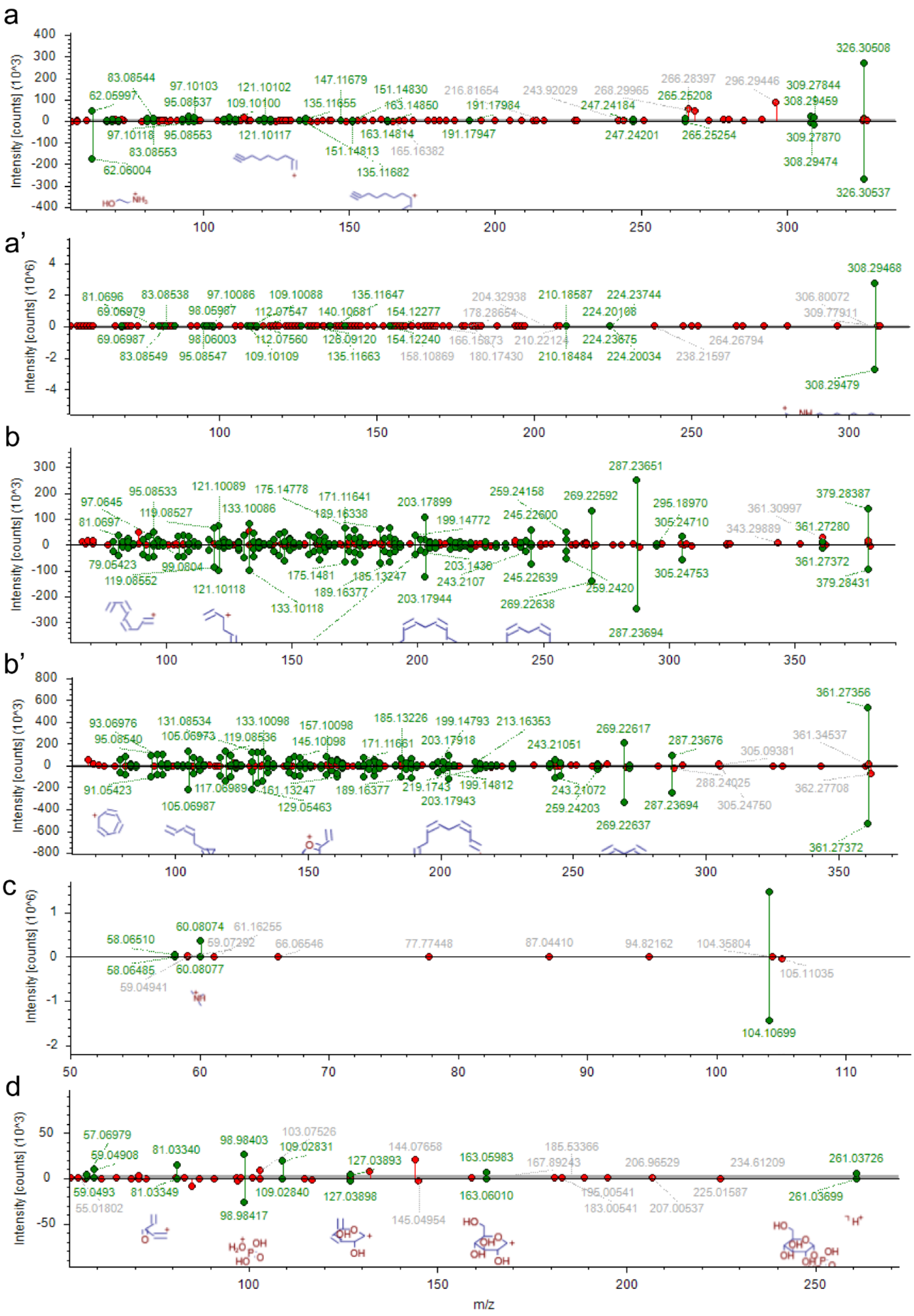

Supplementary Figure 15. The mirror plots of MS2 matching between fragmentation scan of our study on the top and the matched reference scan from the mzCloud database on the bottom: a. Oleoyl ethanolamide (OEA) (Best Match Score: 99.9) $[OEA + H]^+$, a'. $[OEA - H_2O + H]^+$; b. 2-arachidonoyl glycerol (2-AG) (Best Match Score: 83.7) $[2\text{-}AG + H]^+$, b'. $[2\text{-}AG - H_2O + H]^+$; c. Choline (Best Match Score: 94.7); d. Hexose-phosphate. Green: the matched fragments. Red: the unknown fragments.

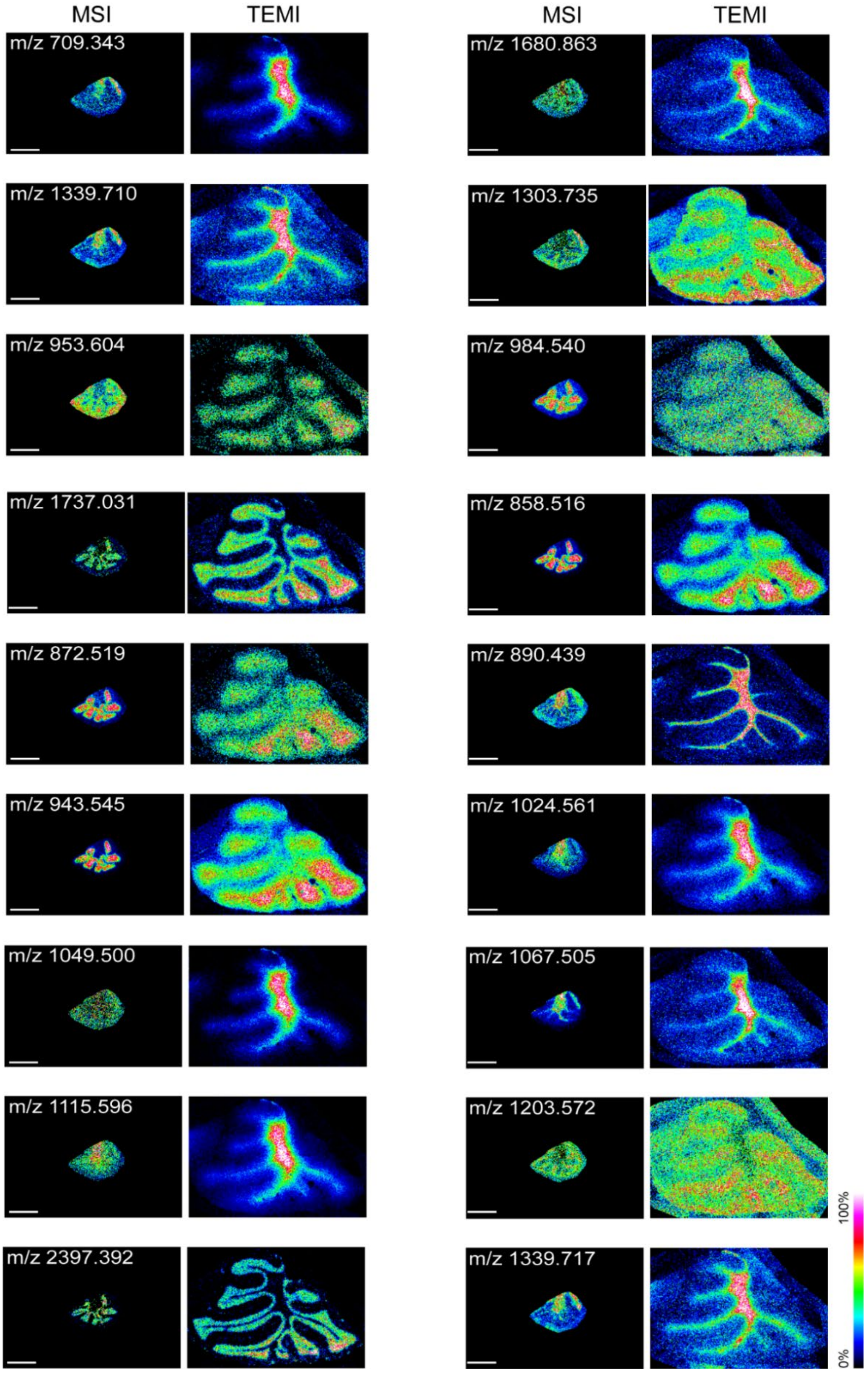

Supplementary Figure 16. Representative MS images of on-tissue digested peptides of ~3.5-fold linearly expanded mouse cerebellum tissue under positive mode. MSI: MS imaging of unexpanded tissue, TEMI: MS imaging of expanded tissue. The *m/z* corresponding peptides information is in **Supplementary Table 4**. The scale bar is 2 mm.

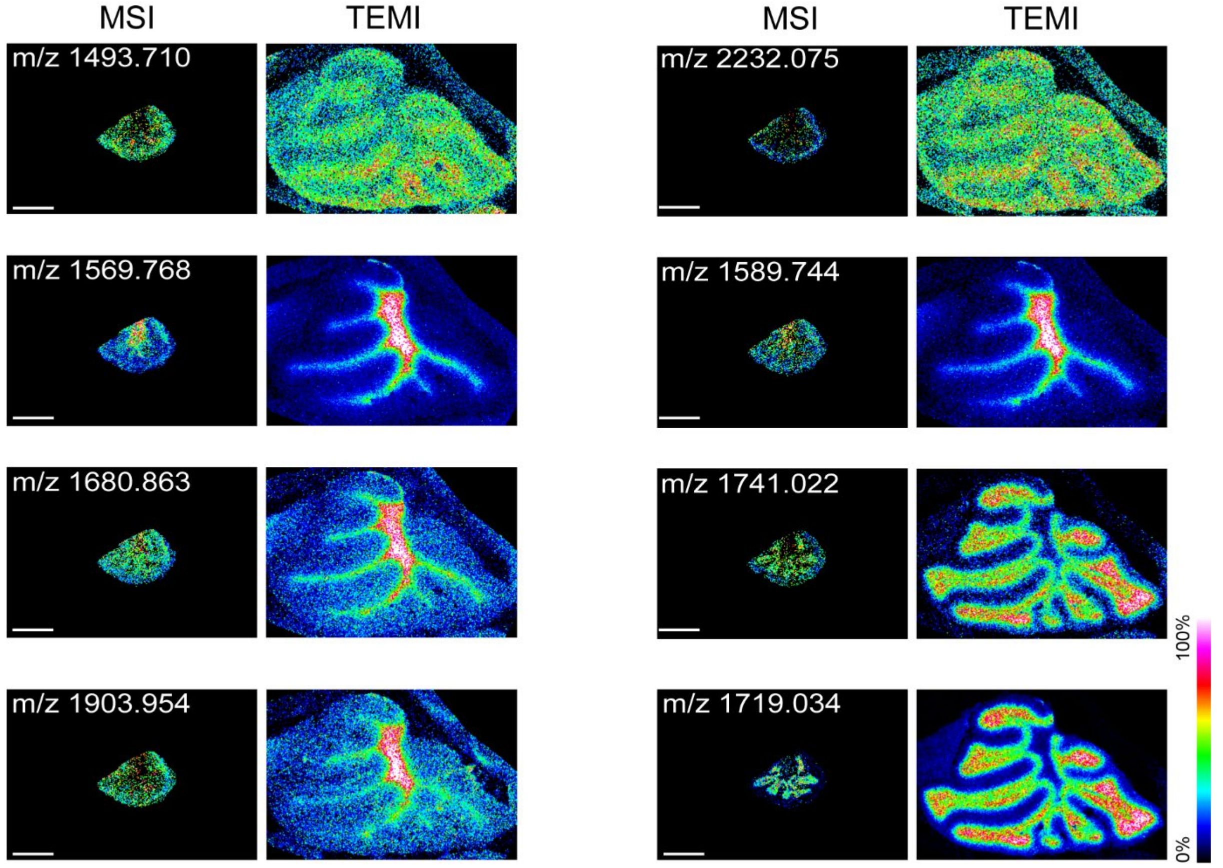

Supplementary Figure 16. (Continued) Representative MS images of on-tissue digested peptides of ~3.5-fold linearly expanded mouse cerebellum tissue under positive mode. MSI: MS imaging of unexpanded tissue, TEMI: MS imaging of expanded tissue. The *m/z* corresponding peptides information is in **Supplementary Table 4**. The scale bar is 2 mm.

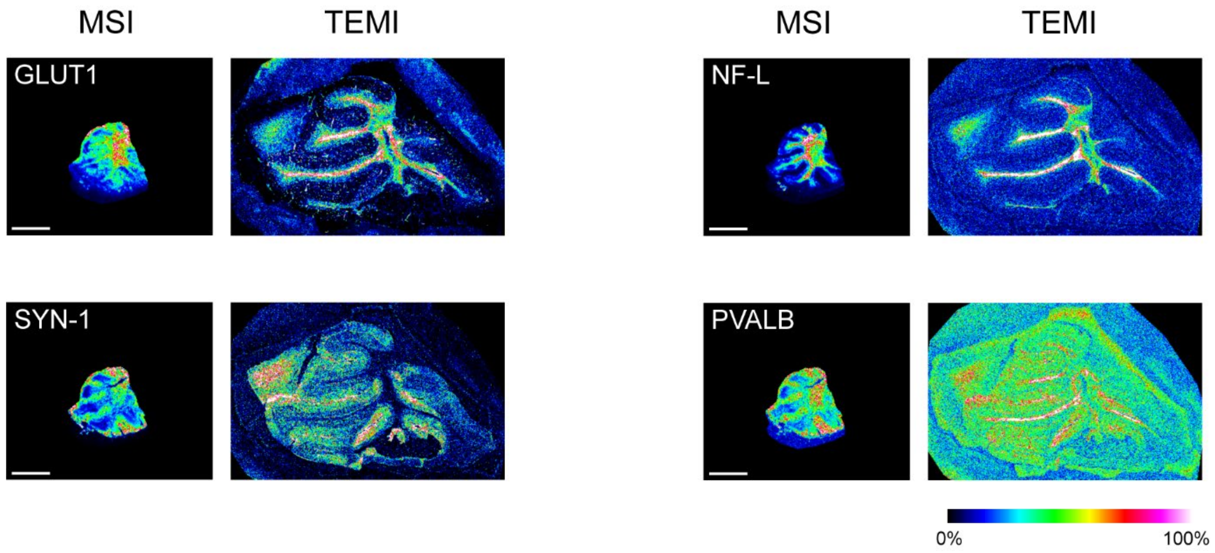

Supplementary Figure 17. Representative MS images of unexpanded and TEMI samples by IHC-MALDI-MSI. Photocleavable antibody mass tags of solute carrier family 2, facilitated MS identification and imaging of glucose transporter member 1 (GLUT1): $[M+H]^+$, *m/z* 856.823; Neurofilament light polypeptide (NF-L): $[M+H]^+$, *m/z* 1346.031; Synapsin-1 (SYN-1): $[M+H]^+$, *m/z* 1483.26; Parvalbumin alpha (PVALB): $[M+H]^+$, *m/z* 1540.283. MSI: MS imaging of unexpanded tissue, TEMI: MS imaging of expanded tissue. The scale bar is 2 mm.

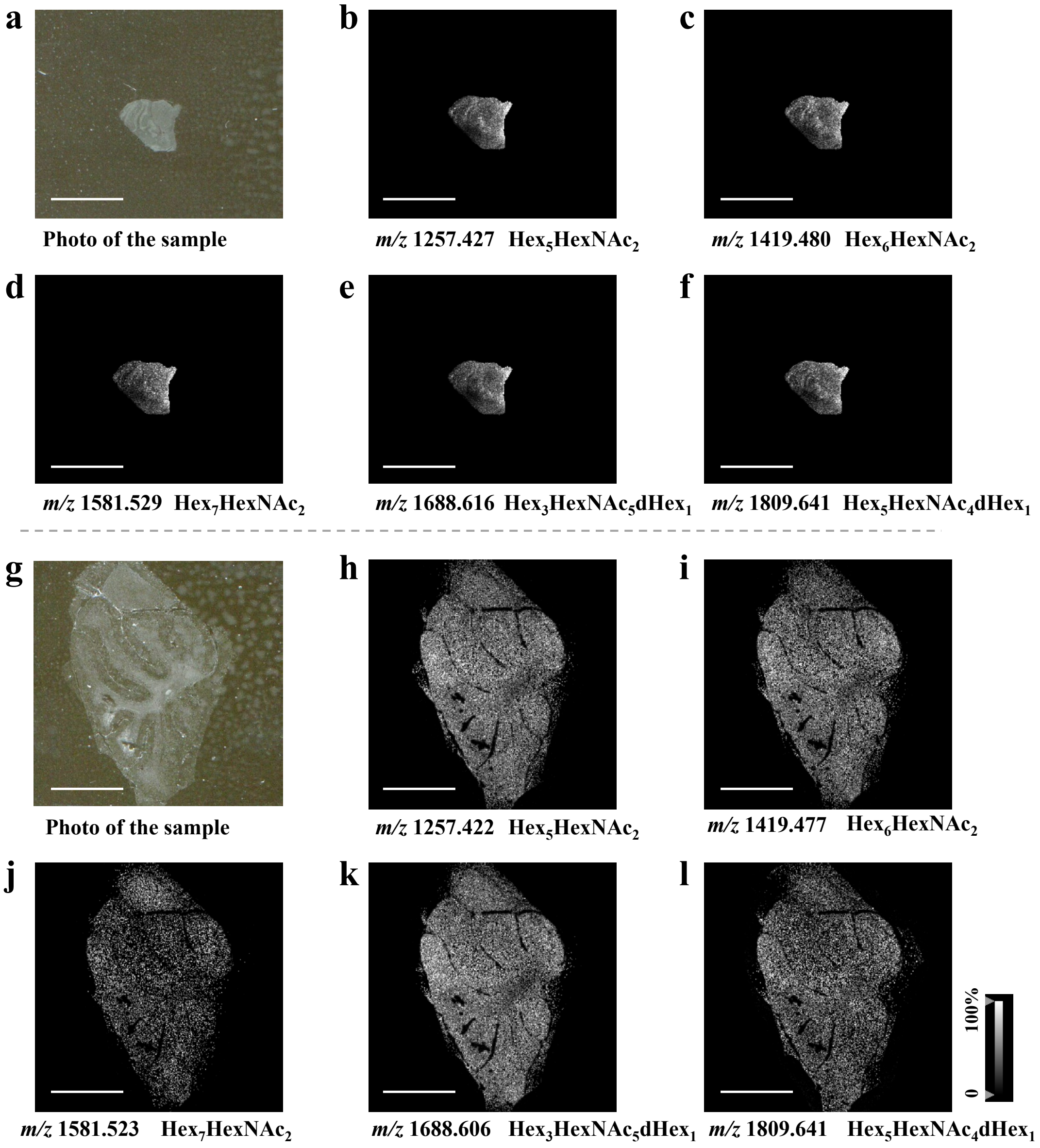

Supplementary Figure 18. Comparative spatial mapping of N-glycans from the mouse cerebellum between unexpanded control and TEMI. a. Bright field photo image of an unexpanded cerebellum tissue section before MALDI matrix deposition, b-f. N-glycans from the unexpanded cerebellum tissue section, g. Bright field photo image of an ~3.5-fold linearly expanded cerebellum tissue section before MALDI matrix deposition, h-l. TEMI of N-glycans from the cerebellum. The scale bar is 4 mm. The experiments were carried out using a 50 µm laser raster scanning and the MSI images obtained with mass error tolerance of 10 ppm. Hex, Hexose (Galactose, Mannose); HexNAc, N-Acetylhexosamine; NeuAc, N-Acetyl neuraminic acid; dHex, Fucose.

Supplementary Table 1. List of the identified lipid species from mouse brain samples using TEMI under positive mode

Name	Type	Molecular Formula	Experimental *m/z*	Theoretical adduct *m/z*[a]	Error[b] Δ *m/z* [ppm]	Enriched in cerebellum layer[c]
PE 28:1;O	$[M+H]^+$	C33H64NO9P	650.444	650.439	6.872	WML
PE 30:1;O	$[M+H]^+$	C35H68NO9P	678.476	678.470	8.696	WML
PC 30:0	$[M+H]^+$	C38H76NO8P	706.543	706.538	7.416	WML
PS 31:3	$[M+H]^+$	C37H66NO10P	716.454	716.450	6.504	WML, GrCL
PS 31:2	$[M+H]^+$	C40H80NO7P	718.467	718.465	1.990	WML, GrCL
PC 28:3;O3	$[M+H]^+$	C40H82NO7P	720.442	720.445	-3.984	GrCL, ML
PS 31:0	$[M+H]^+$	C37H72NO10P	722.502	722.497	6.699	GrCL
PC 32:3	$[M+H]^+$	C40H74NO8P	728.519	728.519	0.000	WML
PC 32:1	$[M+H]^+$	C40H78NO8P	732.559	732.554	7.754	WML
PC 32:0	$[M+H]^+$	C40H80NO8P	734.568	734.569	-1.361	ML
PC O-34:2	$[M+H]^+$	C42H82NO7P	744.594	744.590	5.547	WML
PE 36:1	$[M+H]^+$	C41H80NO8P	746.574	746.569	5.961	WML
PC O-34:1	$[M+H]^+$	C42H84NO7P	746.611	746.606	7.206	WML
PC 30:3;O3	$[M+H]^+$	C38H70NO11P	748.473	748.476	-4.462	GrCL
PS O-34:0	$[M+H]^+$	C40H80NO9P	750.571	750.564	9.220	ML
PC 34:4	$[M+H]^+$	C42H76NO8P	754.541	754.538	3.896	WML
PC 34:3	$[M+H]^+$	C42H78NO8P	756.558	756.554	5.631	ML
PC 34:2	$[M+H]^+$	C42H80NO8P	758.573	758.569	5.787	ML, GrCL
PC 34:1	$[M+H]^+$	C42H82NO8P	760.587	760.585	2.261	WML
PC 34:0	$[M+H]^+$	C42H84NO8P	762.605	762.601	4.590	ML
PE 38:4	$[M+H]^+$	C43H78NO8P	768.557	768.554	3.982	WML
PC O-36:4	$[M+H]^+$	C44H82NO7P	768.595	768.590	6.883	WML
PS O-36:4	$[M+H]^+$	C42H76NO9P	770.535	770.533	2.064	ML
PC O-36:2	$[M+H]^+$	C44H86NO7P	772.626	772.621	5.915	ML
PC 35:1	$[M+H]^+$	C43H84NO8P	774.606	774.601	6.494	ML
PC 36:5	$[M+H]^+$	C44H78NO8P	780.558	780.554	5.624	WML
PC 36:4	$[M+H]^+$	C44H80NO8P	782.575	782.569	7.450	WML
PC 36:3	$[M+H]^+$	C44H82NO8P	784.585	784.585	-0.370	ML, GrCL
PC 36:2	$[M+H]^+$	C44H84NO8P	786.608	786.601	8.416	WML
PC 36:1	$[M+H]^+$	C44H86NO8P	788.620	788.616	5.034	WML
PC 38:6	$[M+H]^+$	C46H80NO8P	806.573	806.569	5.096	GrCL
PC 38:5	$[M+H]^+$	C46H82NO8P	808.588	808.585	3.611	WML
PC 38:4	$[M+H]^+$	C46H84NO8P	810.604	810.601	3.602	WML
PC 38:3	$[M+H]^+$	C46H86NO8P	812.613	812.616	-4.615	WML
PC 38:1	$[M+H]^+$	C46H90NO8P	816.653	816.650	3.943	WML

PC 40:10	$[M+H]^+$	C48H76NO8P	826.541	826.538	3.533	ML
IPC 36:0;O3	$[M+H]^+$	C42H84NO12P	826.578	826.580	-2.649	WML
PC 40:9	$[M+H]^+$	C48H78NO8P	828.556	828.554	2.776	GrCL
PC 40:8	$[M+H]^+$	C48H80NO8P	830.568	830.569	-1.950	GrCL
PC 40:7	$[M+H]^+$	C48H82NO8P	832.589	832.585	4.228	ML, GrCL
PE 42:0	$[M+H]^+$	C47H94NO8P	832.672	832.679	-8.671	GrCL
PC 40:6	$[M+H]^+$	C48H84NO8P	834.606	834.601	5.775	ML
PC 40:5	$[M+H]^+$	C48H86NO8P	836.620	836.616	4.363	ML, WML
PC 40:4	$[M+H]^+$	C48H88NO8P	838.634	838.632	2.552	WML
PS 40:4	$[M+H]^+$	C46H82NO10P	840.578	840.575	3.486	GrCL
PC 40:3	$[M+H]^+$	C48H90NO8P	840.645	840.648	-3.485	WML
PS 40:3	$[M+H]^+$	C46H84NO10P	842.593	842.591	3.359	ML, GrCL, WML
PC 40:2	$[M+H]^+$	C48H92NO8P	842.669	842.663	7.073	WML
PC 40:1	$[M+H]^+$	C48H94NO8P	844.686	844.679	7.873	WML
PC 40:0	$[M+H]^+$	C48H96NO8P	846.690	846.695	-5.516	WML
PS O-42:6	$[M+H]^+$	C48H84NO9P	850.602	850.596	7.736	ML
PC O-42:4	$[M+H]^+$	C50H94NO7P	852.689	852.684	5.406	WML
PC 42:9	$[M+H]^+$	C50H82NO8P	856.586	856.585	0.876	ML, GrCL, WML
PC 42:8	$[M+H]^+$	C50H84NO8P	858.593	858.601	-9.422	ML, GrCL, WML
PE 44:1	$[M+H]^+$	C49H96NO8P	858.700	858.695	6.219	WML
PC 42:7	$[M+H]^+$	C50H86NO8P	860.617	860.616	0.662	WML
PC 42:5	$[M+H]^+$	C50H90NO8P	864.650	864.648	2.059	WML
PC 42:4	$[M+H]^+$	C50H92NO8P	866.667	866.663	4.338	WML
PS 42:4	$[M+H]^+$	C48H86NO10P	868.610	868.606	4.686	ML
PC 42:2	$[M+H]^+$	C50H96NO8P	870.702	870.695	8.258	WML
PC 42:1	$[M+H]^+$	C50H98NO8P	872.715	872.710	5.890	WML
PS 44:12	$[M+H]^+$	C50H74NO10P	880.517	880.512	5.644	ML
PE 46:4	$[M+H]^+$	C51H94NO8P	880.685	880.679	6.336	WML
SHexCer 42:2;O2	$[M+H]^+$	C48H91NO11S	890.644	890.639	5.816	ML
SHexCer 42:1;O2	$[M+H]^+$	C48H93NO11S	892.650	892.654	-4.727	ML
PC 44:5	$[M+H]^+$	C52H94NO8P	892.681	892.679	2.498	WML
PC 44:4	$[M+H]^+$	C52H96NO8P	894.696	894.695	2.112	WML
PC 44:2	$[M+H]^+$	C52H100NO8P	898.732	898.726	6.442	WML
PC 44:1	$[M+H]^+$	C52H102NO8P	900.747	900.742	6.128	WML

[a]Theoretical adduct *m/z* refers to databases of Human Metabolome Database (http//: www.hmdb.ca) and LIPID MAPS (http//: www. lipidmaps.org)

[b]Error = ((experimental adduct mass - theoretical adduct mass)/ theoretical adduct mass)*1000000

[c]Enriched in cerebellum layers (Molecular layer (ML), granular cell layer (GrCL), and white matter layer (WML)): estimated dominant distribution of the corresponding lipid.

Supplementary Table 2. List of the identified lipid species from mouse brain samples using TEMI under negative mode

Name	Type	Molecular Formula	Experimental m/z	Theoretical adduct m/z[a]	Error[b] Δ m/z [ppm]	Enriched in cerebellum layers[c]
LPA 16:0	$[M-H]^-$	C19H39O7P	409.236	409.236	-0.635	ML
LPA 18:1	$[M-H]^-$	C21H41O7P	435.254	435.252	4.733	WML
LPA 18:0	$[M-H]^-$	C21H43O7P	437.269	437.267	3.408	ML
LPE 16:0	$[M-H]^-$	C21H44NO7P	452.281	452.278	6.832	WML
LPA 20:1	$[M-H]^-$	C23H45O7P	463.284	463.283	2.698	WML
LPE 18:1	$[M-H]^-$	C23H46NO7P	478.296	478.294	4.621	WML
LPE 18:0	$[M-H]^-$	C23H48NO7P	480.311	480.310	3.456	ML, GrCL
LPE 20:4	$[M-H]^-$	C25H44NO7P	500.280	500.278	3.178	WML
LPE 20:1	$[M-H]^-$	C25H50NO7P	506.327	506.325	4.483	WML
LPE 20:0	$[M-H]^-$	C25H52NO7P	508.342	508.341	1.436	WML
LPE 22:6	$[M-H]^-$	C27H44NO7P	524.281	524.278	4.711	WML
LPE 22:4	$[M-H]^-$	C27H48NO7P	528.312	528.310	3.710	WML
CerP 36:1;O2	$[M-H]^-$	C36H72NO6P	644.505	644.503	3.507	ML
PA 32:0	$[M-H]^-$	C35H69O8P	647.468	647.466	3.923	ML
PS 26:0	$[M-H]^-$	C32H62NO10P	650.406	650.404	3.183	WML
PS O-28:1	$[M-H]^-$	C34H66NO9P	662.443	662.440	3.729	WML
PS 27:0	$[M-H]^-$	C33H64NO10P	664.423	664.420	4.560	WML, GrCL, ML
PA 34:1	$[M-H]^-$	C37H71O8P	673.485	673.481	4.677	WML
PA 34:0	$[M-H]^-$	C37H73O8P	675.499	675.497	3.035	ML
PS 28:0	$[M-H]^-$	C34H66NO10P	678.437	678.435	2.506	WML
PS 29:0	$[M-H]^-$	C35H68NO10P	692.454	692.451	4.159	WML
PA 36:2	$[M-H]^-$	C39H73O8P	699.500	699.497	4.232	WML
PA 36:1	$[M-H]^-$	C39H75O8P	701.516	701.513	4.333	WML
CerPE 38:1;O2	$[M-H]^-$	C40H81N2O6P	715.578	715.576	3.396	ML
PE 34:1	$[M-H]^-$	C39H76NO8P	716.527	716.524	4.047	WML
PE 34:0	$[M-H]^-$	C39H78NO8P	718.542	718.539	3.451	ML
PA 38:4	$[M-H]^-$	C41H73O8P	723.499	723.497	2.184	WML
CerP 42:2;O2	$[M-H]^-$	C42H82NO6P	726.585	726.581	5.753	WML
PE O-36:2	$[M-H]^-$	C41H80NO7P	728.563	728.560	3.706	WML
PA 38:1	$[M-H]^-$	C41H79O8P	729.549	729.544	6.675	WML
CerPE 38:2;O3	$[M-H]^-$	C40H79N2O7P	729.561	729.555	8.334	WML
HexCer 34:1;O4	$[M-H]^-$	C40H77NO10	730.547	730.548	-0.684	WML
PE 36:2	$[M-H]^-$	C41H78NO8P	742.542	742.539	4.417	WML

HexCer 36:1;O3	[M-H]-	C42H81NO9	742.586	742.584	2.680	WML
PE 36:1	[M-H]-	C41H80NO8P	744.558	744.555	3.855	WML
PE 36:0	[M-H]-	C41H82NO8P	746.571	746.571	1.179	ML
PA 40:6	[M-H]-	C43H73O8P	747.500	747.497	4.134	ML
PS 33:0	[M-H]-	C39H76NO10P	748.507	748.513	-8.764	ML
PE O-38:3	[M-H]-	C43H82NO7P	754.578	754.576	3.048	WML
PE 38:4	[M-H]-	C43H78NO8P	766.542	766.539	3.261	WML
PE 38:2	[M-H]-	C43H82NO8P	770.574	770.571	4.075	WML
HexCer 38:1;O3	[M-H]-	C44H85NO9	770.617	770.615	1.856	WML
PE 38:1	[M-H]-	C43H84NO8P	772.589	772.586	3.663	WML
PG 37:6	[M-H]-	C43H73O10P	779.488	779.487	2.040	WML, GrCL
PS 36:2	[M-H]-	C42H78NO10P	786.532	786.529	3.725	ML
PS 36:1	[M-H]-	C42H80NO10P	788.548	788.545	3.602	WML
PE 40:6	[M-H]-	C45H78NO8P	790.542	790.539	4.124	ML
PI O-32:0	[M-H]-	C41H81O12P	795.532	795.539	-9.629	WML
HexCer 40:1;O3	[M-H]-	C46H89NO9	798.650	798.647	4.558	WML
PS 36:0;O	[M-H]-	C42H82NO11P	806.548	806.555	-8.716	WML
PG O-40:6	[M-H]-	C46H81O9P	807.554	807.555	-0.421	WML
HexCer 42:2;O2	[M-H]-	C48H91NO8	808.670	808.667	3.574	WML
TG 49:5	[M-H]-	C52H90O6	809.674	809.667	9.522	WML
PS 38:4	[M-H]-	C44H78NO10P	810.529	810.529	-0.407	WML
PE O-42:1	[M-H]-	C47H94NO7P	814.674	814.670	5.880	WML
PS 38:1	[M-H]-	C44H84NO10P	816.578	816.576	2.608	WML
PE 42:6	[M-H]-	C47H82NO8P	818.571	818.571	0.098	ML
PI O-34:1	[M-H]-	C43H83O12P	821.548	821.555	-8.666	WML
SHexCer 36:1;O3	[M-H]-	C42H81NO12S	822.545	822.541	5.605	WML
PG 40:5	[M-H]-	C46H81O10P	823.556	823.550	8.293	WML
HexCer 36:1;O	[M-H]-	C48H91NO9	824.665	824.662	3.092	WML
HexCer 42:1;O3	[M-H]-	C48H93NO9	826.680	826.678	2.710	WML
PS 40:6	[M-H]-	C46H78NO10P	834.531	834.529	2.073	ML
SHexCer 38:1;O2	[M-H]-	C44H85NO11S	834.579	834.577	2.432	WML
PI 34:1	[M-H]-	C43H81O13P	835.533	835.534	-1.508	WML
PG O-42:6	[M-H]-	C48H85O9P	835.584	835.586	-2.729	WML
PS 40:5	[M-H]-	C46H80NO10P	836.539	836.545	-6.587	WML
PS 40:4	[M-H]-	C46H82NO10P	838.558	838.560	-3.268	WML
SHexCer 38:1;O3	[M-H]-	C44H85NO12S	850.576	850.572	4.573	WML
PS 42:9	[M-H]-	C48H76NO10P	856.515	856.513	1.354	ML
PI 36:4	[M-H]-	C45H79O13P	857.519	857.519	0.431	WML

PS 42:8	[M-H]-	C48H78NO10P	858.523	858.529	-7.268	WML
IPC 40:1;O2	[M-H]-	C46H90NO11P	862.611	862.618	-8.497	WML
IPC 40:0;O2	[M-H]-	C46H92NO11P	864.626	864.634	-9.010	WML
SHexCer 40:1;O3	[M-H]-	C46H89NO12S	878.606	878.603	3.119	WML
PG 44:5	[M-H]-	C50H89O10P	879.611	879.612	-1.648	WML
IPC 40:0;O3	[M-H]-	C46H92NO12P	880.621	880.628	-8.925	WML
PG 44:4	[M-H]-	C50H91O10P	881.622	881.628	-6.590	WML
PI 38:5	[M-H]-	C47H81O13P	883.535	883.534	0.555	WML
PI 38:4	[M-H]-	C47H83O13P	885.552	885.550	2.417	WML
PS 44:8	[M-H]-	C50H82NO10P	886.557	886.560	-3.373	WML
PI 38:3	[M-H]-	C47H85O13P	887.562	887.566	-4.067	WML
SHexCer 42:2;O2	[M-H]-	C48H91NO11S	888.626	888.624	2.723	WML
SHexCer 42:1;O2	[M-H]-	C48H93NO11S	890.640	890.640	0.404	WML
TG 56:13	[M-H]-	C59H88O6	891.645	891.651	-6.325	WML
PI O-40:2	[M-H]-	C49H93O12P	903.642	903.633	9.705	WML
SHexCer 42:2;O3	[M-H]-	C48H91NO12S	904.621	904.619	2.454	WML
SHexCer 42:1;O3	[M-H]-	C48H93NO12S	906.636	906.635	1.831	WML

[a]Theoretical adduct m/z refers to databases of Human Metabolome Database (http//: www.hmdb.ca) and LIPID MAPS (http//: www. lipidmaps.org).

[b]Error = ((experimental adduct mass - theoretical adduct mass)/ theoretical adduct mass)*1000000.

[c]Enriched in cerebellum layers (Molecular layer (ML), granular cell layer (GrCL), and white matter layer (WML)): estimated dominant distribution of the corresponding lipid.

Supplementary Table 3. Details of detected lipid species in Purkinje neuron cells using TEMI under positive mode

Name	Type	Molecular Formula	Experimental *m/z*	Theoretical adduct *m/z*[a]	Error[b] Δ *m/z* [ppm]	Enriched in Purkinje neuron cells
PC 32:0	$[M+H]^+$	C40H80NO8P	734.575	734.569	7.217	No
PC 34:1	$[M+H]^+$	C42H82NO8P	760.585	760.588	-3.57	No
PC 36:1	$[M+H]^+$	C44H87NO8P	788.614	788.616	2.54	Yes
PC 36:0	$[M+H]^+$	C44H89NO8P	790.631	790.632	1.26	Yes
PC 38:4	$[M+H]^+$	C46H85NO8P	810.601	810.601	0.74	Yes
PC 38:3	$[M+H]^+$	C46H87NO8P	812.614	812.616	2.83	Yes
PC 38:2	$[M+H]^+$	C46H89NO8P	814.636	814.632	4.79	Yes
PC 38:1	$[M+H]^+$	C46H91NO8P	816.647	816.648	1.47	Yes
PC 40:6	[M+H]+	C48H85NO8P	834.603	834.601	2.76	Yes
PC 40:5	[M+H]+	C48H87NO8P	836.617	836.616	0.48	Yes
PC 40:4	[M+H]+	C48H89NO8P	838.636	838.632	4.17	Yes
PC 42:9	[M+H]+	C50H83NO8P	856.583	856.585	2.57	Yes

[a]Theoretical adduct m/z refers to databases of Human Metabolome Database (http//: www.hmdb.ca) and LIPID MAPS (http//: www. lipidmaps.org).

[b]Error = ((experimental adduct mass - theoretical adduct mass)/ theoretical adduct mass)*1000000.

Supplementary Table 4. List of the identified on-tissue tryptic peptides matched with LC-MS proteomics results from mouse cerebella using TEMI under positive mode.

Experimental adduct mass, *m/z* $[M + H]^+$	Theoretical adduct mass, *m/z* $[M + H]^+$	Mass Error (ppm) *	Putative Peptides	Modification	Putative Gene Name
709.3432	709.3416	-2.26	FSWGGR		Mbp
858.5155	858.5155	0.00	QLATKAAR		H3c1
872.5193	872.52	0.80	TGIVLANGK		Map1a
890.4391	890.4407	1.80	FGYQFTK		Tmod2
917.5064	917.505	-1.53	GTVGSILDR		Abce1
935.4981	935.4945	-3.85	DGYAQILR		Aco2
938.4828	938.4829	0.11	EAIDSYIK		Cltc
943.5454	943.5458	0.42	LTVPSADLK		Slc6a11
953.6042	953.6029	-1.36	LLLPGELAK		H2bc26
984.5395	984.536	-3.55	AVTKYTSSK		H2bc26
1006.52	1006.5203	0.40	ANEFLEVGK		Eif3a
1024.561	1024.5574	-3.32	FIRNFAEK		Atp5mg
1039.52	1039.5241	4.04	FGNMGSLSVK		Lama1
1049.5	1049.501	0.86	SHFESSTVR		Epb41l3
1067.505	1067.5004	-4.03	EYQLNDSAK		Gnao1
1087.59	1087.5928	3.03	SPQMVSAIVR		Ctnnb1
1115.596	1115.5942	-1.61	AVDEAADALLK		Immt
1179.633	1179.6368	3.14	VPQIEVETHK		Dync1h1
1203.572	1203.5739	1.91	VAVEEVDEEGK		Lmna
1215.674	1215.6731	-0.58	FKVPGVEASGPK		Ahnak
1225.616	1225.617	0.90	ASHLELNNGTK	1A(30.0105), 2S(12.0000)	Akr1b1
1229.679	1229.6848	4.39	ELIIGDRQTGK		Atp5f1a
1263.718	1263.7168	-1.19	LNIPVNQVNPR		Atp1a1
1288.694	1288.6929	-0.85	VVDTIDQLVMR		Sugp2
1303.735	1303.7368	1.30	DLTPEHLPLLR		Dcps
1311.575	1311.5699	-3.51	EDQTEYLEER		Hsp90aa1
1339.71	1339.7076	-1.94	HRDTGILDSIGR		Mbp
1347.739	1347.7379	-0.67	ALIAAQYSGAQVR		Eef1g
1358.7	1358.6964	-2.58	FAGHNFRNPSVL		Eno2
1363.764	1363.758	-4.03	ITVTSEVPFSKR		Rpl22
1367.772	1367.7753	2.27	AVDSLVPIGRGQR		Atp5f1a
1411.783	1411.7791	-2.55	LEEGPPVTTVLTR		Pdha1
1415.692	1415.6913	-0.14	TWGDAGAAAGGGIPSK		Prkar2b

1440.742	1440.7369	-3.33	VEEFLYEKLDR		Sh3glb2
1493.71	1493.7118	0.94	TGENVEDAFLEAAK		Rab14
1498.857	1498.8587	1.00	SGKAPILIATDVASR		Ddx17
1503.865	1503.8641	-0.33	NLLSVAYKNVVGAR		Ywhaz
1515.761	1515.7649	2.31	EQGVDENETLLLR		Tln2
1569.768	1569.7655	-1.53	KAEAGAGSATEFQFR		Rps10
1575.863	1575.8588	-2.41	SSLLLDTVTSIPSSR		Map1a
1589.744	1589.7441	0.31	HQEGEIFDTEKEK		Rpl6
1649.905	1649.9009	-2.42	VEFLRPSFIDGTIR		Nectin1
1680.863	1680.8564	-4.16	GLSLSRFSWGGRDSR		Mbp
1696.857	1696.8514	-3.18	TTHYGSLPQKSQHGR		Mbp
1707.871	1707.866	-2.93	SVHHALSDVQAEVSTK		Mmrn2
1714.913	1714.9122	-0.64	SSGPTSLFAVTVAPPGAR		Hnrnpu
1719.034	1719.0288	-3.08	AGLQFPVGRVHRLLR		H2ac15
1737.031	1737.0268	-2.59	EVQTAVRLLLPGELAK		H2bc26
1741.858	1741.8602	1.09	ELDALDANDELTPLGR		Dhx9
1751.045	1751.0435	-1.09	EVQTAVRLLLPGELAK	1E(14.0167)	H2bc26
1764.941	1764.9391	-1.30	AAALEFLNRFEEAKR		Stip1
1816.938	1816.934	-1.93	IQPNDGPVFFKVDGQR		Cnrip1
1903.954	1903.9508	-1.79	SAYSSYSAPVSSSLSVRR		Nefl
2063.064	2063.0596	-1.89	TWNDPSVQQDIKFLPFK		Hspa5
2232.075	2232.0785	1.39	QQAAMGSQGNLSAEVEQATRR		Atp6v1g2
2397.392	2397.385	-3.04	ETVSIEVKEVVKPLLVSTLGEK		Mecp2
2402.242	2402.2323	-4.16	HRDTGILDSIGRFFSGDRGAPK		Mbp

*Error = ((experimental adduct mass - theoretical adduct mass)/ theoretical adduct mass)*1000000.

Supplementary Table 5. N-glycans detected from mouse cerebellum using TEMI

Experimental mass [M + Na]$^+$ *m/z*	Identified N-glycans [a]	Theoretical adduct mass [b] [M + Na]$^+$ *m/z*	Mass Error (ppm) [c]
1257.4287	Hex5HexNAc2	1257.4230	4.53
1282.4520	Hex3HexNAc3dHex1	1282.4550	-2.34
1419.4773	Hex6HexNAc2	1419.4760	0.92
1444.5151	Hex4HexNAc3dHex1	1444.5080	4.92
1485.5330	Hex3HexNAc4dHex1	1485.5342	-0.83
1542.5526	Hex3HexNAc5	1542.5557	-2.03
1581.5233	Hex7HexNAc2	1581.5290	-3.60
1606.5572	Hex5HexNAc3dHex1	1606.5604	-2.01
1647.5897	Hex4HexNAc4dHex1	1647.5870	1.64
1663.5742	Hex5HexNAc4	1663.5820	-4.69
1688.6061	Hex3HexNAc5dHex1	1688.6136	-4.46
1743.5763	Hex8HexNAc2	1743.5820	-3.27
1768.6177	Hex6HexNAc3dHex1	1768.6132	2.52
1809.6406	Hex5HexNAc4dHex1	1809.6398	0.42
1825.6448	Hex6HexNAc4	1825.6350	5.37
1850.6709	Hex4HexNAc5dHex1	1850.6664	2.41
1905.6309	Hex9HexNAc2	1905.6340	-1.63
1955.6901	Hex5HexNAc4dHex2	1955.6977	-3.90
1971.7039	Hex6HexNAc4dHex1	1971.6926	5.71
1996.7244	Hex4HexNAc5dHex2	1996.7243	0.03
2012.7120	Hex5HexNAc5dHex1	2012.7190	-3.48
2053.7513	Hex9HexNAc2	2053.7458	2.66
2158.7793	Hex5HexNAc5dHex2	2158.7771	1.00
2174.7700	Hex6HexNAc5dHex1	2174.7720	-0.92
2304.8551	Hex5HexNAc5dHex3	2304.8350	8.71
2320.8251	Hex6HexNAc5dHex2	2320.8299	-2.08
2523.9184	Hex6HexNAc6dHex2	2523.9093	3.59
2539.9074	Hex7HexNAc6dHex1	2539.9042	1.25
2669.9707	Hex6HexNAc6dHex3	2669.9672	1.30

[a] Hex, hexose (galactose, mannose); HexNAc, N-acetylhexosamine; NeuAc, N-acetyl neuraminic acid; dHex, fucose

[b] Theoretical adduct *m/z* refers to databases of GlycoWorkbench (https://code.google.com/archive/p/glycoworkbench/)

[c] Error = ((experimental mass − theoretical mass)/ theoretical mass)*1000000

Supplementary Table 6. The expansion factor and the maximum measurement error of different tissue types under 1st, 2nd, and 3rd embeddings.

		pre-expansion	1st embedding	2nd embedding	3rd embedding
Cerebellum	expansion factor	1	1.7	2.4	3.5
	maximum deformation error	0	9.7%	5.6%	5.4%
Kidney	expansion factor	1	1.3	2.1	3
	maximum deformation error	0	9.5%	11.6%	11.9%
Pancreas	expansion factor	1	1.4	2.5	3.5
	maximum deformation error	0	9.7%	6.9%	8.3%

Supplementary Table 7. The LC-MS gradient used for the lipidomic analysis*

Time	Flow rate (mL/min)	%B
0	0.15	30
3.5	0.15	43
3.64	0.15	55
20.8	0.15	65
31.2	0.15	85
35	0.15	100
43	0.15	100
43.5	0.15	30
52	0.15	30

*: The mobile phase A: 60:40 (v:v) acetonitrile/water with 10 mM ammonium formate and 0.1% formic acid, the mobile phase B: 90:10 (v:v) IPA/acetonitrile with 10 mM ammonium formate and 0.1% formic acid. Column: Accucore™ Vanquish™ C18+ UHPLC Column (2.1 x 150 mm, 1.5 µm). Temperature: 55 °C. Flow rate: 150 µL/min.

Supplementary Table 8. The significantly changed lipids from the lipidomic analysis*

LipidMolec	Adjusted p-value	Significant pairs
PC(18:1_22:6)	0.0135	Ex_ExP;ctrl_ExP
PC(18:1_20:4)	0.0422	Ex_ExP;ctrl_ExP
PE(16:0_20:4)	0.0349	Ex_ExP;ctrl_ExP
PE(P-18:0_22:6)	0.0314	ctrl_ExP
DG(O-20:6_18:0)	0.0234	Ex_ExP;ctrl_ExP
PE(18:0_20:4)	0.0349	ctrl_ExP
PC(22:6_22:6)	0.0322	Ex_ExP;ctrl_ExP

PC(44:12)	0.0104	Ex_ExP;ctrl_ExP
LPE(20:1)	0.0373	Ex_ExP;ctrl_ExP
LPE(22:6)	0.0350	Ex_ExP;ctrl_ExP
PE(22:6_18:0)	0.0349	Ex_ExP;ctrl_ExP
PC(40:8)	0.0110	Ex_ExP;ctrl_ExP
LPC(22:6)	0.0349	ctrl_ExP;Ex_ExP
PA(24:5_18:0)	0.0367	ctrl_ExP
PS(18:0_22:6)	0.0079	Ex_ExP;ctrl_ExP
PC(42:10)	0.0079	Ex_ExP;ctrl_ExP
PC(42:8)	0.0260	Ex_ExP;ctrl_ExP
PC(16:0_20:5)	0.0322	Ex_ExP;ctrl_ExP
PE(19:0_15:0)	0.0441	ctrl_ExP
DG(18:0_20:4)	0.0190	Ex_ExP;ctrl_ExP
LPE(20:4)	0.0256	ctrl_ExP;Ex_ExP
PC(16:1_22:6)	0.0073	Ex_ExP;ctrl_ExP
PC(20:4_22:6)	0.0104	Ex_ExP;ctrl_ExP
PE(22:6_22:6)	0.0078	Ex_ExP;ctrl_ExP
PC(22:4_22:6)	0.0260	Ex_ExP;ctrl_ExP
DG(O-20:6_18:1)	0.0484	ctrl_ExP;Ex_ExP
DG(16:0_22:6)	0.0292	Ex_ExP;ctrl_ExP
DG(O-20:6_16:0)	0.0233	ctrl_ExP;Ex_ExP
PC(O-18:0_22:6)	0.0474	ctrl_ExP
PC(23:6_18:1)	0.0322	Ex_ExP;ctrl_ExP
PC(18:0_24:6)	0.0107	Ex_ExP;ctrl_ExP
PC(16:1_20:4)	0.0320	Ex_ExP;ctrl_ExP
PI(20:4_18:1)	0.0349	Ex_ExP;ctrl_ExP
PC(34:3)	0.0349	Ex_ExP;ctrl_ExP
PS(22:4_22:6)	0.0079	Ex_ExP;ctrl_ExP
PA(19:1_22:6)	0.0115	Ex_ExP;ctrl_ExP
DG(O-22:6_22:6)	0.0373	ctrl_ExP
PC(44:5)	0.0285	Ex_ExP;ctrl_ExP
PE(44:11)	0.0237	Ex_ExP;ctrl_ExP
PC(14:0_20:4)	0.0178	Ex_ExP;ctrl_ExP
PC(20:3_22:6)	0.0322	ctrl_ExP;Ex_ExP
PA(P-19:4_21:1)	0.0424	ctrl_ExP
PE(20:4_22:6)	0.0174	Ex_ExP;ctrl_ExP
PG(O-17:2_23:6)	0.0104	Ex_ExP;ctrl_ExP
PC(35:4)	0.0349	Ex_ExP;ctrl_ExP
AcCa(16:1)	0.0046	Ex_ExP;ctrl_ExP
PE(P-17:0_20:4)	0.0387	ctrl_ExP
LPE(16:1)	0.0256	Ex_ExP;ctrl_ExP
LPE(24:4)	0.0413	Ex_ExP;ctrl_ExP
DG(18:1_22:6)	0.0256	Ex_ExP;ctrl_ExP
PC(44:7)	0.0349	Ex_ExP;ctrl_ExP
PC(O-18:4_22:1)	0.0473	ctrl_ExP;Ex_ExP
AcCa(18:2)	0.0182	Ex_ExP;ctrl_ExP

AcCa(20:4)	0.0260	Ex_ExP;ctrl_ExP
PA(21:2_22:6)	0.0320	ctrl_ExP;Ex_ExP
PA(25:7_18:0)	0.0292	ctrl_ExP;ctrl_Ex
LPE(24:2)	0.0256	Ex_ExP;ctrl_ExP
PS(24:0_18:1)	0.0484	ctrl_ExP
PI(20:4_16:0)	0.0486	ctrl_ExP;Ex_ExP
PC(O-17:2_22:6)	0.0276	Ex_ExP;ctrl_ExP
LPE(24:3)	0.0398	Ex_ExP;ctrl_ExP
PE(O-18:1_22:6)	0.0078	Ex_ExP;ctrl_ExP
PS(20:1_22:6)	0.0473	Ex_ExP;ctrl_ExP
PI(22:6_16:0)	0.0474	Ex_ExP;ctrl_ExP
PS(19:3_18:1)	0.0191	Ex_ExP;ctrl_ExP
PE(24:6_22:6)	0.0322	Ex_ExP;ctrl_ExP
PE(O-18:2_20:4)	0.0181	Ex_ExP;ctrl_ExP
PA(18:0_22:6)	0.0187	ctrl_ExP;Ex_ExP
LPE(24:6)	0.0329	Ex_ExP;ctrl_ExP
PE(O-18:1_20:4)	0.0237	Ex_ExP;ctrl_ExP
PC(O-16:1_20:4)	0.0256	Ex_ExP;ctrl_ExP
PE(18:1_18:1)	0.0203	Ex_ExP;ctrl_ExP
LPC(24:2)	0.0182	Ex_ExP;ctrl_ExP
PS(21:5_18:0)	0.0255	Ex_ExP;ctrl_ExP
PI(18:1_20:4)	0.0165	ctrl_ExP;Ex_ExP
PC(40:9)	0.0191	ctrl_ExP;Ex_ExP
PE(16:1_18:2)	0.0205	Ex_ExP;ctrl_ExP
PE(37:6)	0.0104	Ex_ExP;ctrl_ExP
PE(O-18:2_22:6)	0.0237	Ex_ExP;ctrl_ExP
PA(22:4_25:5)	0.0243	Ex_ExP;ctrl_ExP
PS(19:4_18:1)	0.0320	ctrl_ExP;Ex_ExP
PC(16:0_20:3)	0.0441	ctrl_ExP;Ex_ExP
PE(24:6_18:0)	0.0084	Ex_ExP;ctrl_ExP
PA(P-19:4_21:2)	0.0398	Ex_ExP;ctrl_ExP
PS(18:0_22:4)	0.0095	Ex_ExP;ctrl_ExP
PA(23:4_18:0)	0.0037	Ex_ExP;ctrl_ExP
PE(22:6_14:0)	0.0079	Ex_ExP;ctrl_ExP
TG(16:0_18:0_18:1)	0.0285	ctrl_ExP;Ex_ExP
PC(45:6)	0.0276	Ex_ExP;ctrl_ExP
PE(O-16:0_18:1)	0.0349	ctrl_ExP
PA(18:0_20:4)	0.0203	Ex_ExP;ctrl_ExP
PA(18:1_18:1)	0.0152	ctrl_ExP;Ex_ExP
PI(40:8)	0.0037	Ex_ExP;ctrl_ExP
DG(P-22:7_22:6)	0.0365	ctrl_ExP;Ex_ExP
PS(16:0_18:1)	0.0214	ctrl_ExP;Ex_ExP
PA(22:6_28:5)	0.0349	Ex_ExP;ctrl_ExP
PE(O-18:0_22:6)	0.0115	Ex_ExP;ctrl_ExP
PE(O-16:1_16:1)	0.0396	ctrl_ExP
PG(22:6_22:6)	0.0039	Ex_ExP;ctrl_ExP

PI(18:1_22:6)	0.0473	ctrl_ExP;Ex_ExP
TG(16:0_22:6_22:6)	0.0237	Ex_ExP;ctrl_ExP
PC(47:6)	0.0258	Ex_ExP;ctrl_ExP
PC(18:1_20:3)	0.0078	Ex_ExP;ctrl_ExP
PE(18:0_20:3)	0.0443	ctrl_ExP;Ex_ExP
PE(O-17:1_22:6)	0.0182	Ex_ExP;ctrl_ExP
PC(O-14:0_22:7)	0.0118	ctrl_ExP;Ex_ExP
PA(18:0_22:4)	0.0392	ctrl_ExP;Ex_ExP
PS(O-16:1_22:6)	0.0314	ctrl_ExP;Ex_ExP
PC(20:2_22:6)	0.0078	Ex_ExP;ctrl_ExP
PE(P-18:1_20:5)	0.0410	ctrl_ExP;Ex_ExP
PI(16:0_20:5)	0.0349	ctrl_ExP;Ex_ExP
PS(22:1_22:6)	0.0320	Ex_ExP;ctrl_ExP
PS(42:4)	0.0448	Ex_ExP;ctrl_ExP
PI(17:0_22:6)	0.0258	ctrl_ExP;Ex_ExP
PS(22:6_22:6)	0.0188	Ex_ExP;ctrl_ExP
PC(O-14:0_20:5)	0.0205	ctrl_ExP;Ex_ExP
PC(O-42:5)	0.0174	Ex_ExP;ctrl_ExP
PG(25:8_18:1)	0.0392	Ex_ExP;Ex_ctrl
PS(16:0_22:5)	0.0152	ctrl_ExP;Ex_ExP
PG(23:6_18:0)	0.0118	ExP_ctrl;Ex_ctrl
PG(18:0_25:6)	0.0256	Ex_ctrl;Ex_ExP
PC(18:1_18:2)	0.0104	Ex_ctrl;Ex_ExP
PC(O-46:8)	0.0020	ExP_ctrl;Ex_ctrl;Ex_ExP
Cer(d18:0_16:0)	0.0256	ExP_Ex;ExP_ctrl
PA(O-28:6_20:4)	0.0102	ExP_ctrl;Ex_ctrl;Ex_ExP

*: The ANOVA test was performed for significance analysis, followed by post hoc Tukey's HSD test. The Benjamini-Hochberg adjusted p value that less than or equal to 0.05 was considered as significance. Ex: expanded without applying proteinase K, ExP: expanded with applying proteinase K, ctrl: Control (without expansion).

Supplementary Table 9. The LC-MS gradient used for the metabolomics analysis*

Time	Flow rate (mL/min)	%B
0	0.2	100
8	0.2	100
30.8	0.2	70
38	0.2	40
41	0.2	30
51	0.2	100
68	0.2	100

*: The mobile phase A: water with 10mM ammonium formate and 0.125% formic acid. The mobile phase B: acetonitrile/water (95/5, v/v) with 10mM ammonium formate and 0.125% formic acid. Column: Waters Acquity UPLC BEH Amide column (150 mm× 2.1 mm, 1.7 μm). Temperature: 45 °C. Flow rate: 200 μL/min.

Supplementary Table 10. The LC-MS gradient used for the proteomics analysis*

Time	Flow rate (nL/min)	%B
0	200	2
7.4	200	8
88.8	200	27
118.4	200	42
119.4	200	99
124.95	200	99
126.3	200	99
130	200	50

*: The mobile phase A: water with 0.1% formic acid. The mobile phase B: acetonitrile/water (80/20, v/v) with 0.1% formic acid. Column: Aurora Ultimate XT 25×75 C18 UHPLC column (25 cm× 75 μm, 1.7 μm). Temperature: 35 °C. Flow rate: 200 nL/min.

Supplementary Note 1

Comparison among TEMI, GAMSI, and Ex-MSI

Sample preparation of GAMSI (middle) and Ex-MSI (right) were strictly following the literature of *Nat Commun* 15, 5036 (2024) and *CCS Chemistry* 6, 2662–2670 (2024), respectively. Overall, TEMI outperforms the other two methods—severe cracks (as indicated by the white arrows in a) were observed in the expanded tissue prepared by GAMSI and Ex-MSI methods. We found the tissue samples cracking in GAMSI and Ex-MSI was associated with the drying of the thick tissue-gel material on the ITO slide (a required step for MS imaging), as no cryosection was performed following tissue expansion in these two methods. In addition, chemical delocalization was found in both GAMSI and Ex-MSI, with more severe chemical diffusion observed in Ex-MSI. The chemical diffusion may be associated with proteolysis digestion of the tissue during expansion treatment, where GAMSI used a 2-day trypsin digestion in PBS buffer treatment, and Ex-MSI used 3-hour Proteinase K digestion at 60 °C for more extensive tissue digestion during sample preparation. It is worth mentioning that, since the GAMSI and Ex-MSI experiments strictly adhered to the published studies (refs. GAMSI, *Nat Commun 15, 5036 (2024)* and Ex-MSI, *CCS Chemistry 6, 2662–2670 (2024)*), the sample preparation strategies were different among the different methods. Certain important experimental conditions, such as thickness of the tissue slice (used for tissue expansion), MALDI matrix and laser energy, were different among different methods. Specifically, for MALDI matrix used in **Figure S5**, 9-AA matrix was used in GAMSI, NEDC matrix was used in Ex-MSI, and DAN matrix was used in TEMI for the control and corresponding expanded tissue samples; the laser energy was adjusted based on their MALDI matrix applied. For the thickness of the tissue slices, GAMSI used ~25 µm-thick mouse brain slices, while Ex-MSI used 50 µm-thick mouse brain slices for tissue expansion. Protease digestion was included in GAMSI and Ex-MSI, where GAMSI used a 2-day trypsin digestion in PBS buffer, and Ex-MSI used 3-hour Proteinase K digestion at 60 °C during sample preparation. Neither GAMSI nor Ex-MSI performed cryosection after tissue expansion; instead, the expanded tissue-hydrogel was directly dried on the ITO slide. In contrast, TEMI used a different strategy—a 300 μm-thick tissue slice was subjected to tissue expansion without any harsh denaturation (e.g., detergent treatment, protease digestion, or heating), and the expanded tissue-hydrogel was subjected to cryosection to obtain robust tissue sections for MS imaging. Thus, all these different conditions could contribute to the signal variations observed among the three methods.

Supplementary Note 2

Measurement of expansion non-uniformity.

Expansion non-uniformity is quantified as the deviation of point-to-point distance measurements from the expected values under the assumption of uniform expansion. For any pair of points in the expanded image, the measurement error is defined as the difference between the observed distance between the points and the distance that would have been expected if the expansion were uniform. These values are calculated using the code deposited at the end of this supplementary information. The expanded tissue image was registered to the pre-expansion tissue image using landmarks manually picked with BigWarp. The landmarks are exported as a csv file ("landmarks.csv"). The landmarks file is used by the Fiji script "createSimOrigAndThinPlateCoords.groovy" to generate three arrays of coordinates: one tiling the pre-expansion specimen space, one tiling the post-expansion specimen space assuming uniform expansion, and a third corresponding to the true nonrigid deformation of the expansion process, using a thin plate spline model. The MATLAB script "AllParts_measurement_error_analysis_createMask.m" compares the true coordinate array with the hypothetical uniform coordinate array to calculate the measurement errors for all pairs of points. These errors are then binned based on measurement length, and the mean and standard deviation of the measurements are presented as a function of measurement length shown in Figure S7. The Fiji script "bigwarpSimilarityPart.groovy" uses the "landmarks.csv" file to extract the average expansion factor for each specimen, which the script reports as the average scale of the non-rigid deformation.

Supplementary Note 3

Codes for Figure S7 and expansion factor

Fiji script: createSimOrigAndThinPlateCoords.groovy

```
import java.io.*
import java.nio.file.*
import java.util.*
import bigwarp.landmarks.*
import bigwarp.transforms.*
import net.imglib2.realtransform.*
import net.imglib2.realtransform.inverse.*

//USER: modify path to correspond to folder containing landmarks.csv for one pair of images
def homePath = "C:/Users..."
def exptFolderName = "/test"
def folderPath = homePath + exptFolderName

// Create coords_similarity, using the max and min x and y values of the landmarks as a bounding rectangle
// This confines the analyzed area to the minimum rectangular bounding box that contains all of the
menually picked landmarks
def landmarksFile = new File(folderPath, 'landmarks.csv')
def data = landmarksFile.readLines().collect { line ->
   def values = line.split(',')
   return values[2..-1].collect { it.toDouble() } // Skip the first two columns
}

def pointsFixed = data.collect { [it[2], it[3]] }
def pointsMoving = data.collect { [it[0], it[1]] }

def XMax = Math.ceil(pointsFixed.collect { it[0] }.max()).intValue()
def XMin = Math.floor(pointsFixed.collect { it[0] }.min()).intValue()
def YMax = Math.ceil(pointsFixed.collect { it[1] }.max()).intValue()
def YMin = Math.floor(pointsFixed.collect { it[1] }.min()).intValue()
def XRange = XMax - XMin
def YRange = YMax - YMin

def dXYstep = (int) Math.round(Math.sqrt(XRange * YRange) / 30)

def Xpoints = (XMin..(XMax + dXYstep)).step(dXYstep)
def Ypoints = (YMin..(YMax + dXYstep)).step(dXYstep)
def xyzSim = []
Xpoints.each { x ->
```

```
    Ypoints.each { y ->
        xyzSim << [x, y]
    }
}

// Write to CSV
def outputFile = new File(folderPath, 'coords_similarity.csv')
def writer = new FileWriter(outputFile)
xyzSim.each { coords ->
    writer.write(coords.join(',') + '\n')
}
writer.close()


def buildTransform(File landmarksPath, String transformType, int nd, boolean needInverse, double
invTolerance, int maxIters) {
    LandmarkTableModel ltm = new LandmarkTableModel(nd)
    try {
        ltm.load(landmarksPath)
    } catch (IOException e) {
        e.printStackTrace()
        return null
    }

        BigWarpTransform bwTransform = new BigWarpTransform(ltm, transformType)
    RealTransform xfm = bwTransform.getTransformation()

    if (xfm instanceof Wrapped2DTransformAs3D)
        xfm = ((Wrapped2DTransformAs3D) xfm).getTransform()

    if (needInverse) {
        if (transformType.equals("Thin Plate Spline")) {
            xfm.getOptimzer().setMaxIters(maxIters)
            xfm.getOptimzer().setTolerance(invTolerance)
        }
        xfm = xfm.inverse()
    }

        return xfm
}



def transformPoints(File landmarksPath, File inCsv, File outCsv, String inverseOrForward,
```

```
                String transformType, double invTolerance, int invMaxIters,
               boolean csvHasHeader, double sx, double sy, double sz) {


    boolean needInverseTransform = inverseOrForward.equals("Moving to target")

    // Read the input points
    List<String> lines
    try {
        lines = Files.readAllLines(Paths.get(inCsv.getAbsolutePath()))
    } catch (IOException e) {
        e.printStackTrace()
        return
    }

    // Get the transformation to apply
    int nd = lines.get(0).split(",").length
    RealTransform transform = buildTransform(landmarksPath, transformType, nd, needInverseTransform,
invTolerance, invMaxIters)

    // Transform all points
    List<String> outputLines = []
    double[] result = new double[nd]
    boolean firstLine = true
    for (String l : lines) {
        // Add the first line to the output if it's a header
        if (firstLine && csvHasHeader) {
            outputLines.add(l)
            firstLine = false
            continue
        }

        // Parse line
        double[] pt = Arrays.stream(l.split(",")).mapToDouble(Double::parseDouble).toArray()
        double[] scale = [sx, sy, sz] as double[]

        // Elementwise multiplication of pt and scale
        double[] scaledpt = new double[pt.length]
        for (int i = 0; i < pt.length; i++) {
            scaledpt[i] = pt[i] * scale[i]
        }

        // Transform point
        try {
```

```
            transform.apply(scaledpt, result)
        } catch (Exception e) {
            System.err.println("Warning: failed to transform " + Arrays.toString(scaledpt))
            Arrays.fill(result, Double.NaN)
        }

        outputLines.add(Arrays.toString(result).replaceAll("[\\[\\]]", "").replaceAll(" ", ""))
    }

    // Write output
    try {
        Files.write(Paths.get(outCsv.getAbsolutePath()), outputLines)
    } catch (IOException e) {
        e.printStackTrace()
    }
}
```

MATLAB script: AllParts_measurement_error_analysis_createMask.m

```
% this code was adapted from the code displaying deformation fields
% used in Jurriens, D., van Batenburg, V., Katrukha, E. A., & Kapitein, L. C. (2020). Mapping the neuronal cytoskeleton using expansion microscopy. In Methods in Cell Biology.
https://doi.org/10.1016/bs.mcb.2020.04.018

homeFolder = "C:\Users\...";
experimentFolderName = "\test";
experimentFolder = homeFolder + experimentFolderName;

runPart = 2;

% Instructions:
% 1) generate registration landmarks manually using BigWarp and save them
% as landmarks.csv
% 2) run createSimOrigAndThinPlateCoords.groovy, with the "folderPath"
% updated to correspond to the folder containing landmkars.csv
% 3) update "experimentFolder" above to correspond to the same folder and
% run this script to use "coords_thin_plate.csv" to calcualte the
% deformation field and measurement errors matrix.

% change runPart to 1  to manually choose analysis steps
% to run below if needed

previousDir = pwd;
```

```
cd(experimentFolder);


%% stages of analysis (1 = run, 0 = skip):

if runPart == 1 %manually set individual parts
   calculateDeformationField = 0;
   collectMeasurements =       0;
   measureErrors =             0;
elseif runPart == 2
   calculateDeformationField = 1;
   collectMeasurements =       1;
   measureErrors =             1;
end

%% settings:

% number of dimensions in the nonrigid transformation (2D vs 3D data).
% only 2D supported by this script
nD = 2;

%% size of expanded (target) image in scaled units (e.g. um)
detectSize = 1;

if detectSize
   landmarks=importdata('landmarks.csv');
   if size(landmarks.data,2) == 4
      nD = 2;
   elseif size(landmarks.data,2) == 6
      disp('error: detectSize not set up for 3D');
   else
      disp('error: landmarks.data should have 4 or 6 columns');
   end

   pointsFixed = landmarks.data(:,3:4);
   pointsMoving = landmarks.data(:,1:2);
   XMax = ceil(max(pointsFixed(:,1)));
   XMin = floor(min(pointsFixed(:,1)));
   YMax = ceil(max(pointsFixed(:,2)));
   YMin = floor(min(pointsFixed(:,2)));
   ZMax = 1;
   XRange = XMax - XMin;
   YRange = YMax - YMin;
```

```
else % manually set the size of the expanded image
    XMax=8178;
    YMax=7028;
    XMin=0;
    YMin=0;
    XRange = XMax - XMin;
    YRange = YMax - YMin;
    ZMax=1;
    if nD == 2; ZMax=1; end
end

%%
%image sampling: increase the step size to sample the image more sparsely
dXYstep = round(sqrt(XRange*YRange)/30);
dZstep = 1;

% create a mask using the convex hull containing all landmarks
bUseMask = 1;

%step size along meas length axis in final plot. This controls how much
%binning is done before displaying measurement error plot
%in sclaed units
dMeasStep = 10;


%% calculateDeformationField
% generate a vector field that indicates the nonrigid component of
% transformation from post-expansion image to ideal uniformly expanded
% coordiantes
% optional: export slices of the deformation field

if calculateDeformationField

    %number of tiff images in the mask sequence (# of Z planes)
    nFR=floor(ZMax / dZstep);
    % whether to export deformation field in XY as z-stack
    bExportXYdefField=true;

    % read coordinates from similarity array
    xySim=importdata('coords_similarity.csv');
    sz=size(xySim);

    boolmarks=zeros(sz(1),1);
```

```
if (bUseMask)
   %generate mask using convex hull of landmarks
   hullIndices = convhull(pointsFixed(:,1), pointsFixed(:,2));
   in_hull = inpolygon(xySim(:,1), xySim(:,2), pointsFixed(hullIndices,1), pointsFixed(hullIndices,2));
   boolmarks = double(in_hull);  % Convert logical to numeric
else
   boolmarks=boolmarks+1;
end


%  import transformed coordinates in thin plate
xySpline=importdata('coords_thin_plate.csv');

%vector difference between coordinates in 'similarity' and 'thin plate'
diffvect=xySim-xySpline;

% calculate absolute length of deformation vectors
totNFilt=sum(boolmarks);
displall=zeros(totNFilt,1);
displxy=zeros(totNFilt,1);
displz=zeros(totNFilt,1);
count=0;
for i=1:sz(1)
   if(boolmarks(i))
      count=count+1;
      displall(count)=norm(diffvect(i,:));
      displxy(count)=norm(diffvect(i,1:2));
      if nD == 3
         displz(count)=abs(diffvect(i,3));
      end
   end
end


%export deformation field in 2D (XY)
if (bExportXYdefField)
   h1=figure;
   if nD==3
      filt=(abs(xySim(:,3)-k)<0.00001);
      filt=filt&boolmarks;
   else
      filt = 1&boolmarks;
   end
```

```
        xy=xySpline(filt,1:2);
        xyvect=diffvect(filt,1:2);

        quiver(xy(:,1),xy(:,2),xyvect(:,1),xyvect(:,2),'AutoScale','off');

        axis equal
        set(gca,'Ydir','reverse');
        xlim([0 XMax]);
        ylim([0 YMax]);
        set(gcf,'Units','pixels');
        set(gcf,'Position',[20 20 600 500]);
        set(gca,'Units','pixels');
        set(gca,'Position',[20 20 550 550]);
        saveas(gcf,strcat('XY_sim_thinpl.png'));
    end

end


%% collectMeasurements
%for each pair of points in the image, find the correct distance between
%them assuming uniform expansion (similarity transformation). Find the
%'measured' distance between them (after nonrigid transformation). The
%difference between these lengths is the measurement error for that pair of
%points

if collectMeasurements
    boolmarksDec = boolmarks;

    nPointsDec = sum(boolmarksDec);

    xySimDec = NaN(nPointsDec,nD);
    diffvectDec = NaN(nPointsDec,nD);
    j = 1;

    for i = 1:length(boolmarks)
        if boolmarksDec(i)
            xySimDec(j,:) = xySim(i,:);
            diffvectDec(j,:) = diffvect(i,:);
            j = j + 1;
        end
    end
    if j ~= nPointsDec + 1
```

```
    disp('error: indices do not match!!');
  end


  nMeasDec = nPointsDec * (nPointsDec - 1);
  allMeasurements = NaN(nMeasDec,3);
  %col 1 is meas length
  %col 2 is meas length bin (i.e. which index in measLengths array)
  %col 3 is meas error
  %one row for every measurment (i.e. for every pair of points in the
  %masked region)

  measNum = 1;

  for i = 1:nPointsDec
    for j = 1:nPointsDec
      if i ~= j
        measLength = norm(xySimDec(j,:)-xySimDec(i,:));
        measError = norm(diffvectDec(j,:)-diffvectDec(i,:));
        allMeasurements(measNum,:) = [measLength NaN measError];
        measNum = measNum + 1;
      end
    end
  end
end

%% measureErrors
%bucket measurement errors by measurement length, and find the average and
%S.D. for each measurement length bucket

if measureErrors

  %compute bucket number for each length measurement
  allMeasurements(:,2) = round(allMeasurements(:,1)/dMeasStep + 0.5);

  maxMeas = max(allMeasurements(:,1));
  measLengths = dMeasStep/2:dMeasStep:ceil(maxMeas+dMeasStep);
  %measLength 0 only exists if dMeasStep>min pix spacing after decimation
  %in any direction

  measErrMtx = zeros(length(measLengths),4);
  measErrMtx(:,1) = measLengths;
  %each row is a measurement length bucket
  %for row n:
```

```
    %col 1: middle of bucket n (bucket 1 starts at measLength 0)
    %col 2: number of measurements in this bucket
    %col 3: average of measurement errors for this bucket
    %col 4: std dev of measuremnt errors for this bucket

    %add up number of measurments (col 2) and average error (col 3)for each bucket
    nMeasDec = size(allMeasurements,1);

    for i = 1:nMeasDec
       bucket = allMeasurements(i,2);
       measErrMtx(bucket,2) = measErrMtx(bucket,2) + 1;
       measErrMtx(bucket,3) = measErrMtx(bucket,3) + allMeasurements(i,3);
    end
    measErrMtx(:,3) = measErrMtx(:,3)./measErrMtx(:,2);%error is inf when N = 0

    %add up SD (col 4) for each bucket
    for i = 1:nMeasDec
       bucket = allMeasurements(i,2);
       %sum up squared difference between mesurement error and average error
       difference = allMeasurements(i,3)-measErrMtx(bucket,3);
       measErrMtx(bucket,4) = measErrMtx(bucket,4) + difference^2;
    end
    measErrMtx(:,4) = sqrt(measErrMtx(:,4)./measErrMtx(:,2));%div by N and take sqrt

    if nMeasDec ~= sum(measErrMtx(:,2))
       disp('Error: nMeasDec does not equal sum(measErrMtx(:,2)')
    end

    figure;
hold on;

% Filter out NaNs
iKeep = ~isnan(measErrMtx(:,3));

% Define the upper and lower bounds for the shaded area (mean ± S.D.)
upperBound = measErrMtx(iKeep,3) + measErrMtx(iKeep,4);
lowerBound = measErrMtx(iKeep,3) - measErrMtx(iKeep,4);
% Plot the mean line
plot(measErrMtx(iKeep,1), measErrMtx(iKeep,3), 'b', 'LineWidth', 1.5);
% Create the shaded area
xValues = measErrMtx(iKeep,1); % x-axis values
fill([xValues; flipud(xValues)], [upperBound; flipud(lowerBound)], 'b', 'FaceAlpha', 0.2, 'EdgeColor', 'none');

% Add labels and legend if needed
```

```
xlabel('Measurement Distance (µm)', 'FontName', 'Arial', 'FontSize', 12);
ylabel('Measurement Error (µm)', 'FontName', 'Arial', 'FontSize', 12);

legend('Mean', 'Standard Deviation');
set(gca, 'FontName', 'Arial', 'FontSize', 12);
hold off;

% Extract x-axis and mean y values
xValues = measErrMtx(iKeep, 1);       % x-axis values
meanValues = measErrMtx(iKeep, 3);    % mean values for y-axis

% Combine x and y mean values into a single matrix
meanData = [xValues, meanValues];

% Save to a CSV file
csvwrite('mean_points.csv', meanData);  % Saves to a file named 'mean_points.csv'


end

cd(previousDir)
```

Fiji script: bigwarpSimilarityPart.groovy

```
#@ File (label="Landmark file") landmarksPath
#@ Integer (label="Number of dimensions", value=2) nd
#@ String (label="Direction", choices={"Forward", "Inverse" }, value="Forward") direction

import mpicbg.models.*;
import bigwarp.landmarks.LandmarkTableModel;
import net.imglib2.util.*;

// load landmarks
tableModel = new LandmarkTableModel( nd );
try
{
    tableModel.load( landmarksPath );
} catch ( IOException e )
{
    e.printStackTrace();
    return;
}

def getModel( final int numDims )
{
```

```
    if( numDims == 2 )
        return new SimilarityModel2D();
    else if( numDims == 3 )
        return new SimilarityModel3D();
    else
        return null;
}


/**
 * Determinant of a 2d transform stored as a row-major 3d homogeneous matrix array
 */
def det2d( final double[][] mtx )
{
    return (mtx[0][0] * mtx[1][1]) - (mtx[0][1] * mtx[1][0]);
}

/**
 * Determinant of a 3d transform stored as a row major 4d homogeneous matrix array
 */
def det3d( final double[][] mtx )
{
    return  mtx[0][0] * mtx[1][1] * mtx[2][2] +
            mtx[1][0] * mtx[2][1] * mtx[0][2] +
            mtx[2][0] * mtx[0][1] * mtx[1][2] -
            mtx[0][2] * mtx[1][1] * mtx[2][0] -
            mtx[1][2] * mtx[2][1] * mtx[0][0] -
            mtx[2][2] * mtx[0][1] * mtx[1][0];
}

/**
 * Need this until Fiji's mpicbg versions are updated
 */
def double[][] toMatrix2d( final SimilarityModel2D model )
{
    a = new double[ 6 ];
    model.toArray( a );
    return [[ a[0], -a[1], a[4] ],
            [ a[1],  a[0], a[5] ]] as double[][];

}

/**
 * Need this until Fiji's mpicbg versions are updated
```

```
 */
def double[][] toMatrix3d( final SimilarityModel3D model )
{
	mtx = new double[ 3 ][ 4 ];
	model.toMatrix( mtx );
	return mtx;
}

model = getModel( nd );
if( model == null )
{
	println( "Only works for 2 or 3 dimensions");
	return;
}

// fit the model
int numActive = tableModel.numActive();
int ndims = tableModel.getNumdims();

mvgPts = new double[ ndims ][ numActive ];
tgtPts = new double[ ndims ][ numActive ];

tableModel.copyLandmarks( mvgPts, tgtPts );

double[] w = new double[ numActive ];
Arrays.fill( w, 1.0 );

try {
	model.fit( mvgPts, tgtPts, w );
} catch (NotEnoughDataPointsException e) {
	e.printStackTrace();
} catch (IllDefinedDataPointsException e) {
	e.printStackTrace();
}

themodel = model;
if( direction.equals( "Inverse" ) )
{
	themodel = model.createInverse();
	println( themodel )
}


if( nd == 2 )
```

```
{
      det = det2d( toMatrix2d( themodel ));
      avgscale = Math.sqrt( det );
}
else if( nd == 3 )
{
      det = det3d( toMatrix3d( themodel ));
      avgscale = Math.cbrt( det );
}

println( 'determinant : ' + det );
println( 'avgerage scale : ' + avgscale );
```
