# Supplementary material for: TEMI: tissue-expansion mass-spectrometry imaging
Source: Nat Methods. 2025 Apr 22;22(5):1051–8. doi: 10.1038/s41592-025-02664-9 (PMC12074994; doi:10.1038/s41592-025-02664-9)
Supplement: Supplementary file 2 — Reporting Summary [file 41592_2025_2664_MOESM2_ESM.pdf]

nature portfolio

Corresponding author(s): Lingjun Li
Paul W Tillberg
Meng Wang

Last updated by author(s): Jan 30, 2025

Reporting Summary

Nature Portfolio wishes to improve the reproducibility of the work that we publish. This form provides structure for consistency and transparency in reporting. For further information on Nature Portfolio policies, see our Editorial Policies and the Editorial Policy Checklist.

Statistics

For all statistical analyses, confirm that the following items are present in the figure legend, table legend, main text, or Methods section.

n/a	Confirmed	
☐	☒	The exact sample size (*n*) for each experimental group/condition, given as a discrete number and unit of measurement
☐	☒	A statement on whether measurements were taken from distinct samples or whether the same sample was measured repeatedly
☐	☒	The statistical test(s) used AND whether they are one- or two-sided<br>*Only common tests should be described solely by name; describe more complex techniques in the Methods section.*
☐	☒	A description of all covariates tested
☐	☒	A description of any assumptions or corrections, such as tests of normality and adjustment for multiple comparisons
☐	☒	A full description of the statistical parameters including central tendency (e.g. means) or other basic estimates (e.g. regression coefficient) AND variation (e.g. standard deviation) or associated estimates of uncertainty (e.g. confidence intervals)
☐	☒	For null hypothesis testing, the test statistic (e.g. F, t, r) with confidence intervals, effect sizes, degrees of freedom and P value noted<br>*Give P values as exact values whenever suitable.*
☒	☐	For Bayesian analysis, information on the choice of priors and Markov chain Monte Carlo settings
☒	☐	For hierarchical and complex designs, identification of the appropriate level for tests and full reporting of outcomes
☐	☒	Estimates of effect sizes (e.g. Cohen's *d*, Pearson's *r*), indicating how they were calculated

Our web collection on statistics for biologists contains articles on many of the points above.

Software and code

Policy information about availability of computer code

Data collection	For MS imaging: flexImaging 7.2, TimsControl 4.1;<br>For LC-MS/MS acquisition: Thermo Scientific Xcalibur 4.2 with Foundation 3.1 SP5, Orbitrap Fusion Lumos Tune Application 3.1, Thermo Scientific SII for Xcalibur 1.4
Data analysis	Compass Data Analysis Version 6.1, LipidSearch5 (Thermo Fisher Scientific), SCiLS Lab 2023c Pro, fiji imagej 1.54f and MATLAB R2024B, GlycoWorkbench v2.0, Perseus v2.1.3.0, FragPipe based MSFragger v4.0, Compound Discoverer 3.3 SP2<br>The code for the quantification of tissue expansion non-uniformity is available in Supporting Information materials as Supplementary Note 3.

For manuscripts utilizing custom algorithms or software that are central to the research but not yet described in published literature, software must be made available to editors and reviewers. We strongly encourage code deposition in a community repository (e.g. GitHub). See the Nature Portfolio guidelines for submitting code & software for further information.

Data

Policy information about availability of data

All manuscripts must include a data availability statement. This statement should provide the following information, where applicable:

- Accession codes, unique identifiers, or web links for publicly available datasets
- A description of any restrictions on data availability
- For clinical datasets or third party data, please ensure that the statement adheres to our policy

All the liquid chromatography-mass spectrometry raw files have been deposited into MassIVE (dataset identifier MSV000096466, ftp://massive.ucsd.edu/v07/MSV000096466/). All the Source Data for Figures 1-6 and Extended Data Figures 3 and 4 of MALDI-MSI raw files have been deposited into MassIVE (dataset identifier MSV000097036, ftp://massive.ucsd.edu/v09/MSV000097036/).
GlyGen (http://www.glygen.org) and GlycoWorkbench (https://code.google.com/archive/p/glycoworkbench/).

Human research participants

Policy information about studies involving human research participants and Sex and Gender in Research.

Reporting on sex and gender	N/A
Population characteristics	N/A
Recruitment	N/A
Ethics oversight	N/A

Note that full information on the approval of the study protocol must also be provided in the manuscript.

Field-specific reporting

Please select the one below that is the best fit for your research. If you are not sure, read the appropriate sections before making your selection.

☒ Life sciences ☐ Behavioural & social sciences ☐ Ecological, evolutionary & environmental sciences

For a reference copy of the document with all sections, see nature.com/documents/nr-reporting-summary-flat.pdf

Life sciences study design

All studies must disclose on these points even when the disclosure is negative.

Sample size	All experiments were performed with three samples. As the study is methodological proof of principle, no differences were observed between samples, thus not more samples were included.
Data exclusions	No data was excluded from the analyses.
Replication	Three tissue sections imaged per sample. The number of replicates for each specific experiment is indicated throughout the manuscript text, figure legends and methods. All attempts of replication were successful, no areas or samples were discarded.
Randomization	NA, no groups involved.
Blinding	Samples were anonimised and analysed as such.

Reporting for specific materials, systems and methods

We require information from authors about some types of materials, experimental systems and methods used in many studies. Here, indicate whether each material, system or method listed is relevant to your study. If you are not sure if a list item applies to your research, read the appropriate section before selecting a response.

Materials & experimental systems

n/a	Involved in the study	
☐	☒	Antibodies
☐	☒	Eukaryotic cell lines
☒	☐	Palaeontology and archaeology
☐	☒	Animals and other organisms
☒	☐	Clinical data
☒	☐	Dual use research of concern

Methods

n/a	Involved in the study	
☒	☐	ChIP-seq
☒	☐	Flow cytometry
☒	☐	MRI-based neuroimaging

Antibodies

Antibodies used	GLUT1(SLC2A1) ANTIBODY Probe (AmberGen, AP1001199, 2.5 µg/mL final concentration)<br>MBP(Myelin Basic Protein) ANTIBODY Probe (AmberGen, AP1001200, 2.5 µg/mL final concentration)<br>NF-L(Neurofilament Light) ANTIBODY Probe (AmberGen, AP1001205, 2.5 µg/mL final concentration)<br>PVALB(Parvalbumin) ANTIBODY Probe (AmberGen, AP1001203, 2.5 µg/mL final concentration)<br>SYN-I(Syanpsin I) ANTIBODY Probe (AmberGen, AP1001204, 2.5 µg/mL final concentration)
Validation	All antibodies were selected based on thorough validation by the selling companies for specificity and validated use for tissue IHC. Antibodies were further tested by multiplex imaging mass as shown in Gargey, et al, Highly multiplexed immunohistochemical maldi-ms imaging of biomarkers in tissues, J. Am. Soc. Mass Spectrom. 2021, 32, 4, 977–988 (https://pubs.acs.org/doi/10.1021/jasms.0c00473).<br>GLUT1(SLC2A1) ANTIBODY Probe: MALDI-IHC Ambergen (https://ambergen.com/) and Gargey, et al, J. Am. Soc. Mass Spectrom. 2021, 32, 4, 977–988 (https://pubs.acs.org/doi/10.1021/jasms.0c00473);<br>MBP(Myelin Basic Protein) ANTIBODY Probe: MALDI-IHC Ambergen (https://ambergen.com/) and Gargey, et al, J. Am. Soc. Mass Spectrom. 2021, 32, 4, 977–988 (https://pubs.acs.org/doi/10.1021/jasms.0c00473);<br>NF-L(Neurofilament Light) ANTIBODY Probe: MALDI-IHC Ambergen (https://ambergen.com/) and Gargey, et al, J. Am. Soc. Mass Spectrom. 2021, 32, 4, 977–988 (https://pubs.acs.org/doi/10.1021/jasms.0c00473);<br>PVALB(Parvalbumin) ANTIBODY Probe: MALDI-IHC Ambergen (https://ambergen.com/) and Gargey, et al, J. Am. Soc. Mass Spectrom. 2021, 32, 4, 977–988 (https://pubs.acs.org/doi/10.1021/jasms.0c00473);<br>SYN-I(Syanpsin I) ANTIBODY Probe: MALDI-IHC Ambergen (https://ambergen.com/) and Gargey, et al, J. Am. Soc. Mass Spectrom. 2021, 32, 4, 977–988 (https://pubs.acs.org/doi/10.1021/jasms.0c00473);

Eukaryotic cell lines

Policy information about cell lines and Sex and Gender in Research

Cell line source(s)	B78(B78-D14, GD2+) melanoma cells (from Ralph Reisfeld (Scripps Research Institute)).
Authentication	Cell authentication was performed per ATCC guidelines using morphology, growth curves and Mycoplasma testing within 6 months of use and routinely thereafter.
Mycoplasma contamination	All cell lines tested negative for contamination with mycoplasma.
Commonly misidentified lines<br>(See ICLAC register)	No commonly misidentified cell lines were used.

Animals and other research organisms

Policy information about studies involving animals; ARRIVE guidelines recommended for reporting animal research, and Sex and Gender in Research

Laboratory animals	C57BL/6N female, 6 to 8 months old mice. Mice were housed in facilities with a standard light-dark cycle, humidity of 50% at 24 °C.
Wild animals	This study did not involve wild animals.
Reporting on sex	The sex- and gender-based analysis is not relevant to the study.
Field-collected samples	This study did not involve samples collected from the field.
Ethics oversight	Animal care and experimental procedures were performed with approval from the Institutional Animal Care and Use Committees from the University of Wisconsin-Madison and Howard Hughes Medical Institute (Janelia Research Campus).

Note that full information on the approval of the study protocol must also be provided in the manuscript.
